# Synthesis of 4-Substituted-1,2-Dihydroquinolines by Means of Gold-Catalyzed Intramolecular Hydroarylation Reaction of *N*-Ethoxycarbonyl-*N*-Propargylanilines

**DOI:** 10.3390/molecules26113366

**Published:** 2021-06-02

**Authors:** Antonio Arcadi, Andrea Calcaterra, Giancarlo Fabrizi, Andrea Fochetti, Antonella Goggiamani, Antonia Iazzetti, Federico Marrone, Vincenzo Marsicano, Giulia Mazzoccanti, Andrea Serraiocco

**Affiliations:** 1 Dipartimento di Scienze Fisiche e Chimiche, Università degli Studi di L’Aquila, 67100 Coppito, Italy; antonio.arcadi@univaq.it (A.A.); vincenzo.marsicano@graduate.univaq.it (V.M.); 2 Dipartimento di Chimica e Tecnologie del Farmaco, Dipartimento di Eccellenza 2018–2022, Sapienza Università di Roma, 00185 Rome, Italy; andrea.calcaterra@uniroma1.it (A.C.); giancarlo.fabrizi@uniroma1.it (G.F.); andrea.fochetti@uniroma1.it (A.F.); federico.marrone@uniroma1.it (F.M.); giulia.mazzoccanti@uniroma1.it (G.M.); andrea.serraiocco@uniroma1.it (A.S.)

**Keywords:** gold catalysis, intramolecular hydroarylation, 1,2-dihydroquinolines

## Abstract

An alternative Au(I)-catalyzed synthetic route to functionalized 1,2-dihydroquinolines is reported. This novel approach is based on the use of *N*-ethoxycarbonyl protected-*N*-propargylanilines as building blocks that rapidly undergo the IMHA reaction affording the 6-*endo* cyclization product in good to high yields. In the presence of *N*-ethoxycarbonyl-*N*-propargyl-*meta-*substituted anilines, the regiodivergent cyclization at the *ortho*-/*para*-position is achieved by the means of catalyst fine tuning.

## 1. Introduction

**Scheme 1:**
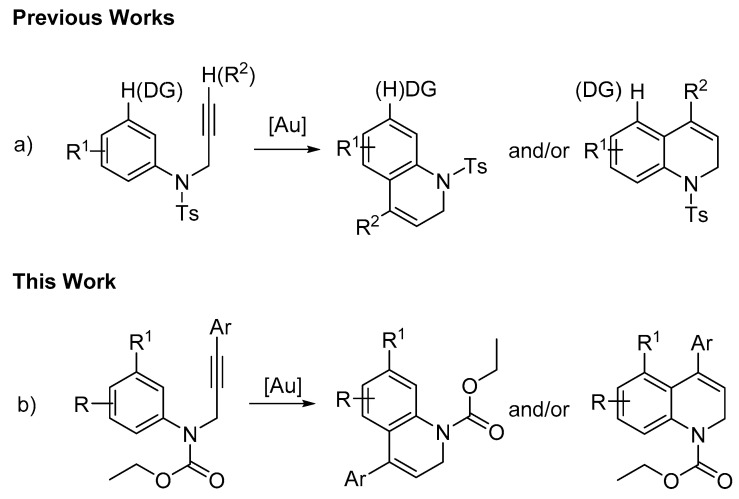
Gold(I)-catalyzed IMHA of *N*-tosyl- (**a**) vs. *N*-ethoxy carbonyl-*N*-propargylanilines (**b**).

4-Substituted-1,2-dihydroquinolines represent key structural units in a variety of naturally occurring products/pharmaceuticals and are used as building blocks in organic synthesis [1,2,3,4,5]. Many methods for the synthesis of functionalized 1,2-dihydroquinolines are known, [1], but due to their pharmaceutical relevance, the development of practical approaches using mild reaction conditions remains an active research area [6,7,8,9,10,11]. Among them, the transition metal-catalyzed as well as the metal-free mediated intramolecular hydroarylation (IMHA) reactions involving the activation of the *N*-substituted-*N*-propargyl anilines carbon–carbon triple bond by using an electrophilic source have been extensively used [12,13]. In particular, the synthetic potential of gold catalysis in the IMHA of *N*-tosyl-*N*-propargylanilines was explored and the corresponding 4-substituted-1,2-dihydroquinoline derivatives were efficiently isolated (Scheme 1a) [14,15,16]. Alternatively these latter products can be obtained by the sequential catalyzed IMHA/Pd-catalyzed cross-coupling of 3-bromo-2-propynyl-*N*-tosylanilines, which afforded the corresponding 4-substituted-1,2-dihydroquinoline derivatives [17,18]. However, the behavior of the substituent attached to *N*-propargylaniline nitrogen has a significant impact on the reaction outcome. While *N*-propargylanilines bearing the more easily removable 2-nitrobenzenesulfonyl (Ns) nitrogen protecting group underwent the gold-catalyzed IMHA to give the corresponding dihydroquinoline in good yield, subjection of the *N*-Boc protected derivatives under the same reaction conditions afforded the divergent formation of an oxazolidinone derivative as the exclusive product [16]. Moreover, 1-azaspirotrienone derivatives were produced exclusively instead of the expected dihydroquinolines when *N*-(4-methoxyphenyl)-*N*-(3-substituted-2-propyn-1-yl)triflamides were reacted with 2 equiv. of ICl in CH_2_Cl_2_ at −78 °C for 0.5 [19]. As part of our ongoing interest on the development of efficient atom-economical routes of heterocycles by means of gold-catalyzed IMHA [20,21,22], we envisaged that the introduction of the more suitable ethyl carbamate protecting group could allow for some of the drawbacks of the previously reported gold-catalyzed IMHA of *N*-substituted-*N*-propargylanilines (Scheme 1b) to be overcome. The carbamate motif, indeed, in addition to being widely known as excellent protecting groups for amines in organic synthesis, has received a great deal of attention in drug design and medicinal chemistry for its application in the construction of drugs and prodrugs [23].

Herein, we report the results of our investigations.

## 2. Results and Discussion

We started our study by examining the transformation of the *N*-ethoxycarbonyl-*N*-propargylaniline **1a** into **2a** under different reaction conditions. The results of this preliminary screening are summarized in Table 1.

As shown, the IMHA of **1a** occurred in almost quantitative yield in the presence of the commercially available JohnPhosAu(MeCN)SbF_6_ catalyst (4 mol %) in anhydrous DCM at 80 °C (Table 1, entry 3) [15,16]. 

**Figure 1:**
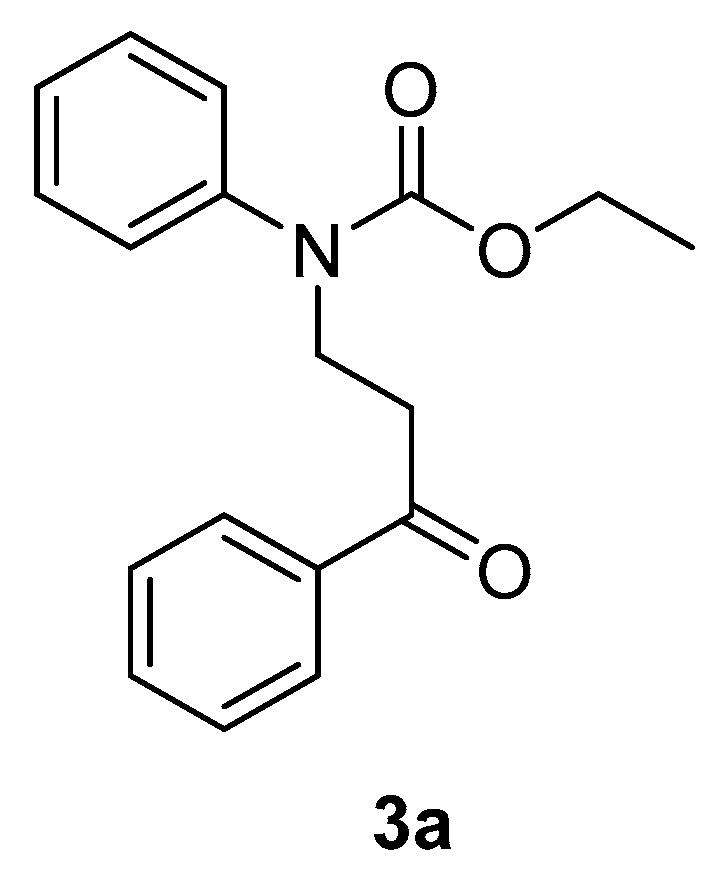
Structure of the hydration derivative **3a**.

About the same result was obtained using the catalytic system JPAuCl/AgNTf_2_ (Table 1, entry 6) while slightly poorer results were observed when CHCl_3_ was used as the solvent instead of DCM (Table 1, entries 4, 5). In this latter solvent, the hydration derivative ethyl 3-oxo-3-phenylpropyl(phenyl)carbamate **3a** was isolated to some extent (Figure 1). 

In contrast with the good efficiency showed by NaAuCl_4_·2H_2_O in the sequential alkylation/gold-catalyzed annulation reactions of anilines with propargylic bromide derivatives providing quinoline scaffolds in ethanol [24], this gold salt was ineffective as the catalyst of the IMHA of *N*-ethoxycarbonyl-*N*-propargylaniline **1a,** affording only the formation of the hydration product **3a** in good yield (Table 1, entry 2) [25]. Starting material **1a** was recovered in almost quantitative yield when PtCl_2_ was used as the catalyst in ethanol (Table 1, entry 1) [26]. 

**Scheme 2:**
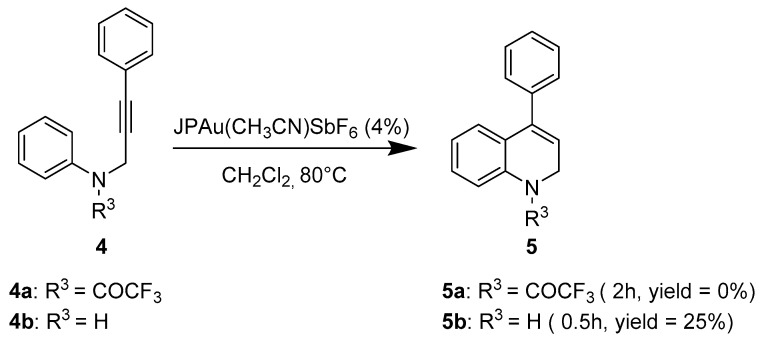
JPAu(CH_3_CN)SbF_6_ catalyzed IMHA of *N*-propargylaniline derivatives **4a** and **4b** ^a^. ^a^ Compound **4b** was recovered in almost quantitative yield when the reaction was carried out in the absence of the catalyst.

Then, to briefly explore the influence of the protecting group on the reaction outcome, we used the optimized reaction condition for the cyclization of the *N*-propargylaniline derivatives **4a** and **4b** (Scheme 2).

**Figure 2:**
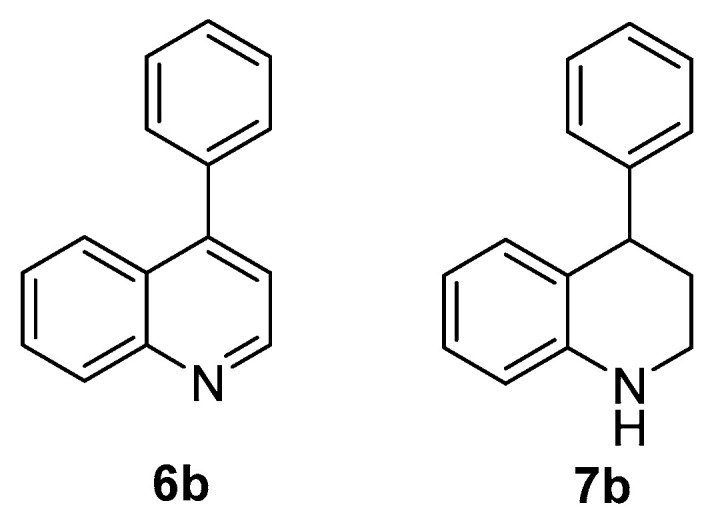
Structure of the 4-phenylquinoline **6b** and 4-phenyl-1,2,3,4-tetrahydroquinoline **7b** obtained from compound **4b** in the condition reported in Scheme 2.

As shown by the results reported in Scheme 2, the *N*-trifluoroacetyl-*N*-propargylaniline derivative failed to undergo the desired gold-catalyzed IMHA to give the corresponding dihydroquinoline **5a** in the presence of 4 mol % of JPAu(CH_3_CN)SbF_6_ in DCM at 80 °C. Interestingly, under the same reaction conditions, the simple *N*-(3-phenylprop-2-yn-1-yl)aniline **4b** underwent a complete gold-catalyzed IMHA, but the 4-phenyl-1,2-dihydroquinoline **5b** (25% yield) was prone to be partially oxidized under the reaction conditions to give the corresponding 4-phenylquinoline **6b** (56% yield). The partial oxidation of **5b** to **6b** occurs even under a nitrogen atmosphere. Furthermore, we observed the formation of **7b**, which was isolated in 7% of yield (see Figure 2) [27].

Subsequently, we continued to establish the scope and the generality of gold(I) catalyzed-IMHA reactions of aryl-substituted *N*-ethoxycarbonyl-*N*-propargylanilines **1** in terms of ring substitution. The utilization of electron-deficient substrates and the control of the regioselectivity of substituted aromatics remain challenges of gold(I) catalyzed-IMHA reactions of aryl-substituted *N*-propargylanilines. To that end, a range of readily accessible derivatives **1a**–**j** were prepared and then subjected to the IMHA in CH_2_Cl_2_ at 80 °C in the presence of the JohnPhosAu(CH_3_CN)SbF_6_ as the catalyst. The outcomes of such studies are shown in Table 2. The 4-arylsubstituted-1,2-dihydroquinoline derivatives **2** were isolated in high yields both when the electron donating –OMe group or the strong withdrawing –COOMe were introduced into the *para-*position of the aromatic ring attached to the alkyne (Table 2, entries 2, 3). Conversely, the introduction of substituents onto the aromatic ring attached to the nitrogen moiety had a different pronounced effect according to their electronic features. The formation of the target 4-aryl-1,2-dihydroquinoline derivative **2** efficiently occurred by the introduction of an electron-donating group on the phenyl ring *para* to the nitrogen and in the *para* position of both aromatic rings of the starting aryl-substituted propargylic aniline derivatives (Table 2, entries 4–6). Moreover, the IMHA was also allowed in almost quantitative yield in the presence of the –Me group on the phenyl ring *para* to the nitrogen and of a withdrawing carbonyl in the *para* position of the other aryl group (Table 2, entry 7). In absolute agreement with considerations of the positive effect of electronic releasing groups on the aromatic ring attached to the nitrogen on the gold-catalyzed IMHA of substrate **1**, substrate **1h** bearing two methyl groups on the same benzene nucleus was smoothly converted to the corresponding 1,2-dihydroquilonine **2h** in about quantitative yield either by the gold-catalyzed IMHA (Table 2, entry 8). Substrate **1i**, possessing a Cl-substituent on the same aromatic ring, cyclized as expected to afford the corresponding dihydroquinoline derivative **2i** in moderate yield (Table 2, entry 9). The formation of the IMHA products occurred only in low yield in the presence of the strong electron-withdrawing CF_3_-substituent probably due to the poorer coordination of the alkyne moiety with the gold catalyst (Table 2, entry 10). 

With regard to the regiochemical outcome, the *meta*-substituted derivatives **1k**–**n** mainly underwent the *para*-position cyclization to give the corresponding 1,2-dihydroquinolines **2k**–**n** in the presence of JohnPhosAu(CH_3_CN)SbF_6_ (catalyst **A**). Fine tuning factors such as valency state, counterion, and auxiliary ligand in homogeneous gold catalysis is imperative in controlling the product divergence [28]. Indeed, for compounds **1k**–**l,** the *para*-position cyclization was revealed to be enhanced in the presence of catalyst **A′** bearing NTf_2_ as counterion (catalyst **A′**, entries 2 and 6). The electron-rich tri-isopropylphenyl ring on the ligand and the slightly more strongly coordinated NTf_2_^−^ jointly lower the electrophilicity of the gold center. On the other hand, the regiodivergent cyclization to the sterically hindered *ortho*-position to give the regioisomeric 1,2-dihydroquinolines **2′k**–**n** resulted governed by the electron-deficient ligand features, according to the literature (Table 3) [29].

**Figure 3:**
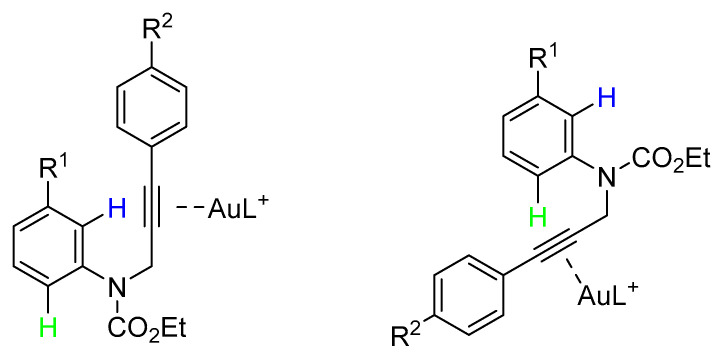
Ligand control of *ortho*/*para* site-selectivity.

Very likely, the control of *ortho/para* site-selectivity in these substrates is the result of the different coordination modes of the gold catalyst influenced by sterics and electronics of the auxiliary ligand. The prowess of electron-rich bulk ligands in pushing the π-system toward the *para* C–H bond through a Au(I)-bicoordinate activation was also explored in the 6-*endo*-*dig* gold catalyzed hydroarylation of functionalized *N*-aryl alkynamides (Figure 3) [30].

**Scheme 3:**
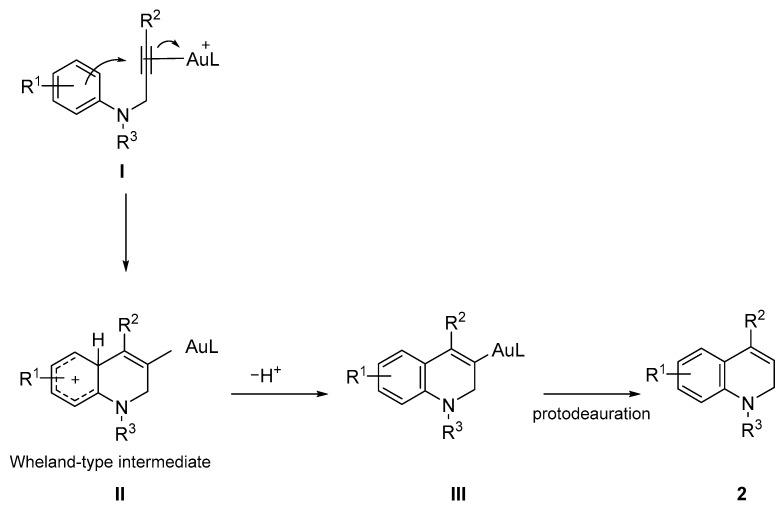
Proposed Friedel–Crafts mechanism for the Au(I)-catalyzed cyclization of *N*-substituted-*N*-propargylanilines.

Indeed, according to the literature [13], the gold catalyzed IMHA proceeds through a Friedel–Crafts type mechanism: η^2^-coordination of alkyne moiety affords complex **I**, which undergoes an electrophilic aromatic substitution to give the Wheland-type intermediate **II**. This latter, after aromatization and protodeauration would give the product **2**. The proposed mechanism is outlined in the Scheme 3.

## 3. Materials and Methods

### 3.1. General Information

All the commercially available reagents, catalysts, bases, and solvents were used as purchased without further purification. Reaction products **2a**–**e** and **2g**–**h** were filtered on a pad of SiO_2_ using AcOEt, while reaction products **2f**, **2i** and **2j** were purified by chromatography on SiO_2_ (25–40 μm), eluting with *n*-hexane/AcOEt mixtures. Reaction products **2k/2′k**–**2o/2′o** were obtained as isomeric mixtures by filtration on a pad of SiO_2_ using AcOEt to eliminate the catalysts before calculating the isomeric ratio by ^1^H NMR. When possible, to obtain suitable NMR spectra of each compound, the isomeric mixtures were further purified by semi-preparative HPLC under normal phase condition using a Nucleodur 100–5 column (762,007.100) and eluting with *n*-hexane/AcOEt mixtures. ^1^H NMR (400.13 MHz), ^13^C NMR (100.6 MHz), and ^19^F spectra (376.5 MHz) were recorded with a Bruker Avance 400 spectrometer. Splitting patterns were designed as s (singlet), d (doublet), t (triplet), q (quartet), m (multiplet), or bs (broad singlet). IR spectra were recorded with a Jasco FT/IR-430 spectrometer. HRMS were recorded with an Orbitrap Exactive Mass spectrometer with ESI source. Melting points were determined with a Büchi B-545 apparatus and are uncorrected. 

### 3.2. Synthetic Procedures

#### 3.2.1. Preparation of Substrates 1 

Substrates were prepared as described in the Supplementary Materials.

#### 3.2.2. Preparation of Derivatives 2: Typical Procedure for the Preparation of the Ethyl 4-Phenylquinoline-1(2H)-Carboxylate **2a**

A Carousel Tube Reactor (Radley Discovery Technology) equipped with a magnetic stirring bar was charged with ethyl phenyl(3-phenylprop-2-yn-1-yl)carbamate **1a** (97.8 mg, 0.35 mmol, 1 equiv.), CH_2_Cl_2_ (2 mL), JohnPhosAu(MeCN)SbF_6_ (10.8 mg, 0.014 mmol, 0.04 equiv.), and sealed. Then, the reaction mixture was stirred at 80 °C and monitored by TLC until the disappearance of the starting material. After 1 h, the obtained mixture was cooled at room temperature and concentrated under reduced pressure. The residue was filtered on a pad of SiO_2_ to afford 97.1 mg of ethyl 4-phenylquinoline-1(2*H*)-carboxylate **2a** (99% yield).

Compound **2a:** yield: 99% (97.1 mg); yellow oil; IR (neat): 2912, 1707, 1380 cm^−1^; ^1^H NMR (400.13 MHz) (CDCl_3_): δ = 7.69 (d, *J* = 7.4 Hz, 1H), 7.45–7.36 (m, 5H), 7.31–7.27 (m, 1H), 7.12–7.05 (m, 2H), 6.07 (t, *J* = 4.5 Hz, 1H), 4.51 (d, *J* = 4.5 Hz, 2H), 4.33 (q, *J* = 7.1 Hz, 2H), 1.39 (t, *J* = 7.1 Hz, 3H); ^13^C NMR (100.6 MHz) (CDCl_3_): δ = 154.2 (q), 138.9 (q), 137.3 (q), 129.3 (q), 128.8 (CH), 128.5 (CH), 127.9 (CH), 127.7 (CH), 126.1 (CH), 124.2 (CH), 124.1 (CH), 123.4 (CH), 62.3 (CH_2_), 43.1 (CH_2_), 14.7 (CH_3_); MS (EI ion source): *m*/*z* (%) = 279 (18, [M^+^]), 206 (100), 204 (62), 102 (21); HRMS: *m*/*z* [M + Na]^+^ calcd. for C_18_H_17_NO_2_Na: 302.1152; found: 302.1152.

### 3.3. Characterization Data

#### 3.3.1. Characterization Data of Compound **1a**–**o**

Characterization data of substrate **1** are reported in the Supplementary Materials.

#### 3.3.2. Characterization Data of Compound **2b**–**j**, **2l**–**o**, **2′l**–**2′o**

Compound **2b**. Yield: 82% (88.9 mg); yellow oil; IR (neat): 2980, 1699, 1608, 1510, 1051 cm^−1^; ^1^H NMR (400.13 MHz) (CDCl_3_): δ = 7.56 (bd, *J* = 7.7 Hz, 1H), 7.19 (d, *J* = 8.8 Hz, 2H), 7.18–7.15 (m, 1H), 7.01 (dd, *J*_1_ = 7.8 Hz, *J*_2_ = 1.7 Hz, 1H), 6.95 (dt, *J*_1_ = 7.2 Hz, *J*_2_ = 1.2 Hz, 1H), 6.84 (d, *J* = 8.8 Hz, 2H), 5.91 (t, *J* = 4.5 Hz, 1H), 4.37 (d, *J* = 4.5 Hz, 2H), 4.20 (q, *J* = 7.1 Hz, 2H), 3.76 (s, 3H), 1.26 (t, *J* = 7.1 Hz, 3H); ^13^C NMR (100.6 MHz) (CDCl_3_): δ = 159.4 (q), 154.2 (q), 138.4 (q), 137.4 (q), 131.3 (q), 130.0 (CH), 129.5 (q), 127.6 (CH), 126.1 (CH), 124.15 (CH), 124.08 (CH), 122.5 (CH), 113.9 (CH), 62.2 (CH_2_), 55.4 (CH_3_), 43.1 (CH_2_), 14.7 (CH_3_); MS (EI ion source): *m*/*z* (%) = 309 (47, [M^+^]), 280 (93), 236 (100), 221 (21), 192 (20); HRMS: *m*/*z* [M + Na]^+^ calcd. for C_19_H_19_NO_3_Na: 332.1257; found: 332.1261.

Compound **2c**. Yield: 99% (111.3 mg); white solid; m.p. = 110–111 °C; IR (neat): 2977, 1698, 1604, 1484 cm^−1^; ^1^H NMR (400.13 MHz) (CDCl_3_): δ = 7.98 (d, *J* = 8.5 Hz, 2H), 7.67–7.65 (m, 1H), 7.45 (d, *J* = 8.5 Hz, 2H), 7.30–7.25 (m, 1H), 7.06–6.99 (m, 2H), 6.10 (t, *J* = 4.6 Hz, 1H), 4.49 (d, *J* = 4.6 Hz, 2H), 4.28 (q, *J* = 7.1 Hz, 2H), 2.63 (s, 3H), 1.34 (t, *J* = 7.1 Hz, 3H); ^13^C NMR (100.6 MHz) (CDCl_3_): δ = 197.8 (q), 154.2 (q), 143.9 (q), 138.3 (q), 137.4 (q), 136.6 (q), 129.1 (CH), 128.7 (CH), 128.1 (CH), 126.0 (CH), 124.6 (CH), 124.4 (CH), 120.6 (q), 62.4 (CH_2_), 43.1 (CH_2_), 26.8 (CH_3_), 14.7 (CH_3_); MS (EI ion source): *m*/*z* (%) = 321 (27, [M^+^]), 292 (71), 248 (100), 205 (28); HRMS: *m*/*z* [M + H]^+^ calcd. for C_20_H_20_NO_3_: 322.1438; found: 322.1431.

Compound **2d**. Yield: 82% (88.6 mg); pale yellow oil; IR (neat): 2980, 1702, 1491, 1382 cm^−1^; ^1^H NMR (400.13 MHz) (CDCl_3_): δ = 7.56–7.53 (m, 1H), 7.40–7.33 (m, 5H), 6.81 (dd, *J*_1_ = 8.9 Hz, *J*_2_ = 2.9 Hz, 1H), 6.60 (d, *J* = 2.9 Hz, 1H), 6.06 (t, *J* = 4.5 Hz, 1H), 4.44 (d, *J* = 4.5 Hz, 2H), 4.26 (q, *J* = 7.1 Hz, 2H), 3.69 (s, 3H), 1.33 (t, *J* = 7.1 Hz, 3H); ^13^C NMR (100.6 MHz) (CDCl_3_): δ = 156.2 (q), 154.4 (q), 138.9 (q), 138.8 (q), 130.51 (q), 130.47 (q), 128.8 (CH), 128.5 (CH), 127.9 (CH), 125.2 (CH), 113.0 (CH), 111.6 (CH), 62.1 (CH_2_), 55.6 (CH_3_), 43.2 (CH_2_), 14.7 (CH_3_); MS (EI ion source): *m*/*z* (%) = 309 (21, [M^+^]), 280 (24), 236 (100), 193 (36), 165 (19), 63 (30); HRMS: *m*/*z* [M + H]^+^ calcd. for C_19_H_20_NO_3_: 310.1438; found: 310.1443.

Compound **2e**. Yield: 99% (101.4 mg); orange oil; IR (neat): 2981, 1697, 1493, 1378 cm^−1^; ^1^H NMR (400.13 MHz) (CDCl_3_): δ = 7.53–7.52 (m, 1H), 7.42–7.34 (m, 5H), 7.07 (dd, *J*_1_ = 8.3 Hz, *J*_2_ = 1.4 Hz, 1H), 6.86 (bd, *J* = 1.4 Hz, 1H), 6.02 (t, *J* = 4.5 Hz, 1H), 4.45 (d, *J* = 4.5 Hz, 2H), 4.28 (q, *J* = 7.1 Hz, 2H), 2.24 (s, 3H), 1.34 (t, *J* = 7.1 Hz, 3H); ^13^C NMR (100.6 MHz) (CDCl_3_): δ = 154.2 (q), 139.1 (q), 139.0 (q), 134.8 (q), 133.8 (q), 129.1 (q), 128.8 (CH), 128.5 (CH), 128.4 (CH), 127.8 (CH), 126.5 (CH), 124.0 (CH), 123.5 (CH), 62.2 (CH_2_), 43.1 (CH_2_), 21.1 (CH_3_), 14.7 (CH_3_); MS (EI ion source): *m*/*z* (%) = 293 (39, [M^+^]), 264 (49), 220 (100), 204 (55), 63 (29); HRMS: *m*/*z* [M + H]^+^ calcd. for C_19_H_20_NO_2_: 294.1489; found: 294.1491.

Compound **2f**. Yield: 68% (77.2 mg); yellow oil; IR (neat): 2980, 2836, 1702, 1608, 1509, 1463 cm^−1^; ^1^H NMR (400.13 MHz) (CDCl_3_): δ = 7.53–7.51 (m, 1H), 7.28 (d, *J* = 8.8 Hz, 2H), 7.07 (dd, *J*_1_ = 8.3 Hz, *J*_2_ = 1.6 Hz, 1H), 6.93 (d, *J* = 8.8 Hz, 2H), 6.89 (bd, *J* = 1.6 Hz, 1H), 5.97 (t, *J* = 4.5 Hz, 1H), 4.43 (d, *J* = 4.5 Hz, 2H), 4.27 (q, *J* = 7.1 Hz, 2H), 3.85 (s, 3H), 2.24 (s, 3H), 1.34 (t, *J* = 7.1 Hz, 3H); ^13^C NMR (100.6 MHz) (CDCl_3_): δ = 159.3 (q), 154.2 (q), 138.5 (q), 134.8 (q), 133.7 (q), 131.4 (q), 129.9 (CH), 129.3 (CH), 128.3 (CH), 126.5 (CH), 123.9 (CH), 122.7 (q), 113.9 (CH), 62.1 (CH_2_), 55.4 (CH_3_), 43.1 (CH_2_), 21.1 (CH_3_), 14.7 (CH_3_); MS (EI ion source): *m*/*z* (%) = 323 (43, [M^+^]), 294 (87), 250 (100), 235 (20), 207 (17); HRMS: *m*/*z* [M + H]^+^ calcd. for C_20_H_22_NO_3_: 324.1594; found: 324.1597.

Compound **2g**. Yield: 96% (112.4 mg); orange wax; IR (neat): 2980, 2243, 1705, 1596, 1494 cm^−1^; ^1^H NMR (400.13 MHz) (CDCl_3_): δ = 7.99 (d, *J* = 8.4 Hz, 2H), 7.54–7.52 (m, 1H), 7.45 (d, *J* = 8.4 Hz, 2H), 7.11–7.07 (m, 1H), 6.79 (bd, *J* = 1.4 Hz, 1H), 6.08 (t, *J* = 4.4 Hz, 1H), 4.46 (d, *J* = 4.4 Hz, 2H), 4.27 (q, *J* = 7.1 Hz, 2H), 2.64 (s, 3H), 2.23 (s, 3H), 1.34 (t, *J* = 7.1 Hz, 3H); ^13^C NMR (100.6 MHz) (CDCl_3_): δ = 197.9 (q), 154.2 (q), 144.0 (q), 138.3 (q), 136.5 (q), 134.8 (q), 133.9 (q), 129.6 (CH), 129.0 (CH), 128.7 (CH), 128.6 (CH), 128.5 (q), 126.3 (CH), 124.1 (CH), 62.3 (CH_2_), 43.1 (CH_2_), 26.8 (CH_3_), 21.1 (CH_3_), 14.7 (CH_3_); MS (EI ion source): *m*/*z* (%) = 351 (M^+^, 31), 322 (50), 278 (14), 157 (46), 134 (100), 114 (62); HRMS: *m*/*z* [M + H]^+^ calcd. for C_21_H_22_NO_3_: 336.1594; found: 336.1598.

Compound **2h**. Yield: 99% (106.3 mg); yellow oil; IR (neat): 2979, 1703, 1608, 1557, 1376, 1271 cm^−1^; ^1^H NMR (400.13 MHz) (CDCl_3_): δ = 7.28–7.14 (m, 6H), 6.68 (s, 1H), 6.01 (t, *J* = 5.2 Hz, 1H), 4.20–4.14 (m, 4H), 2.26 (s, 3H), 1.65 (s, 3H), 1.24 (t, *J* = 7.1 Hz, 3H); ^13^C NMR (100.6 MHz) (CDCl_3_): δ = 153.9 (q), 141.8 (q), 139.9 (q), 139.1 (q), 137.3 (q), 135.3 (q), 129.0 (CH), 128.5 (CH), 127.4 (CH), 127.2 (CH), 125.8 (q), 125.3 (CH), 122.4 (CH), 62.1 (CH_2_), 42.6 (CH_2_), 23.0 (CH_3_), 21.5 (CH_3_), 14.7 (CH_3_); MS (EI ion source): *m*/*z* (%) = 307 (20, [M^+^]), 278 (27), 234 (100), 218 (18); HRMS: *m*/*z* [M + H]^+^ calcd. for C_20_H_22_NO_2_: 308.1645; found: 308.1649.

Compound **2i**. Yield: 56% (61.7 mg); pale yellow oil; IR (neat): 2981, 2847, 1702, 1594, 1481 cm^−1^; ^1^H NMR (400.13 MHz) (CDCl_3_): δ = 7.59 (bd, *J* = 8.1 Hz, 1H), 7.43–7.36 (m, 3H), 7.33–7.30 (m, 2H), 7.21 (dd, *J*_1_ = 8.7 Hz, *J*_2_ = 2.5 Hz, 1H), 7.02 (d, *J* = 2.5 Hz, 1H), 6.06 (t, *J* = 4.5 Hz, 1H), 4.47 (d, *J* = 4.5 Hz, 2H), 4.28 (q, *J* = 7.1 Hz, 2H), 1.34 (t, *J* = 7.1 Hz, 3H); ^13^C NMR (100.6 MHz) (CDCl_3_): δ = 153.9 (q), 138.1 (q), 135.6 (q), 130.7 (q), 129.5 (CH), 128.61 (CH), 128.60 (CH), 128.0 (CH), 127.4 (CH), 125.7 (CH), 125.3 (CH), 124.4 (q), 62.3 (CH_2_), 43.0 (CH_2_), 14.5 (CH_3_). MS (EI ion source): *m*/*z* (%) = 313 (30, [M^+^]), 284 (72), 240 (100), 204 (62), 176 (19); HRMS: *m*/*z* [M + H]^+^ calcd. for C_18_H_17_ClNO_2_: 314.0942; found: 314.0952.

Compound **2j**. Yield: 10% (13.0 mg); colorless oil; IR (eat): 2919, 2848, 1710, 1609, 1382, 1051 cm^−1^; ^1^H NMR (400.13 MHz) (CDCl_3_): δ = 7.77 (bd, *J* = 8.5 Hz, 1H), 7.49 (dd, *J*_1_ = 8.6 Hz, *J*_2_ = 1.6 Hz, 1H), 7.33 (bd, *J* = 1.6 Hz, 1H), 7.25 (d, *J* = 8.8 Hz, 2H), 6.94 (d, *J* = 8.8 Hz, 2H), 6.04 (t, *J* = 4.5 Hz, 1H), 4.48 (d, *J* = 4.5 Hz, 2H), 4.30 (q, *J* = 7.1 Hz, 2H), 3.86 (s, 3H), 1.36 (t, *J* = 7.1 Hz, 3H); ^13^C NMR (100.6 MHz) (CDCl_3_): δ = 159.7 (q), 153.9 (q), 140.4 (q), 137.8 (q), 130.4 (q), 129.9 (q), 129.7 (CH), 126.1 (q, q, *J* = 32.5 Hz), 124.5 (CH, q, *J* = 3.7 Hz), 124.20 (q, q, *J* = 270.3 Hz), 124.19 (CH), 123.7 (CH), 123.1 (CH, q, *J* = 3.7 Hz), 114.2 (CH), 62.7 (CH_2_), 55.5 (CH_3_), 43.2 (CH_2_), 14.6 (CH_3_); ^19^F NMR (376.5 MHz) (CDCl_3_): δ = −62.3; MS (EI ion source): *m*/*z* (%) = 377 (26, [M^+^]), 348 (77), 304 (100), 289 (14), 261 (18); HRMS: *m*/*z* [M + Na]^+^ calcd. for C_20_H_18_F_3_NO_3_Na: 400.1131; found: 400.1119.

Isomeric mixture **2k** + **2′k**. Overall yield (catalyst A): 99% (107.2.0 mg); **2k**/**2′k** = 67/33; overall yield (catalyst A′): 99% (107.1 mg); **2k**/**2′k** = 94/6; overall yield (catalyst B): 90% (97.1 mg); **2k**/**2′k** = 44/56; overall yield (catalyst C): 67% (72.7 mg); **2k**/**2′k** = 46/54.

**2k**: orange oil; IR (Neat): 2980, 2243, 1705, 1596, 1494 cm^−1^; ^1^H NMR (400.13 MHz) (CDCl_3_): δ = 7.29–7.21 (m, 6H), 6.90 (d, *J* = 8.7 Hz, 1H), 6.52 (dd, *J*_1_ = 8.7 Hz, *J*_2_ = 2.6 Hz, 1H), 5.80 (t, *J* = 4.5 Hz, 1H), 4.38 (d, *J* = 4.6 Hz, 2H), 4.21 (q, *J* = 7.1 Hz, 2H), 3.74 (s, 3H), 1.27 (t, *J* = 7.1 Hz, 3H); ^13^C NMR (100.6 MHz) (CDCl_3_): δ = 159.1 (q), 154.1 (q), 139.2 (q), 138.7 (q), 138.6 (q), 128.8 (CH), 128.5 (CH), 127.8 (CH), 127.0 (CH), 122.5 (q), 120.4 (CH), 110.2 (CH), 109.7 (CH), 62.3 (CH_2_), 55.5 (CH_3_), 43.3 (CH_2_), 14.7 (CH_3_). MS (EI ion source): *m*/*z* (%) = 309 (0.2, [M^+^]), 235 (100), 220 (17), 204 (29), 191 (24), 165 (15); HRMS: *m*/*z* [M + Na]^+^ calcd. for C_19_H_19_NO_3_Na: 332.1257; found: 332.1260.

**2′k**: colorless oil; IR (neat): 2982, 1708, 1610, 1504, 1466 cm^−1^; ^1^H NMR (400.13 MHz) (CDCl_3_): δ = 7.36–7.24 (m, 6H), 6.69 (d, *J* = 8.9 Hz, 1H), 6.08 (t, *J* = 5.1 Hz, 1H), 4.33 (d, *J* = 5.1 Hz, 2H), 4.29 (q, *J* = 7.2 Hz, 2H), 3.43 (s, 3H), 1.35 (t, *J* = 7.2 Hz, 3H); ^13^C NMR (100.6 MHz) (CDCl_3_): δ = 156.2 (q), 153.9 (q), 141.5 (q), 139.8 (q), 137.8 (q), 128.3 (CH), 127.7 (CH), 126.68 (CH), 126.62 (CH), 124.5 (CH), 118.6 (q), 117.0 (CH), 108.4 (CH), 62.2 (CH_2_), 55.5 (CH_3_), 42.7 (CH_2_), 14.7 (CH_3_); MS (EI ion source): *m*/*z* (%) = 309 (42, [M^+^]), 280 (51), 236 (100), 220 (51), 193 (15); HRMS: *m*/*z* [M + Na]^+^ calcd. for C_19_H_19_NO_3_Na: 332.1257; found: 332.1260.

Isomeric mixture **2l** + **2′l**. Overall yield (catalyst A): 98% (120.4 mg); **2l**/**2′l** = 61/39; overall yield (catalyst A′): 99% (120.9); **2l**/**2′l** = 75/29; overall yield (catalyst B): 99% (121.9 mg); **2l**/**2′l** = 54/46; overall yield (catalyst C): 86% (105.5 mg); **2l**/**2′l** = 54/46; yellow wax; IR (KBr): 3060, 2922, 1680, 1593, 1480, 1232 cm^−1^.

Reported NMR spectra refer to an isomeric mixture **2l** + **2′l** in the ratio 54/46; ^1^H NMR signals were assigned to each specific isomer while ^13^C NMR signals were not assigned.

^1^H NMR (400.13 MHz) (CDCl_3_) (selected signals): δ = 7.89 (d, *J* = 8.4 Hz, 2H **2l**), 7.81 (d, *J* = 8.4 Hz, 2H **2′l**), 7.35 (d, *J* = 8.4 Hz, 2H **2l**), 7.24–7.17 (m, 1H **2l** + 4H **2′l**), 6.84 (d, *J* = 8.6 Hz, 1H **2l**), 6.59 (d, *J* = 9.0 Hz, 1H **2′l**), 6.52 (dd, *J*_1_ = 8.6 Hz, *J*_2_ = 2.6 Hz, 1H **2l**), 6.03 (t, *J* = 5.0 Hz, 1H **2′l**), 5.87 (t, *J* = 4.6 Hz, 1H **2l**), 4.39 (d, *J* = 4.6 Hz, 2H **2l**), 4.25–4.15 (m, 2H **2l** + 4H **2′l**), 3.74 (s, 3H **2l**), 3.32 (s, 3H **2′l**), 2.54 (s, 3H **2l**), 2.53 (s, 3H **2′l**), 1.29–1.23 (m, 3H **2l** + 3H **2′l**); ^13^C NMR (100.6 MHz) (CDCl_3_): δ = 198.1, 197.9, 159.4, 156.0, 154.1, 153.9, 146.7, 144.1, 139.8, 138.7, 138.1, 137.1, 136.6, 135.5, 129.0, 128.8, 128.7, 128.0, 126.93, 126.90, 126.0, 121.9, 121.7, 117.9, 117.1, 110.4, 109.9, 108.2, 62.43, 62.39, 55.6, 55.4, 43.2, 42.7, 26.85, 26.79, 14.8, 14.7; MS (EI ion source): *m*/*z* (%) = 351 (51, [M^+^]), 322 (63), 278 (100), 262 (23), 235 (20), 43 (23); HRMS: *m*/*z* [M + H]^+^ calcd. for C_21_H_22_NO_4_: 352.1543; found: 352.1536.

Isomeric mixture **2m** + **2′m**. Overall yield (catalyst A): 70% (83.0 mg); **2m**/**2′m** = 91/9; overall yield (catalyst A′): 73% (86.5 mg); **2m**/**2′m** = 91/9; overall yield (catalyst B): 83% (98.6 mg); **2m**/**2′m** = 63/37; overall yield (catalyst C): 77% (91.8 mg); **2m**/**2′m** = 51/49.

Compound **2m**. Colorless oil; IR (Neat): 2915, 1711, 1577, 1386, 1244 cm^−1^; ^1^H NMR (400.13 MHz) (CDCl_3_): δ = 7.27–7.24 (m, 3H), 7.00 (d, *J* = 8.6 Hz, 1H), 6.91 (d, *J* = 8.8 Hz, 2H), 6.60 (dd, *J*_1_ = 8.6 Hz, *J*_2_ = 2.6 Hz, 1H), 5.84 (t, *J* = 4.5 Hz, 1H), 4.43 (d, *J* = 4.5 Hz, 2H), 4.28 (q, *J* = 7.1 Hz, 2H), 3.84 (s, 3H), 3.82 (s, 3H), 1.35 (t, *J* = 7.1 Hz, 3H); ^13^C NMR (100.6 MHz) (CDCl_3_): δ = 159.3 (q), 159.0 (q), 154.1 (q), 138.6 (q), 138.2 (q), 131.6 (q), 129.9 (CH), 127.0 (CH), 122.7 (q), 119.6 (CH), 113.9 (CH), 110.2 (CH), 109.6 (CH), 62.2 (CH_2_), 55.5 (CH_3_), 55.4 (CH_3_), 43.2 (CH_2_), 14.7 (CH_3_); MS (EI ion source): *m*/*z* (%) = 339 (1, [M^+^]), 265 (100), 250 (13), 222 (10), 207 (15); HRMS: *m*/*z* [M + K]^+^ calcd. for C_20_H_21_NO_4_K: 378.1102; found: 378.1095.

Compound **2′m**. Colorless oil; IR (neat): 2915, 1694, 1609, 1381, 1239, 1042 cm^−1^; ^1^H NMR (400.13 MHz) (CDCl_3_): δ = 7.26–7.24 (m, 1H), 7.17 (t, *J* = 8.2 Hz, 1H), 7.08 (d, *J* = 8.8 Hz, 2H), 6.75 (d, *J* = 8.8 Hz, 2H), 6.60 (d, *J* = 8.9 Hz, 1H), 5.93 (t, *J* = 5.1 Hz, 1H), 4.20 (d, *J* = 5.1 Hz, 2H), 4.18 (q, *J* = 7.1 Hz, 2H), 3.75 (s, 3H), 3.37 (s, 3H), 1.25 (t, *J* = 7.1 Hz, 3H); ^13^C NMR (100.6 MHz) (CDCl_3_): δ = 158.6 (q), 156.3 (q), 153.9 (q), 140.0 (q), 137.2 (q), 134.1 (q), 128.3 (CH), 127.8 (CH), 123.6 (CH), 118.7 (q), 117.0 (CH), 113.1 (CH), 108.4 (CH), 62.3 (CH_2_), 55.7 (CH_3_), 55.5 (CH_3_), 42.7 (CH_2_), 14.7 (CH_3_); MS (EI ion source): *m*/*z* (%) = 339 (66, [M^+^]), 310 (67), 266 (100), 251 (23); HRMS: *m*/*z* [M + H]^+^ calcd. for C_20_H_22_NO_4_: 340.1543; found: 340.1539.

Isomeric mixture **2n** + **2′n**. Overall yield (catalyst A): 85% (112.9 mg); **2n**/**2′n** = 88/12; overall yield (catalyst A′): 75% (99.7); **2n**/**2′n** = 64/36; overall yield (catalyst B): 99% (131.6 mg); **2n**/**2′n** = 40/60; overall yield (catalyst C): 72% (95.1 mg); **2n**/**2′n** = 19/81.

Compound **2n**: pale yellow solid; m.p. = 140–141 °C; IR (neat): 2930, 1751, 1657, 1583, 1298 cm^−1^; ^1^H NMR (400.13 MHz) (CDCl_3_): δ = 8.27 (bs, 1H), 8.08 (d, *J* = 8.4 Hz, 2H), 7.62 (dd, *J*_1_ = 8.2 Hz, *J*_2_ = 1.7 Hz, 1H), 7.41 (d, *J* = 8.4 Hz, 2H), 7.08 (d, *J* = 8.2 Hz, 1H), 6.21 (t, *J* = 4.5 Hz, 1H), 4.53 (d, *J* = 4.5 Hz, 2H), 4.31 (q, *J* = 7.1 Hz, 2H), 3.94 (s, 3H), 2.60 (s, 3H), 1.37 (t, *J* = 7.1 Hz, 3H); ^13^C NMR (100.6 MHz) (CDCl_3_): δ = 197.3 (q), 166.7 (q), 153.9 (q), 142.8 (q), 137.7 (q), 137.3 (q), 136.4 (q), 132.6, (q) 129.9 (CH), 129.8 (CH), 128.7 (CH), 126.9 (q), 125.9 (CH), 124.4 (CH), 123.9 (CH), 62.5 (CH_2_), 52.2 (CH_3_), 43.0 (CH_2_), 26.7 (CH_3_), 14.5 (CH_3_); MS (EI ion source): *m*/*z* (%) = 379 (67, [M^+^]), 350 (15), 306 (100), 290 (52), 264 (70), 204 (26); HRMS: *m*/*z* [M + Na]^+^ calcd. for C_22_H_21_NO_5_Na: 402.1312; found: 402.1312.

Compound **2′n**: yellow oil; IR (neat): 2919, 1725, 1599, 1268, 1023 cm^−1^; ^1^H NMR (400.13 MHz) (CDCl_3_): δ = 7.90 (d, *J* = 8.5 Hz, 2H), 7.77 (m, 1H), 7.30 (t, *J* = 7.8 Hz, 1H), 7.23–7.19 (m, 3H), 6.21 (t, *J* = 5.2 Hz, 1H), 4.33 (d, *J* = 5.2 Hz, 2H), 4.21 (q, *J* = 7.1 Hz, 2H), 3.84 (s,3 H), 2.00 (s, 3H), 1.27 (t, *J* = 7.1 Hz, 3H); ^13^C NMR (100.6 MHz) (CDCl_3_): δ = 202.2 (q), 166.7 (q), 153.6 (q), 145.4 (q), 139.8 (q), 139.5 (q), 138.0 (q), 129.9 (q), 129.1 (CH), 127.6 (CH), 127.3 (CH), 127.1 (q), 126.7 (CH), 123.9 (CH), 62.5 (CH_2_), 52.1 (CH_3_), 42.4 (CH_2_), 29.1 (CH_3_), 14.5 (CH_3_); MS (EI ion source): *m*/*z* (%) = 379 (58, [M^+^]), 350 (100), 306 (95), 264 (44), 204 (36), 43 (27); HRMS: *m*/*z* [M + Na]^+^ calcd. for C_22_H_21_NO_5_Na: 402.1312; found: 402.1310.

Isomeric mixture **2o** + **2′o**. Overall yield (catalyst A): 88% (112.0 mg); **2o**/**2′o** = 88/12; overall yield (catalyst A′): 90% (114.3); **2o**/**2′o** = 65/35; overall yield (catalyst B): 70% (89.0 mg); **2o**/**2′o** = 33/67; overall yield (catalyst C): 82% (104.4 mg); **2o**/**2′o** = 20/80.

Compound **2o**. Yellow wax; IR (neat): 2982, 1680, 1607, 1556, 1256 cm^−1^; ^1^H NMR (400.13 MHz) (CDCl_3_): δ = 8.27 (bs, 1H), 8.00 (d, *J* = 8.4 Hz, 2H), 7.63 (dd, *J*_1_ = 8.2 Hz, *J*_2_ = 1.7 Hz, 1H), 7.43 (d, *J* = 8.4 Hz, 2H), 7.08 (d, *J* = 8.2 Hz, 1H), 6.22 (t, *J* = 4.6 Hz, 1H), 4.54 (d, *J* = 4.6 Hz, 2H), 4.32 (q, *J* = 7.1 Hz, 2H), 2.64 (s, 3H), 2.60 (s, 3H), 1.37 (t, *J* = 7.1 Hz, 3H); ^13^C NMR (100.6 MHz) (CDCl_3_): δ = 197.8 (q), 197.5 (q), 154.0 (q), 143.1 (q), 137.9 (q), 137.5 (q), 136.9 (q), 136.7 (q), 132.7 (q), 129.1 (CH), 128.9 (CH), 127.2 (CH), 126.1 (CH), 124.6 (CH), 124.0 (CH), 62.7 (CH_2_), 43.3 (CH_2_), 26.9 (CH_3_, 2C), 14.7 (CH_3_); MS (EI ion source): *m*/*z* (%) = 363 (62, [M^+^]), 333 (12), 290 (83), 248 (70), 43 (100); HRMS: *m*/*z* [M + Na]^+^ calcd. for C_22_H_21_NO_4_Na: 386.1363; found: 386.1359.

Compound **2′o**. Yellow wax; IR (neat): 2981, 1682, 1603, 1450, 1376, 1265 cm^−1^; ^1^H NMR (400.13 MHz) (CDCl_3_): δ = 7.89 (d, *J* = 8.6 Hz, 2H), 7.85 (m, 1H) 7.38 (t, *J* = 7.7 Hz, 1H), 7.31–7.27 (m, 3H), 6.28 (t, *J* = 5.2 Hz, 1H), 4.40 (d, *J* = 5.2 Hz, 2H), 4.28 (q, *J* = 7.1 Hz, 2H), 2.59 (s, 3H), 2.07 (s, 3H), 1.34 (t, *J* = 7.1 Hz, 3H); ^13^C NMR (100.6 MHz) (CDCl_3_): δ = 202.3 (q), 197.6 (q), 153.7 (q), 145.8 (q), 139.9 (q), 139.6 (q), 138.1 (q), 136.1 (q), 128.8 (CH), 127.7 (CH), 127.39 (CH), 127.34 (q), 126.9 (CH), 124.1 (CH), 62.6 (CH_2_), 42.5 (CH_2_), 29.3 (CH_3_), 26.7 (CH_3_), 14.6 (CH_3_); MS (EI ion source): *m*/*z* (%) = 363 (26, [M^+^]), 334 (41), 290 (54), 232 (28), 204 (30), 43 (100); HRMS: *m*/*z* [M + Na]^+^ calcd. for C_22_H_21_NO_4_Na: 386.1363; found: 386.1361. 

#### 3.3.3. Characterization Data of Compounds **3a**, **5b**, **6b**, **7b**

Characterization data of the listed compounds are reported in the Supplementary Materials.

## 4. Conclusions

The employment of ethyl carbamate *N*-protecting group represents a viable alternative to the tosyl *N*-protecting group, allowing the efficient synthesis of the corresponding 4-substituted-1,2-dihydroquinolines by means of the gold-catalyzed IMHA reaction. The reaction proceeds with internal alkynes bearing electron-rich and electron-deficient substituents in the benzenes affording only the *6-endo* cyclization product in fair to high yields. Au(I)- catalyzed regiodivergent intramolecular hydroarylation of the *N*-ethoxycarbonyl-*N*-propargyl-*meta-*substituted anilines at the *ortho*- and *para*-position cyclization could be successfully established respectively through fine-tuning electronic and steric effects of the gold complexes ligands. 

## Figures and Tables

**Table 1:**
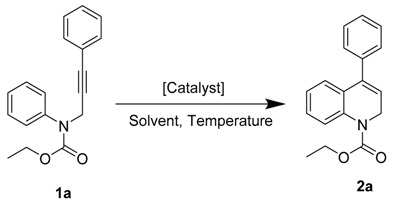
Screening optimal conditions for the IMHA of the *N*-ethoxycarbonyl-*N*-propargylaniline **1a** ^a^.

Entry	Catalyst	T (°C)	Solvent	Time (h)	2a (Yield %)
1	PtCl_2_	80	EtOH	24	- ^b^
2	NaAuCl_4_	80	EtOH	48	- ^c^
3	JPAu(CH_3_CN)SbF_6_	80	DCM ^d^	1	98
4	JPAu(CH_3_CN)SbF_6_	100	CHCl_3_	4.5	70 ^e^
5	JPAu(CH_3_CN)SbF_6_	100	CHCl_3_ ^d^	0.75	86 ^f^
6	JPAuCl/AgNTf_2_	80	CH_2_Cl_2_	3.5	94

^a^ Reactions were carried out on a 0.35 mmol of **1a** in 2 mL of solvent in the presence of 0.014 mmol of the catalyst. ^b^ **1a** was recovered in 30% yield, ethyl 3-oxo-3-phenylpropyl(phenyl)carbamate **3a** was isolated in 35% yield; ^c^ **1a** was recovered in 18% yield, **3a** was isolated in 75% yield; ^d^ Commercial anhydrous solvent; ^e^ **3a** was isolated in 21% yield; ^f^ **3a** was isolated in 7% yield.

**Table 2:**
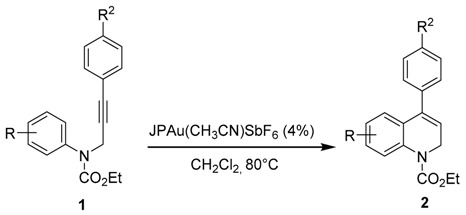
Scope of the gold(I)-catalyzed IMHA of the *N*-ethoxycarbonyl-*N*-propargylanilines **1** ^a^.

Entry	R	R^2^	1	Time (h)	2 (Yield %)
1	H	H	**a**	1	**2a** (98)
2	H	OMe	**b**	18	**2b** (82)
3	H	COMe	**c**	2	**2c** (99)
4	4-OMe	H	**d**	2	**2d** (82)
5	4-Me	H	**e**	1	**2e** (99)
6	4-Me	OMe	**f**	5	**2f** (68) ^b^
7	4-Me	COMe	**g**	1	**2g** (99)
8	3,5-(Me)_2_	H	**h**	1	**2h** (99)
9	4-Cl	H	**i**	24	**2i** (56) ^c^
10	4-CF_3_	OMe	**j**	24	**2j** (10) ^d^

^a^ Reactions were carried out on 0.35 mmol scale of **1** in 2 mL of CH_2_Cl_2_; ^b^ The starting alkyne **1f** was recovered in 8% yield; ^c^ The starting alkyne **1i** was recovered in 18% yield; ^d^ The starting alkyne **1j** was recovered in 60% yield.

**Table 3:**
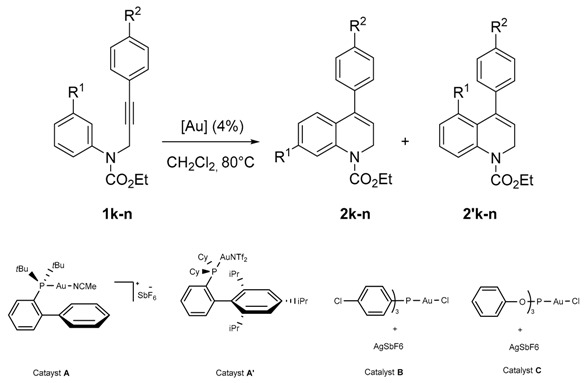
*Ortho*- vs. *para*-position annulation in the gold(I)-catalyzed IMHA of the *N*-ethoxycarbonyl-*N*-propargylanilines **1k**–**n**^a^.

Entry	R^1^	R^2^	Catalyst	Overall Yield (%) ^b^	2/2′	(Ratio) ^c^
1	OMe	H	A	99	**2k/2′k**	(67/33)
2			A′	99		(94/6)
3			B	90		(44/56)
4			C	67		(46/54)
5	OMe	COMe	A	99	**2l/2′l**	(61/39)
6			A′	99		(75/25)
7			B	99		(54/46)
8			C	86		(54/46)
9	OMe	OMe	A	70	**2m/2′m**	(91/9)
10			A′	73		(91/9)
11			B	83		(63/37)
12			C	77		(51/49)
13	COMe	COOMe	A	85	**2n/2′n**	(88/12)
14			A′	75		(64/36)
15			B	99		(40/60)
16			C	72		(19/81)
17	COMe	COMe	A	88	**2o/2′o**	(88/12)
18			A′	90		(65/35)
19			B	70		(33/67)
20			C	82		(20/80)

^a^ Reactions were carried out on 0.35 mmol of **1k**–**n** in 2 mL in CH_2_Cl_2_; ^b^ Overall yield refers to the mixture of regioisomers 2 + 2′; ^c^ The isomeric ratio was determined by ^1^H NMR analyses.

## Data Availability

Data is contained within the article or supplementary material.

## Supplementary Materials

The following are available online, general information of reagents and methods, synthetic procedures, and characterization data.

## Sample Availability

Samples of the compounds are not available from the authors.
