# Supplementary material for: Synthesis of 4-Substituted-1,2-Dihydroquinolines by Means of Gold-Catalyzed Intramolecular Hydroarylation Reaction of N-Ethoxycarbonyl-N-Propargylanilines
Source: Molecules. 2021 Jun 2;26(11):3366. doi: 10.3390/molecules26113366 (PMC8199670; doi:10.3390/molecules26113366)
Supplement: Supplementary file 1 [file molecules-26-03366-s001.zip › molecules-1232963-supplementary.pdf]

Supplementary materials for

Synthesis of 4-Substituted-1,2-Dihydroquinolines by means of Gold-Catalyzed Intramolecular Hydroarylation Reaction of *N*-Ethoxycarbonyl-*N*-Propargylanilines

Antonio Arcadi [1], Andrea Calcaterra [2], Giancarlo Fabrizi [2], Andrea Fochetti [2], Antonella Goggiamani [2,*], Antonia Iazzetti [2,*], Federico Marrone [2,] Vincenzo Marsicano [1], Giulia Mazzoccanti[2], and Andrea Serraiocco [2]

[1] Dipartimento di Scienze Fisiche e Chimiche, Università degli Studi di L'Aquila, Via Vetoio, 67100 Coppito (AQ), Italy; antonio.arcadi@univaq.it (A.A.);

[2] Dipartimento di Chimica e Tecnologie del Farmaco, Dipartimento di Eccellenza 2018- 2022, Sapienza Università di Roma, P.le A. Moro 5, 00185 Rome, Italy; andrea.fochetti@uniroma1.it (A.F.), andrea.calcaterra@uniroma1.it (A. C), giancarlo.fabrizi@uniroma1.it (G.F.), federico.marrone@uniroma1.it (F.M), vincenzo.marsicano@graduate.univaq.it (V. M), giulia.mazzoccanti@uniroma1.it (G.M); andrea.serraiocco@uniroma1.it (A.S.);

* Correspondence: antonia.iazzetti@uniroma1.it (A.I.); antonella.goggiamani@uniroma1.it (A.G.)

Table of Contents

1. GENERAL INFORMATION 2

1.1. Reagents and methods 2

2. SYNTHETIC PROCEDURES 2

2.1 Typical procedure for the preparation of the ethyl phenylcarbamate: 2

2.2 Typical procedure for the preparation of the ethyl phenyl(prop-2-yn-1-yl)carbamate: 3

2.3 Typical procedure for the preparation of the ethyl phenyl(3-phenylprop-2-yn-1-yl)carbamate **1a**: 3

2.4 Typical procedure for the preparation of the ethyl 4-phenylquinoline-1(2*H*)-carboxylate **2a**: 4

2.5 Typical procedure for the preparation of **2k/2'k:** 4

3. CHARACTERIZATION DATA 6

3.1 Characterization data of compounds **1b – 1o** 6

3.2 Characterization data of compounds **2b – o**, **2'l – o**, **3a**, **4a**, **5b**, **6b**, **7b** 12

^{1}H, ^{13}C ^{19}F NMR SPECTRA OF COMPOUNDS **2a – j**, **2k – o**, **2'k – o**, **3a**, **5b**, **6b**, **7b** 21

HRMS SPECTRA OF COMPOUNDS **2a – j**, **2k – o**, **2'k – o**, **3a**, **5b**, **6b**, **7b** 45

1. GENERAL INFORMATION

1.1. Reagents and methods

All the commercially available reagents, catalysts, bases and solvents were used as purchased, without further purification. Reaction products **2a** -**e** and **2g** - **h** were filtered on a pad of SiO_2 using AcOEt, while reaction products **2f**, **2i** and **2j** were purified by chromatography on SiO_2 (25-40 µm), eluting with *n*-hexane/AcOEt mixtures. Reaction products **2k/2'k – 2o/2'o** were obtained as isomeric mixtures by filtration on a pad of SiO_2 using AcOEt to eliminate the catalysts before calculating the isomeric ratio by ^1H NMR. When possible, to obtain suitable NMR spectra of each compound, the isomeric mixtures were further purified by semi-preparative HPLC under normal phase condition using a Nucleodur 100-5 column (762007.100) and eluting with *n*-hexane/AcOEt mixtures. ^1H NMR (400.13 MHz), ^{13}C NMR (100.6 MHz), and ^{19}F spectra (376.5 MHz) were recorded with a Bruker Avance 400 spectrometer. Splitting patterns are designed as s (singlet), d (doublet), t (triplet), q (quartet), m (multiplet), or bs (broad singlet). IR spectra were recorded with a Jasco FT/IR-430 spectrometer. HRMS were recorded with an Orbitrap Exactive Mass spectrometer with ESI source. Melting points were determined with a Büchi B-545 apparatus and are uncorrected.

2. SYNTHETIC PROCEDURES

Starting materials **1** have been synthetized according to the following scheme 1:

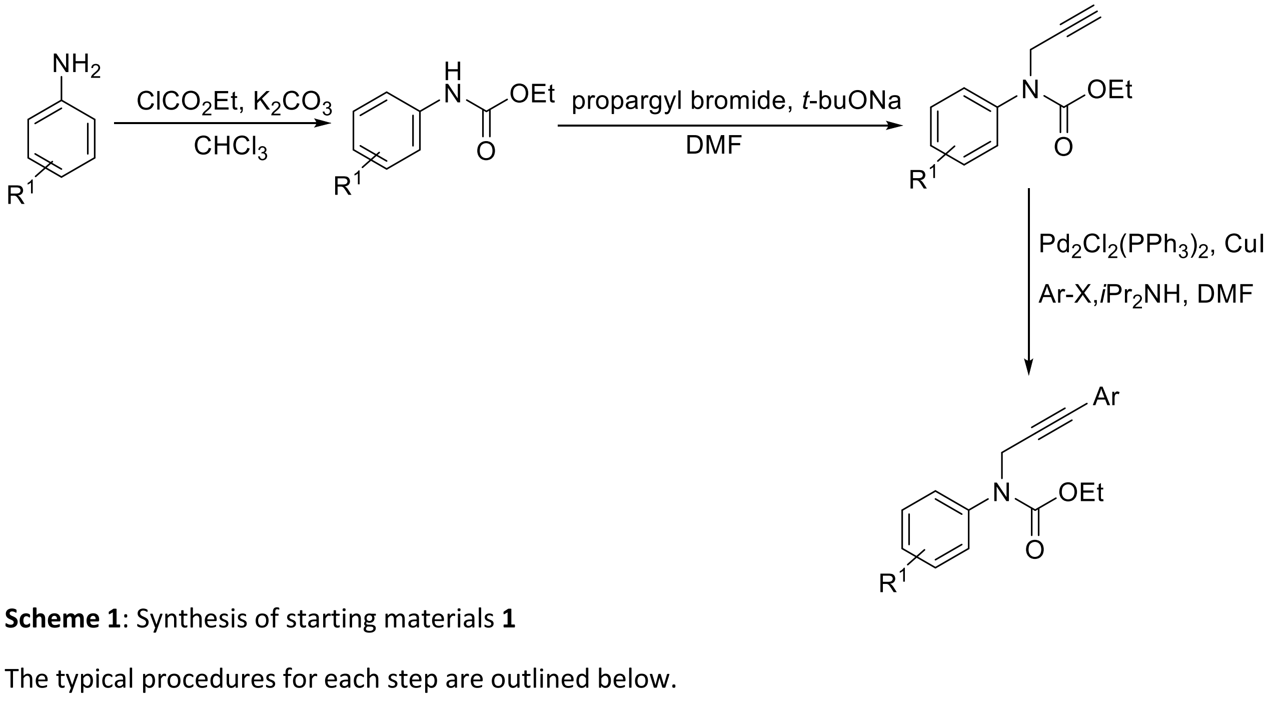

Scheme 1: Synthesis of starting materials **1**

The typical procedures for each step are outlined below.

2.1 Typical procedure for the preparation of the ethyl phenylcarbamate:

A solution of aniline (1.02 g, 1 ml, 11 mmol, 1 equiv.) in chloroform (15 ml) was cooled to 0°C in a flask equipped with a magnetic stirring bar, then ethyl chloroformate (1.43 g, 1.26 ml, 13.2 mmol, 1.2 equiv.) and K_2CO_3 (3.03 g, 22 mmol, 2 equiv.) were added while stirring. The reaction was slowly allowed to warm to room temperature and monitored by TLC until complete consumption of the aniline. After this time, the reaction mixture was cooled again to 0°C and quenched with 20 ml of water, before being diluted with CH_2Cl_2 and washed with 2N solution of HCl and brine. The organic layer was dried over Na_2SO_4, filtered, and concentrated under reduced pressure. The crude product as a pale-yellow solid (1,34 g, 74% yield) was used without further purification.

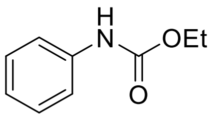

Yield: 74% (1343.9 mg);
ethyl phenylcarbamate: known compound; [1] lit. mp = 49 – 50 °C; [1] pale yellow solid; mp = 51 – 52 °C; IR (neat): 3310, 2978, 1703, 1599, 1314, 1065 cm^{-1}; ^{1}H NMR (400.13 MHz) ($CDCl_3$): δ = 7.40 (d, *J* = 8.1 Hz, 2 H), 7.33 (t, *J* = 8.1 Hz, 2 H), 7.08 (t, *J* = 8.1 Hz, 1 H), 6.62 (bs, 1 H), 4.25 (q, *J* = 7.1 Hz, 2 H), 1.34 (t, *J* = 7.1 Hz, 3 H); ^{13}C NMR (100.6 MHz) ($CDCl_3$): δ = 153.8, 138.2, 129.2, 123.6, 118.8, 61.4, 14.8.

2.2 Typical procedure for the preparation of the ethyl phenyl(prop-2-yn-1-yl)carbamate:

A flask with a magnetic stirring bar was charged with ethyl phenylcarbamate (1.3g, 7.9 mmol, 1 equiv.) dissolved in *N,N*-dimethylformamide (10 ml) and cooled to 0°C. Sodium *tert*-butoxide (1.14 g, 11.85 mmol, 1.5 equiv.) was added and the resulting solution was stirred for 10 minutes before adding propargyl bromide (1.13 g, 817 µl, 9.48 mmol, 1.2 equiv.). The reaction was allowed to warm to room temperature and after 17 hours was again cooled to 0°C and quenched with 20 ml of water, before being diluted with Et_2O and washed with brine. The organic layer was dried over Na_2SO_4, filtered and concentrated under reduced pressure. The residue was purified by chromatography on SiO_2 (25-40 µm), eluting with a 95/5 (v/v) *n*-hexane/AcOEt mixture *(R_f* = 0.24) to obtain 1.24 g (77% yield) of ethyl phenyl(prop-2-yn-1-yl)carbamate.

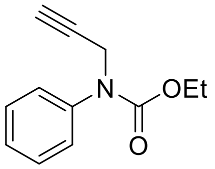

Yield: 77% (1241.5 mg); *n*-hexane/AcOEt mixture 95/5 (v/v) *(R_f* = 0.22)
ethyl phenyl(prop-2-yn-1-yl)carbamate: known compound; [2] orange oil; IR (neat): 2917, 2304, 1716, 1603, 1443, 1313 cm^{-1}; ^{1}H NMR (400.13 MHz) ($CDCl_3$): δ = 7.42 – 7.27 (m, 5 H), 4.44 (d, *J* = 2.6 Hz, 2 H), 4.22 (q, *J* = 6.8 Hz, 2 H), 2.29 (t, *J* = 2.6 Hz, 1 H), 1.26 (t, *J* = 6.8 Hz, 3 H); ^{13}C NMR (100.6 MHz) ($CDCl_3$): δ = 155.2, 129.0, 128.9, 126.8, 126.6, 79.6, 72.1, 62.1, 40.1, 14.5.

2.3 Typical procedure for the preparation of the ethyl phenyl(3-phenylprop-2-yn-1-yl)carbamate 1a:

A flask equipped with a magnetic stirring bar was charged with $PdCl_2(PPh_3)_2$ (79 mg, 0.11 mmol, 0.02 equiv.) and CuI (43 mg, 0.23 mmol, 0.04 equiv.) dissolved in diisopropylamine (12 mL) and *N,N*-dimethylformamide (6 mL). The resultant solution was stirred under nitrogen at room temperature for 10 minutes before adding iodobenzene (1385 mg, 760 µl, 6.8 mmol, 1.2 equiv.) and ethyl phenyl(prop-2-yn-1-yl)carbamate (1151 mg, 5.66 mmol, 1 equiv.) and stirred for 2 hours at room temperature. After this time, the reaction mixture was diluted with Et_2O and washed with a saturated NH_4Cl solution and with brine. The organic layer was dried over Na_2SO_4, filtered, and concentrated under reduced pressure. The residue was purified by chromatography on SiO_2 (25-40 µm), eluting with a 95/5 (v/v) *n*-hexane/AcOEt mixture *(R_f* = 0.24) to obtain 1439 mg (91% yield) of ethyl phenyl(3-phenylprop-2-yn-1-yl)carbamate **1a**.

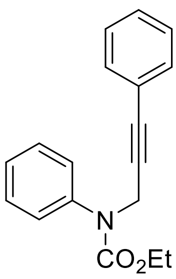

1a

Yield: 91% (1439.0 mg); *n*-hexane/AcOEt mixture 95/5 (v/v) (R_f = 0.24)

1a: orange oil; IR (neat): 2980, 2243, 1705, 1596, 1494 cm^{-1}; ^{1}H NMR (400.13 MHz) ($CDCl_3$): δ = 7.61 - 7.17 (m, 10 H), 4.67 (s, 2 H), 4.24 (bq, *J*= 7.2 Hz, 2 H), 1.34-1.28 (m, 3 H); ^{13}C NMR (100.6 MHz) ($CDCl_3$): δ = 155.2 (q), 141.7 (q), 131.7 (CH), 128.9 (CH), 128.3 (CH), 128.2 (CH), 126.8 (CH), 122.8 (q), 85.1 (q), 84.0 (q), 62.1 (CH_2), 40.8 (CH_2), 14.6 (CH_3); MS (EI ion source): *m/z* (%) = 280 (22 [M^+]), 236 (4), 134 (35), 115 (100), 63 (36); HRMS: *m/z* $[M + H]^+$ calcd for $C_{18}H_{18}NO_2$: 280.1332; found: 280.1333.

2.4 Typical procedure for the preparation of the ethyl 4-phenylquinoline-1(2*H*)-carboxylate 2a:

A Carousel Tube Reactor (Radely Discovery Technology) equipped with a magnetic stirring bar was charged with ethyl phenyl(3-phenylprop-2-yn-1-yl)carbamate (97.8 mg, 0.35 mmol, 1 equiv.) **1a**, CH_2Cl_2 (2 mL), JohnPhosAu(MeCN)SbF_6 (10.8 mg, 0.014 mmol, 0.04 equiv) and sealed. Then the reaction mixture was stirred at 80°C and monitored by TLC until the disappearance of the starting material. After 1 hour the obtained mixture was cooled at room temperature and concentrated under reduced pressure. The residue was filtered on a pad of SiO_2 using AcOEt to afford 97.1 mg of ethyl 4-phenylquinoline-1(2*H*)-carboxylate **2a** (99% yield).

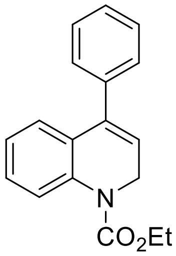

2a

Yield: 99% (97.1 mg);

2a: yellow oil ; IR (neat): 2912, 1707, 1380 cm^{-1}; ^{1}H NMR (400.13 MHz) ($CDCl_3$): δ = 7.69 (d, *J* = 7.4 Hz, 1 H), 7.45 – 7.36 (m, 5 H), 7.31 – 7.27 (m, 1 H), 7.12 – 7.05 (m, 2 H), 6.07 (t, *J* = 4.5 Hz, 1 H), 4.51 (d, *J* = 4.5 Hz, 2 H), 4.33 (q, *J* = 7.1 Hz, 2 H), 1.39 (t, *J* = 7.1 Hz, 3 H); ^{13}C NMR (100.6 MHz) ($CDCl_3$): δ = 154.2 (q), 138.9 (q), 137.3 (q), 129.3 (q), 128.8 (CH), 128.5 (CH), 127.9 (CH), 127.7 (CH), 126.1 (CH), 124.2 (CH), 124.1 (CH), 123.4 (CH), 62.3 (CH_2), 43.1 (CH_2), 14.7 (CH_3); MS (EI ion source): *m/z* (%) = 279 (18, [M^+]), 206 (100), 204 (62), 102 (21); HRMS: *m/z* $[M + Na]^+$ calcd for $C_{18}H_{17}NO_2Na$: 302.1152; found: 302.1152.

2.5 Typical procedure for the preparation of 2k/2'k:

A Carousel Tube Reactor (Radely Discovery Technology) equipped with a magnetic stirring bar was charged with ethyl (3-methoxyphenyl)(3-phenylprop-2-yn-1-yl)carbamate **1k** (108.2 mg, 0.35 mmol, 1 equiv) and CH_2Cl_2 (2 mL) before adding catalyst A, A' or catalyst C (catalyst A: JohnPhosAu(MeCN)SbF_6 10.8 mg, 0.014 mmol, 0.04 equiv; A': XphosAuCl 9.9 mg, 0.014 mmol, 0.04 equiv. and $AgNTf_2$ 5.4 mg, 0.014, 0.04 equiv.; catalyst B: (*p*-ClC_6H_4)PAuCl 8.4 mg, 0.014 mmol, 0.04 equiv. and $AgSbF_6$ 4.8 mg, 0.014 mmol, 0.04 equiv;

catalyst C: $(C_6H_5O)_3PAuCl$ 7.6 mg, 0.014 mmol, 0.04 equiv. and $AgSbF_6$ 4.8 mg, 0.014 mmol, 0.04 equiv).The resulting mixture was stirred at 80 °C for one hour, then it was cooled, concentrated under reduced pressure and the residue was filtered on a pad of SiO_2 to afford 107.2 mg of **2k** + **2'k** (99% overall yield) using catalyst A, or 107.1 mg of **2k** + **2'k** (99% overall yield) using catalyst A', or 97.1 mg of **2k** +**2'k** (90% overall yield) using catalyst B, or 72.7 mg of **2k** + **2'k** (67% overall yield) using catalyst C. **2k/2'k** ratio was calculated by ^{1}H NMR analyses. Afterwards, the two isomers were separated by semi-preparative HPLC to obtain suitable NMR spectra of each compound using a 85/5 (v/v) *n*-hexane/AcOEt mixture *(R_f* = 0.22) as eluent.

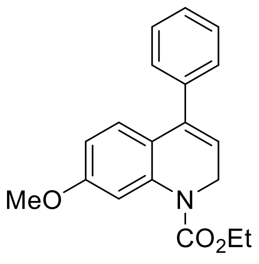

2k

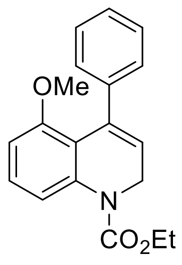

2'k

HPLC eluent = *n*-hexane/AcOEt mixture 85/15 (v/v) *(R_f* = 0.22)
Overall yield (catalyst A): 99% (107.2 mg); **2k/2'k =** 67/33
Overall yield (catalyst A'): 99% (107.1 mg); **2k/2'k =** 94/6
Overall yield (catalyst B): 90% (97.1 mg); **2k/2'k =** 44/56
Overall yield (catalyst C): 67% (72.7 mg); **2k/2'k =** 46/54
2k: orange oil; IR (neat): 2980, 2243, 1705, 1596, 1494 cm^{-1}; ^{1}H NMR (400.13 MHz) ($CDCl_3$): δ = 7.29 – 7.21 (m, 6 H), 6.90 (d, *J* = 8.7 Hz, 1 H), 6.52 (dd, J_1 = 8.7 Hz, J_2 = 2.6 Hz, 1 H), 5.80 (t, *J* = 4.5 Hz, 1 H), 4.38 (d, *J* = 4.6 Hz, 2 H), 4.21 (q, *J* = 7.1 Hz, 2 H), 3.74 (s, 3 H), 1.27 (t, *J* = 7.1 Hz, 3 H); ^{13}C NMR (100.6 MHz) ($CDCl_3$): δ = 159.1 (q), 154.1 (q), 139.2 (q), 138.7 (q), 138.6 (q), 128.8 (CH), 128.5 (CH), 127.8 (CH), 127.0 (CH), 122.5 (q), 120.4 (CH), 110.2 (CH), 109.7 (CH), 62.3 (CH_2), 55.5 (CH_3), 43.3 (CH_2), 14.7 (CH_3). MS (EI ion source): *m/z* (%) = 309 (0.2, [M^+]), 235 (100), 220 (17), 204 (29), 191 (24), 165 (15); HRMS: *m/z* $[M + Na]^+$ calcd for $C_{19}H_{19}NO_3Na$: 332.1257; found: 332.1260.

2'k: colorless oil; IR (neat): 2982, 1708, 1610, 1504, 1466 cm^{-1}; ^{1}H NMR (400.13 MHz) ($CDCl_3$): δ = 7.36 - 7.24 (m, 6 H), 6.69 (d, *J* = 8.9 Hz, 1 H), 6.08 (t, *J* = 5.1 Hz, 1 H), 4.33 (d, *J* = 5.1 Hz, 2 H), 4.29 (q, *J* = 7.2 Hz, 2 H), 3.43 (s, 3 H), 1.35 (t, *J* = 7.2 Hz, 3 H); ^{13}C NMR (100.6 MHz) ($CDCl_3$): δ = 156.2 (q), 153.9 (q), 141.5 (q), 139.8 (q), 137.8 (q), 128.3 (CH), 127.7 (CH), 126.68 (CH), 126.62 (CH), 124.5 (CH), 118.6 (q), 117.0 (CH), 108.4 (CH), 62.2 (CH_2), 55.5 (CH_3), 42.7 (CH_2), 14.7 (CH_3); MS (EI ion source): *m/z* (%) = 309 (42, [M^+]), 280 (51), 236 (100), 220 (51), 193 (15); HRMS: *m/z* $[M + Na]^+$ calcd for $C_{19}H_{19}NO_3Na$: 332.1257; found: 332.1260.

3. CHARACTERIZATION DATA

3.1 Characterization data of compounds 1b – 1o

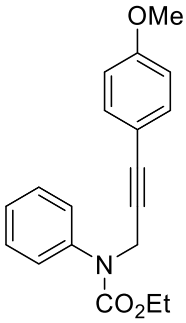

1b

Yield: 73%; *n*-hexane/AcOEt mixture 85/15 (v/v) (R_f = 0.24)

1b: red oil; IR (neat): 2978, 2239, 1704, 1604, 1511 cm^{-1}; ^1H NMR (400.13 MHz) ($CDCl_3$): δ = 7.41 – 7.40 (m, 4 H), 7.33 (d, J = 8.8 Hz, 2 H), 7.32 – 7.28 (m, 1 H), 6.84 (d, J = 8.8 Hz, 2 H), 4.65 (s, 2 H), 4.24 (q, J = 6.7 Hz, 2 H), 3.82 (s, 3 H), 1.27 (bt, J = 6.7 Hz, 3 H); ^{13}C NMR (100.6 MHz) ($CDCl_3$): δ = 159.7 (q), 155.4 (q), 133.2 (CH), 129.0 (CH), 126.9 (CH), 115.1 (q) 114.0 (CH), 84.0 (q), 83.7 (q), 62.1 (CH_2), 55.4 (CH_3), 41.0 (CH_2), 14.7 (CH_3); MS (EI ion source): *m/z* (%) = 309 (1, [M^+]), 280 (42), 236 (12), 145 (100), 102 (35), 77 (33); HRMS: *m/z* $[M + Na]^+$ calcd for $C_{19}H_{19}NO_3Na$: 332.1257; found: 332.1260.

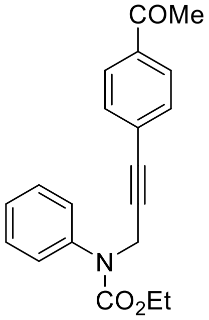

1c

Yield: 64%; *n*-hexane/AcOEt mixture 70/30 (v/v) (R_f = 0.23)

1c: red oil; IR (neat): 2980, 2241, 1684, 1599, 1496 cm^{-1}; ^1H NMR (400.13 MHz) ($CDCl_3$): δ = 7.90 (d, J = 8.4 Hz, 2 H), 7.48 - 7.28 (m, 7 H), 4.69 (s, 2 H), 4.24 (bq, J = 7.0 Hz, 2 H), 2.61 (s, 3 H), 1.27 (bt, J = 7.0 Hz, 3 H); ^{13}C NMR (100.6 MHz) ($CDCl_3$): δ = 197.3 (q), 155.2 (q), 141.5 (q), 136.3 (q), 131.8 (CH), 129.0 (CH), 128.2 (CH), 127.7 (q), 126.9 (CH), 126.8 (CH), 88.6 (q), 83.3 (q), 62.2 (CH_2), 40.9 (CH_2), 26.6 (CH_3), 14.6 (CH_3); MS (EI ion source): *m/z* (%) = 321 (14, [M^+]), 292 (81), 248 (19), 157 (50), 104 (100), 77 (48); HRMS: *m/z* $[M + Na]^+$ calcd for $C_{20}H_{19}NO_3Na$: 344.1257; found: 344.1248.

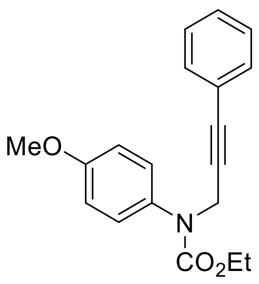

1d

Yield: 82%; *n*-hexane/AcOEt mixture 80/20 (v/v) *(R_f = 0.25)*

1d: red oil; IR (neat): 2979, 2241, 1705, 1608, 1511, 1248 cm^{-1}; ^{1}H NMR (400.13 MHz) ($CDCl_3$): δ = 7.41 – 7.39 (m, 2 H), 7.2 – 7.28 (m, 5 H), 6.93 (d, *J* = 8.9 Hz, 2 H), 4.62 (s, 2 H), 4.22 (m, 2 H), 3.84 (s, 3 H), 1.26 (m, 3 H); ^{13}C NMR (100.6 MHz) ($CDCl_3$): δ = 158.3 (q), 155.6 (q), 131.7 (CH), 128.25 (CH), 128.23, 122.9 (q), 114.1 (CH), 85.2 (q), 84.0 (q), 62.0 (CH_2), 55.4 (CH_3), 41.1 (CH_2), 14.6 (CH_3); MS (EI ion source): *m/z* (%) = 309 (11, [M$^+$]), 280 (34), 236 (12), 134 (39), 115 (100), 63 (34); HRMS: *m/z* [M + Na]$^+$ calcd for $C_{19}H_{19}NO_3Na$: 332.1257; found: 332.1261.

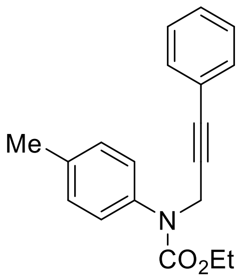

1e

Yield: 92%; *n*-hexane/AcOEt mixture 90/10 (v/v) *(R_f = 0.24)*

1e: orange oil; IR (neat): 2980, 2240, 1705, 1598, 1489 cm^{-1}; ^{1}H NMR (400.13 MHz) ($CDCl_3$): δ = 7.42 – 7.39 (m, 2 H), 7.32 – 7.27 (m, 5 H), 7.22 – 7.20 (m, 2 H), 4.64 (s, 2 H), 4.23 (bq, *J* = 8.9 Hz, 2 H), 2.39 (s, 3 H), 1.27 (bs, 3 H); ^{13}C NMR (100.6 MHz) ($CDCl_3$): δ = 155.5 (q), 139.1 (q), 136.8 (q), 131.8 (CH), 129.7 (CH), 128.3 (CH), 126.9 (CH), 123.0 (q), 85.3 (q), 84.0 (q), 62.1 (CH_2), 41.1 (CH_2), 21.2 (CH_3), 14.7 (CH_3). MS (EI ion source): *m/z* (%) = 293 (11, [M$^+$]), 265 (5) 220 (17) 118 (44), 115 (100), 91 (35), 63 (37); HRMS: *m/z* [M + H]$^+$ calcd for $C_{19}H_{20}NO_2$: 294.1489; found: 294.1491.

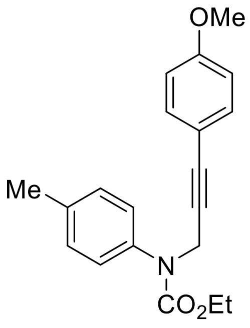

1f

Yield: 91%; *n*-hexane/AcOEt mixture 75/25 (v/v) *(R_f = 0.28)*

1f: red oil; IR (neat): 2978, 2241, 1703, 1606, 1509 cm^{-1}; ^1H NMR (400.13 MHz) ($CDCl_3$): δ = 7.34 (d, *J* = 8.8 Hz, 2 H), 7.27 (d, *J* = 8.2 Hz, 2 H), 7.20 (d, *J* = 8.2 Hz, 2 H), 6.84 (d, *J* = 8.8 Hz, 2 H), 4.62 (s, 2 H), 4.24 (bq, *J* = 6.7 Hz, 2 H), 3.82 (s, 3 H), 2.38 (s, 3 H), 1.26 (bs, 3 H); ^{13}C NMR (100.6 MHz) ($CDCl_3$): δ = 159.7 (q), 155.5 (q), 136.7 (q), 133.2 (CH), 129.6 (CH), 126.9 (CH), 115.2 (q), 114.0 (CH), 83.9 (q), 83.8 (q), 62.1 (CH_2), 55.4 (CH_3), 41.1 (CH_2), 21.2 (CH_3), 14.7 (CH_3); MS (EI ion source): *m/z* (%) = 323 (1, [M^+]), 294 (24), 250 (6), 145 (100), 135 (21), 102 (33); HRMS: *m/z* $[M + Na]^+$ calcd for $C_{20}H_{21}NO_3Na$: 346.1414; found: 346.1414.

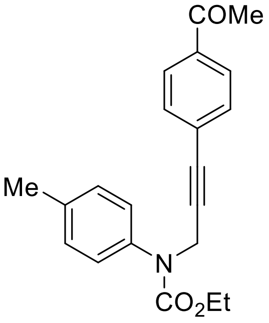

1g

Yield: 63%; *n*-hexane/AcOEt mixture 75/25 (v/v) *(R_f = 0.24)*
1g: brown oil; IR (neat): 3034, 2980, 2250, 1706, 1601, 1514 cm^{-1}; ^1H NMR (400.13 MHz) ($CDCl_3$): δ = 7.90 (d, *J* = 8.4 Hz, 2 H), 7.47 (d, *J* = 8.0 Hz, 2 H), 7.25 - 7.19 (m, 4 H), 4.66 (s, 2 H), 4.22 (bq, *J* = 6.8 Hz, 2 H), 2.61 (s, 3 H), 2.39 (s, 3 H), 1.26 (bs, 3 H); ^{13}C NMR (100.6 MHz) ($CDCl_3$): δ = 197.3 (q), 155.4 (q), 136.8 (q), 136.3 (q), 131.8 (CH), 129.7 (CH), 129.6 (q), 128.1 (CH), 127.8 (CH), 126.7 (q), 88.7 (q), 83.3 (q), 62.1 (CH_2), 41.0 (CH_2), 26.6 (CH_3), 21.1 (CH_3), 14.6 (CH_3); MS (EI ion source): *m/z* (%) = 335 (31, [M^+]), 306 (72), 262 (21), 157 (71), 118 (100), 114 (75); HRMS: *m/z* $[M + H]^+$ calcd for $C_{21}H_{22}NO_3$: 336.1594; found: 336.1597.

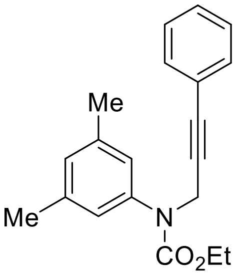

1h

Yield: 64%; *n*-hexane/AcOEt mixture 90/10 (v/v) *(R_f = 0.26)*
1h: yellow oil; IR (neat): 2979, 2239, 1699, 1598, 1378, 1308, 1240 cm^{-1}; ^1H NMR (400.13 MHz) ($CDCl_3$): δ = 7.32 - 7.29 (m, 2 H), 7.22 - 7.18 (m, 3 H), 6.91 (s, 2 H), 6.84 (s, 1 H), 4.52 (s, 2 H), 4.14 (q, *J* = 6.8 Hz, 2 H), 2.25 (s, 6 H), 1.18 (t, *J* = 6.8 Hz, 3 H); ^{13}C NMR (100.6 MHz) ($CDCl_3$): δ = 155.3 (q), 141.5 (q), 138.5 (q), 131.6 (CH), 128.6 (CH), 128.25 (CH), 128.21 (CH), 124.6 (CH), 123.0 (q), 85.3 (q), 83.9 (q), 62.0 (CH_2), 41.0 (CH_2), 21.3 (CH_3), 14.6 (CH_3); MS (EI ion source): *m/z* (%) = 307 (78, [M^+]), 278 (54), 193 (26), 132 (52), 115 (100), 105 (46); HRMS: *m/z* $[M + Na]^+$ calcd for $C_{20}H_{21}NO_2Na$: 330.1465; found: 330.1469.

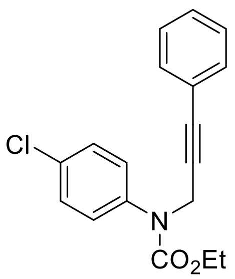

1i

Yield: 52%; *n*-hexane/AcOEt mixture 85/15 (v/v) *(R_f = 0.25)*

1i: yellow oil; IR (neat): 2981, 2240, 1710, 1595, 1493, 1378, 1285 cm^{-1}; ^1H NMR (400.13 MHz) ($CDCl_3$): δ = 7.31 - 7.18 (m, 9 H), 4.54 (s, 2 H), 4.14 (q, *J* = 6.8 Hz, 2 H), 1.18 (t, *J* = 6.8 Hz, 3 H); ^{13}C NMR (100.6 MHz) ($CDCl_3$): δ = 155.0 (q), 132.4 (q), 131.7 (CH), 129.1 (CH), 128.4 (CH), 128.3 (CH), 128.1 (CH), 122.6 (q), 84.6 (q), 84.4 (q), 62.3 (CH_2), 40.7 (CH_2), 14.6 (CH_3); MS (EI ion source): *m/z* (%) = 313 (5, [M^+]), 284 (71), 240 (14), 138 (32), 115 (100), 89 (11); HRMS: *m/z* [M + Na]$^+$ calcd for $C_{18}H_{16}ClNO_2Na$: 336.0762; found: 336.0769.

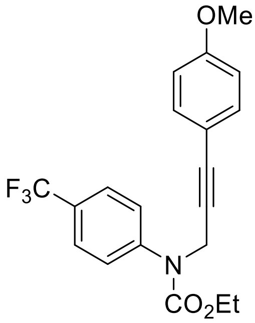

1j

Yield: 86%; *n*-hexane/AcOEt mixture 70/30 (v/v) *(R_f = 0.26)*

1j: red oil; IR (KBr): 2981, 2243, 1714, 1608, 1509 cm^{-1}; ^1H NMR (400.13 MHz) ($CDCl_3$): δ = 7.66 (d, *J* = 8.8 Hz, 2 H), 7.56 (d, *J* = 8.0 Hz, 2 H), 7.33 (d, *J* = 8.8 Hz, 2 H), 6.84 (d, *J* = 8.8 Hz, 2 H), 4.68 (s, 2 H), 4.27 (q, *J* = 7.2 Hz, 2 H), 3.82 (s, 3 H), 1.31 (t, *J* = 7.2 Hz, 3 H); ^{13}C NMR (100.6 MHz) ($CDCl_3$): δ = 160.0 (q), 154.9 (q), 145.1 (q), 133.3 (CH), 128.5 (q, q, *J* = 32.3 Hz), 126.5 (CH), 126.1 (CH, q, *J* = 3.7 Hz), 124.2 (q, q, *J* = 270.2 Hz), 114.8 (q), 114.1 (CH), 84.6 (q), 83.2 (q), 62.7 (CH_2), 55.5 (CH_3), 40.7 (CH_2), 14.7 (CH_3); ^{19}F NMR (376.5 MHz) ($CDCl_3$) δ -62.3; MS (EI ion source): *m/z* (%) = 358 (25), 281 (30), 207 (97), 73 (100); HRMS: *m/z* [M + Na]$^+$ calcd for $C_{20}H_{18}F_3NO_3Na$: 400.1131; found: 400.1121.

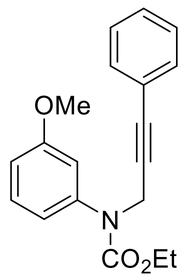

1k

Yield: 95%; *n*-hexane/AcOEt mixture 75/25 (v/v) *(R_f = 0.24)*

1k: brown oil; IR (neat): 2979, 2241, 1696, 1605, 1489 cm^{-1}; ^1H NMR (400.13 MHz) ($CDCl_3$): δ = 7.42 – 7.39 (m, 2 H), 7.33 – 7.28 (m, 4 H), 7.01 (m, 2 H), 6.86 (m, 1 H), 4.65 (s, 2 H), 4.25 (q, *J* = 7.1 Hz, 2 H), 3.83 (s, 3 H), 1.29 (t, *J* = 7.1 Hz, 3 H); ^{13}C NMR (100.6 MHz) ($CDCl_3$): δ = 159.9 (q), 155.1 (q), 142.8 (q), 131.7 (CH), 129.5 (CH),

128.3 (CH), 128.2 (CH), 122.8 (q), 119.0 (CH), 112.5 (CH), 85.1 (q), 84.0 (q), 62.1 (CH_2), 55.3 (CH_3), 40.9 (CH_2), 14.6 (CH_3); MS (EI ion source): *m/z* (%) = 309 (8, [M^+]), 280 (22), 236 (4), 134 (35), 115 (100), 63 (36); HRMS: *m/z* $[M + Na]^+$ calcd for $C_{19}H_{19}NO_3Na$: 332.1257; found: 332.1260.

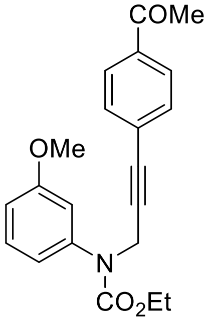

1l

Yield: 83%; *n*-hexane/AcOEt mixture 80/20 (v/v) (R_f = 0.22)

1l: red oil; IR (neat): 2979, 2246, 1707, 1602, 1491, 1264 cm^{-1}; ^1H NMR (400.13 MHz) ($CDCl_3$): δ = 7.90 (d, *J* = 8.5 Hz, 2 H), 7.48 (d, *J* = 8.5 Hz, 2 H), 7.31 (t, *J* = 8.0 Hz, 1 H), 6.99 – 6.96 (m, 2 H), 6.86 (dd, J_1 = 8.3 Hz, J_2 = 1.9 Hz, 1 H), 4.67 (s, 2 H), 4.24 (q, *J* = 7.0 Hz, 2 H), 3.82 (s, 3 H), 2.61 (s, 3 H), 1.28 (bt, *J* = 6.9 Hz, 3 H); ^{13}C NMR (100.6 MHz) ($CDCl_3$): δ = 197.4 (q), 160.1 (q), 155.2 (q), 142.8 (q), 136.4 (q), 131.9 (CH), 129.7 (CH), 128.3 (CH), 127.8 (q), 119.1 (CH), 112.9 (CH), 112.6 (CH), 88.7 (q), 83.4 (q), 62.3 (CH_2), 55.5 (CH_3), 41.0 (CH_2), 26.7 (CH_3), 14.7 (CH_3); MS (EI ion source): *m/z* (%) = 351 (31, [M^+]), 322 (50), 278 (14), 157 (46), 134 (100), 114 (62); HRMS: *m/z* $[M + Na]^+$ calcd for $C_{21}H_{21}NO_4Na$: 374.1363; found: 374.1354.

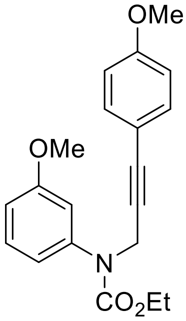

1m

Yield: 79%; *n*-hexane/AcOEt mixture 70/30 (v/v) (R_f = 0.23)

1m: red oil; IR (neat): 2977, 2241, 1702, 1605, 1509, 1243 cm^{-1}; ^1H NMR (400.13 MHz) ($CDCl_3$): δ = 7.34 (d, *J* = 8.8 Hz, 2 H), 7.30 (t, *J* = 8.8 Hz, 1 H), 7.01 – 6.99 (m, 2 H), 6.86 – 6.81 (m, 3 H), 4.63 (s, 2 H), 4.24 (q, *J* = 7.0 Hz, 2 H), 3.82 (s, 6 H), 1.28 (t, *J* = 7.0 Hz, 3 H); ^{13}C NMR (100.6 MHz) ($CDCl_3$): δ = 160.0 (q), 159.7 (q), 155.3 (q), 143.0 (q), 133.2 (CH), 129.6 (CH), 119.2 (q), 115.1 (CH), 114.0 (CH), 112.8 (CH), 112.6 (CH), 84.0 (q), 83.8 (q), 62.2 (CH_2), 55.5 (CH_3), 55.4 (CH_3), 41.0 (CH_2), 14.7 (CH_3); MS (EI ion source): *m/z* (%) = 310 (32), 266 (8), 207 (19), 145 (100), 135 (50), 73 (55); HRMS: *m/z* $[M + Na]^+$ calcd for $C_{20}H_{21}NO_4Na$: 362.1363; found: 362.1357.

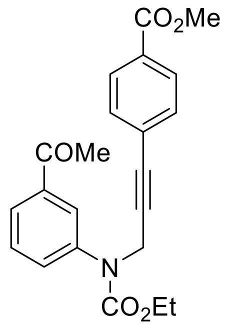

1n

Yield: 76%; *n*-hexane/AcOEt mixture 70/30 (v/v) *(R_f = 0.26)*

1n: red oil; IR (KBr): 2982, 2239, 1684, 1605, 1270, 1056 cm^{-1}; ^{1}H NMR (400.13 MHz) ($CDCl_3$): δ = 8.03 (bs, 1 H), 7.98 (d, *J* = 8.8 Hz, 2 H), 7.89 (d, *J* = 8 Hz, 1 H), 7.62 - 7.60 (m, 1 H), 7.51 (t, *J* = 8 Hz, 1 H) 7.45 (d, *J* = 8.8 Hz, 2 H), 4.72 (s, 2 H), 4.25 (q, *J* = 7.2 Hz, 2 H), 3.93 (s, 3 H), 2.62 (s, 3 H), 1.28 (bs, 3 H); ^{13}C NMR (100.6 MHz) ($CDCl_3$): δ = 197.2 (q), 166.4 (q), 154.9 (q), 142.0 (q), 138.0 (q), 131.6 (CH), 131.5 (CH), 129.8 (CH), 129.5 (CH), 129.2 (CH), 127.2 (q), 126.8 (CH), 126.4 (q), 87.7 (q), 83.8 (q), 62.4 (CH_2), 52.2 (CH_3), 40.6 (CH_2), 26.6 (CH_3), 14.5 (CH_3). MS (EI ion source): *m/z* (%) = 379 (54, [M^+]), 351 (25), 350 (100), 306 (35), 173 (35), 146 (23); HRMS: *m/z* [M + Na]$^+$ calcd for $C_{22}H_{21}NO_5Na$: 402.1312; found: 402.1311.

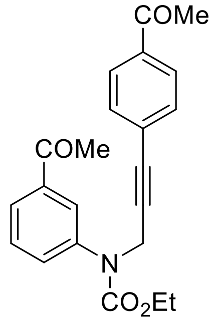

1o

Yield: 65%; *n*-hexane/AcOEt mixture 65/35 (v/v) *(R_f = 0.23)*

1o: orange oil; IR (neat): 2980, 2241, 1683, 1600, 1485, 1358, 1262 cm^{-1}; ^{1}H NMR (400.13 MHz) ($CDCl_3$): δ = 8.0 (s, 1 H), 7.91 - 7.88 (m, 3 H), 7.61 (d, *J* = 7.6 Hz, 1 H), 7.52 (t, *J* = 8.0 Hz, 1 H), 7.48 (d, *J* = 7.5 Hz, 2 H), 4.72 (s, 2 H), 4.25 (bq, *J* = 7.2 Hz, 2 H), 2.62 (s, 3 H), 2.60 (s, 3 H), 1.28 (bs, 3 H); ^{13}C NMR (100.6 MHz) ($CDCl_3$): δ = 197.3 (q), 154.9 (q), 141.9 (q), 138.0 (q), 136.4 (q), 131.8 (CH), 131.5 (CH), 129.2 (CH), 128.2 (CH), 127.4 (q), 126.8 (CH), 126.4 (CH), 88.0 (q), 83.8 (q), 62.5 (CH_2), 40.6 (CH_2), 26.7 (CH_3), 26.6 (CH_3), 14.5 (CH_3). MS (EI ion source): *m/z* (%) = 363 (56, [M^+]), 334 (100), 290 (35), 157 (20), 146(18); HRMS: *m/z* [M + Na]$^+$ calcd for $C_{22}H_{21}NO_4Na$: 386.1363; found: 386.1360.

3.2 Characterization data of compounds 2b – o, 2’l – o, 3a, 4a, 5b, 6b, 7b

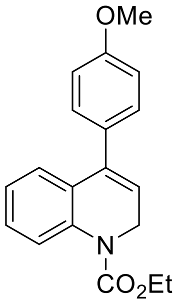

2b

Yield: 82% (88.9 mg);

2b; yellow oil; IR (neat): 2980, 1699, 1608, 1510, 1051 cm^{-1}; ^{1}H NMR (400.13 MHz) ($CDCl_3$): δ = 7.56 (bd, *J* = 7.7 Hz, 1 H), 7.19 (d, *J* = 8.8 Hz, 2 H), 7.18 – 7.15 (m, 1 H), 7.01 (dd, J_1 = 7.8 Hz, J_2 = 1.7 Hz, 1 H), 6.95 (dt, J_1 = 7.2 Hz, J_2 = 1.2 Hz, 1 H), 6.84 (d, *J* = 8.8 Hz, 2 H), 5.91 (t, *J* = 4.5 Hz, 1 H), 4.37 (d, *J* = 4.5 Hz, 2 H), 4.20 (q, *J* = 7.1 Hz, 2 H), 3.76 (s, 3 H), 1.26 (t, *J* = 7.1 Hz, 3 H); ^{13}C NMR (100.6 MHz) ($CDCl_3$): δ = 159.4 (q), 154.2 (q), 138.4 (q), 137.4 (q), 131.3 (q), 130.0 (CH), 129.5 (q), 127.6 (CH), 126.1 (CH), 124.15 (CH), 124.08 (CH), 122.5 (CH), 113.9 (CH), 62.2 (CH_2), 55.4 (CH_3), 43.1 (CH_2), 14.7 (CH_3); MS (EI ion source): *m/z* (%) = 309 (47, [M^+]), 280 (93), 236 (100), 221 (21), 192 (20); HRMS: *m/z* [M + Na]$^+$ calcd for $C_{19}H_{19}NO_3Na$: 332.1257; found: 332.1261.

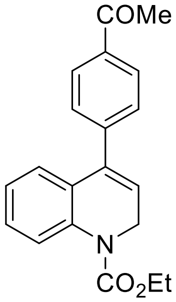

2c

Yield: 99% (111.3 mg);

2c: white solid; mp = 110 – 111 °C; IR (neat): 2977, 1698, 1604, 1484 cm^{-1}; ^{1}H NMR (400.13 MHz) ($CDCl_3$): δ = 7.98 (d, *J* = 8.5 Hz, 2 H), 7.67 – 7.65 (m, 1 H), 7.45 (d, *J* = 8.5 Hz, 2 H), 7.30 – 7.25 (m, 1 H), 7.06 – 6.99 (m, 2 H), 6.10 (t, *J* = 4.6 Hz, 1 H), 4.49 (d, *J* = 4.6 Hz, 2 H), 4.28 (q, *J* = 7.1 Hz, 2 H), 2.63 (s, 3 H), 1.34 (t, *J* = 7.1 Hz, 3 H); ^{13}C NMR (100.6 MHz) ($CDCl_3$): δ = 197.8 (q), 154.2 (q), 143.9 (q), 138.3 (q), 137.4 (q), 136.6 (q), 129.1 (CH), 128.7 (CH), 128.1 (CH), 126.0 (CH), 124.6 (CH), 124.4 (CH), 120.6 (q), 62.4 (CH_2), 43.1 (CH_2), 26.8 (CH_3), 14.7 (CH_3); MS (EI ion source): *m/z* (%) = 321 (27, [M^+]), 292 (71), 248 (100), 205 (28); HRMS: *m/z* [M + H]$^+$ calcd for $C_{20}H_{20}NO_3$: 322.1438; found: 322.1431.

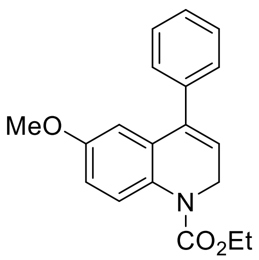

2d

Yield: 82% (88.6 mg);

2d: pale yellow oil; IR (neat): 2980, 1702, 1491, 1382 cm^{-1}; ^{1}H NMR (400.13 MHz) ($CDCl_3$): δ = 7.56 – 7.53 (m, 1 H), 7.40 – 7.33 (m, 5 H), 6.81 (dd, J_1 = 8.9 Hz, J_2 = 2.9 Hz, 1 H), 6.60 (d, J = 2.9 Hz, 1 H), 6.06 (t, J = 4.5 Hz, 1 H), 4.44 (d, J = 4.5 Hz, 2 H), 4.26 (q, J = 7.1 Hz, 2 H), 3.69 (s, 3 H), 1.33 (t, J = 7.1 Hz, 3 H); ^{13}C NMR (100.6 MHz) ($CDCl_3$): δ = 156.2 (q), 154.4 (q), 138.9 (q), 138.8 (q), 130.51 (q), 130.47 (q), 128.8 (CH), 128.5 (CH), 127.9 (CH), 125.2 (CH), 113.0 (CH), 111.6 (CH), 62.1 (CH_2), 55.6 (CH_3), 43.2 (CH_2), 14.7 (CH_3); MS (EI ion source): *m/z* (%) = 309 (21, [M^+]), 280 (24), 236 (100), 193 (36), 165 (19), 63 (30); HRMS: *m/z* [M + H]$^+$ calcd for $C_{19}H_{20}NO_3$: 310.1438; found: 310.1443.

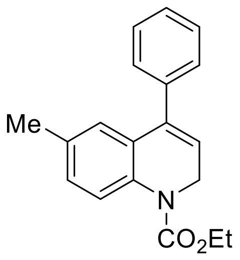

2e

Yield: 99% (101.4 mg);

2e: orange oil; IR (neat): 2981, 1697, 1493, 1378 cm^{-1}; ^{1}H NMR (400.13 MHz) ($CDCl_3$): δ = 7.53 – 7.52 (m, 1 H), 7.42 – 7.34 (m, 5 H), 7.07 (dd, J_1 = 8.3 Hz, J_2 = 1.4 Hz, 1 H), 6.86 (bd, J = 1.4 Hz, 1 H), 6.02 (t, J = 4.5 Hz, 1 H), 4.45 (d, J = 4.5 Hz, 2 H), 4.28 (q, J = 7.1 Hz, 2 H), 2.24 (s, 3 H), 1.34 (t, J = 7.1 Hz, 3 H); ^{13}C NMR (100.6 MHz) ($CDCl_3$): δ = 154.2 (q), 139.1 (q), 139.0 (q), 134.8 (q), 133.8 (q), 129.1 (q), 128.8 (CH), 128.5 (CH), 128.4 (CH), 127.8 (CH), 126.5 (CH), 124.0 (CH), 123.5 (CH), 62.2 (CH_2), 43.1 (CH_2), 21.1 (CH_3), 14.7 (CH_3); MS (EI ion source): *m/z* (%) = 293 (39, [M^+]), 264 (49), 220 (100), 204 (55), 63 (29); HRMS: *m/z* [M + H]$^+$ calcd for $C_{19}H_{20}NO_2$: 294.1489; found: 294.1491.

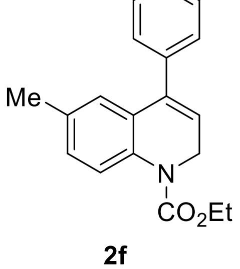

2f

Yield: 68% (77.2 mg); *n*-hexane/AcOEt mixture 80/20 (v/v) (R_f = 0.23)
2f: yellow oil; IR (neat): 2980, 2836, 1702, 1608, 1509, 1463 cm^{-1}; ^1H NMR (400.13 MHz) ($CDCl_3$): δ = 7.53 - 7.51 (m, 1 H), 7.28 (d, *J* = 8.8 Hz, 2 H), 7.07 (dd, J_1 = 8.3 Hz, J_2 = 1.6 Hz, 1 H), 6.93 (d, *J* = 8.8 Hz, 2 H), 6.89 (bd, *J* = 1.6 Hz, 1 H), 5.97 (t, *J* = 4.5 Hz, 1 H), 4.43 (d, *J* = 4.5 Hz, 2 H), 4.27 (q, *J* = 7.1 Hz, 2 H), 3.85 (s, 3 H), 2.24 (s, 3 H), 1.34 (t, *J* = 7.1 Hz, 3 H); ^{13}C NMR (100.6 MHz) ($CDCl_3$): δ = 159.3 (q), 154.2 (q), 138.5 (q), 134.8 (q), 133.7 (q), 131.4 (q), 129.9 (CH), 129.3 (CH), 128.3 (CH), 126.5 (CH), 123.9 (CH), 122.7 (q), 113.9 (CH), 62.1 (CH_2), 55.4 (CH_3), 43.1 (CH_2), 21.1 (CH_3), 14.7 (CH_3); MS (EI ion source): *m/z* (%) = 323 (43, [M^+]), 294 (87), 250 (100), 235 (20), 207 (17); HRMS: *m/z* $[M + H]^+$ calcd for $C_{20}H_{22}NO_3$: 324.1594; found: 324.1597.

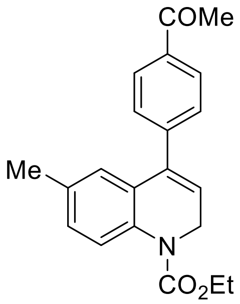

2g

Yield: 96% (112.4 mg);
2g: orange wax; IR (neat): 2980, 2243, 1705, 1596, 1494 cm^{-1}; ^1H NMR (400.13 MHz) ($CDCl_3$): δ = 7.99 (d, *J* = 8.4 Hz, 2 H), 7.54 – 7.52 (m, 1 H), 7.45 (d, *J* = 8.4 Hz, 2 H), 7.11 – 7.07 (m, 1 H), 6.79 (bd, *J* = 1.4 Hz, 1 H), 6.08 (t, *J* = 4.4 Hz, 1 H), 4.46 (d, *J* = 4.4 Hz, 2 H), 4.27 (q, *J* = 7.1 Hz, 2 H), 2.64 (s, 3 H), 2.23 (s, 3 H), 1.34 (t, *J* = 7.1 Hz, 3 H); ^{13}C NMR (100.6 MHz) ($CDCl_3$): δ = 197.9 (q), 154.2 (q), 144.0 (q), 138.3 (q), 136.5 (q), 134.8 (q), 133.9 (q), 129.6 (CH), 129.0 (CH), 128.7 (CH), 128.6 (CH), 128.5 (q), 126.3 (CH), 124.1 (CH), 62.3 (CH_2), 43.1 (CH_2), 26.8 (CH_3), 21.1 (CH_3), 14.7 (CH_3); MS (EI ion source): *m/z* (%) = 351 (M^+, 31), 322 (50), 278 (14), 157 (46), 134 (100), 114 (62); HRMS: *m/z* $[M + H]^+$ calcd for $C_{21}H_{22}NO_3$: 336.1594; found: 336.1598.

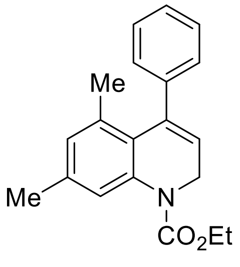

2h

Yield: 99% (106.3 mg);
2h: yellow oil; IR (neat): 2979, 1703, 1608, 1557, 1376, 1271 cm^{-1}; ^1H NMR (400.13 MHz) ($CDCl_3$): δ = 7.28 – 7.14 (m, 6 H), 6.68 (s, 1 H), 6.01 (t, *J* = 5.2 Hz, 1 H), 4.20 – 4.14 (m, 4 H), 2.26 (s, 3 H), 1.65 (s, 3 H), 1.24 (t, *J* = 7.1 Hz, 3 H); ^{13}C NMR (100.6 MHz) ($CDCl_3$): δ = 153.9 (q), 141.8 (q), 139.9 (q), 139.1 (q), 137.3 (q), 135.3 (q), 129.0 (CH), 128.5 (CH), 127.4 (CH), 127.2 (CH), 125.8 (q), 125.3 (CH), 122.4 (CH), 62.1 (CH_2), 42.6 (CH_2), 23.0 (CH_3), 21.5 (CH_3), 14.7 (CH_3); MS (EI ion source): *m/z* (%) = 307 (20, [M^+]), 278 (27), 234 (100), 218 (18); HRMS: *m/z* $[M + H]^+$ calcd for $C_{20}H_{22}NO_2$: 308.1645; found: 308.1649.

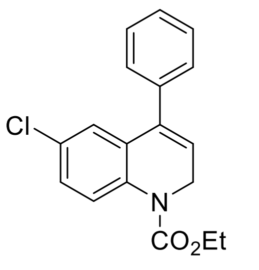

2i

Yield: 56% (61.7 mg); *n*-hexane/AcOEt mixture 95/5 (v/v) (R_f = 0.21)

2i: pale yellow oil; IR (neat): 2981, 2847, 1702, 1594, 1481 cm^{-1}; ^1H NMR (400.13 MHz) ($CDCl_3$): δ = 7.59 (bd, *J* = 8.1 Hz, 1 H), 7.43 – 7.36 (m, 3 H), 7.33 – 7.30 (m, 2 H), 7.21 (dd, J_1 = 8.7 Hz, J_2 = 2.5 Hz, 1 H), 7.02 (d, *J* = 2.5 Hz, 1 H), 6.06 (t, *J* = 4.5 Hz, 1 H), 4.47 (d, *J* = 4.5 Hz, 2 H), 4.28 (q, *J* = 7.1 Hz, 2 H), 1.34 (t, *J* = 7.1 Hz, 3 H); ^{13}C NMR (100.6 MHz) ($CDCl_3$): δ = 153.9 (q), 138.1 (q), 135.6 (q), 130.7 (q), 129.5 (CH), 128.61 (CH), 128.60 (CH), 128.0 (CH), 127.4 (CH), 125.7 (CH), 125.3 (CH), 124.4 (q), 62.3 (CH_2), 43.0 (CH_2), 14.5 (CH_3). MS (EI ion source): *m/z* (%) = 313 (30, [M^+]), 284 (72), 240 (100), 204 (62), 176 (19); HRMS: *m/z* $[M + H]^+$ calcd for $C_{18}H_{17}ClNO_2$: 314.0942; found: 314.0952.

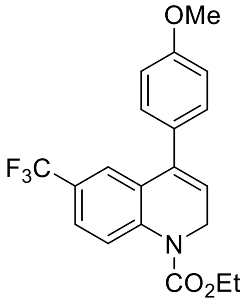

2j

Yield: 10% (13.0 mg); *n*-hexane/AcOEt mixture 90/10 (v/v) (R_f = 0.24)

2j: colorless oil; IR (neat): 2919, 2848, 1710, 1609, 1382, 1051 cm^{-1}; ^1H NMR (400.13 MHz) ($CDCl_3$): δ = 7.77 (bd, *J* = 8.5 Hz, 1 H), 7.49 (dd, J_1 = 8.6 Hz, J_2 = 1.6 Hz, 1 H), 7.33 (bd, *J* = 1.6 Hz, 1 H), 7.25 (d, *J* = 8.8 Hz, 2 H), 6.94 (d, *J* = 8.8 Hz, 2 H), 6.04 (t, *J* = 4.5 Hz, 1 H), 4.48 (d, *J* = 4.5 Hz, 2 H), 4.30 (q, *J* = 7.1 Hz, 2 H), 3.86 (s, 3 H), 1.36 (t, *J* = 7.1 Hz, 3 H); ^{13}C NMR (100.6 MHz) ($CDCl_3$): δ = 159.7 (q), 153.9 (q), 140.4 (q), 137.8 (q), 130.4 (q), 129.9 (q), 129.7 (CH), 126.1 (q, q, *J* = 32.5 Hz), 124.5 (CH, q, *J* = 3.7 Hz), 124.20 (q, q, *J* = 270.3 Hz), 124.19 (CH), 123.7 (CH), 123.1 (CH, q, *J* = 3.7 Hz), 114.2 (CH), 62.7 (CH_2), 55.5 (CH_3), 43.2 (CH_2), 14.6 (CH_3); ^{19}F NMR (376.5 MHz) ($CDCl_3$): δ = -62.3; MS (EI ion source): *m/z* (%) = 377 (26, [M^+]), 348 (77), 304 (100), 289 (14), 261 (18); HRMS: *m/z* $[M + Na]^+$ calcd for $C_{20}H_{18}F_3NO_3Na$: 400.1131; found: 400.1119.

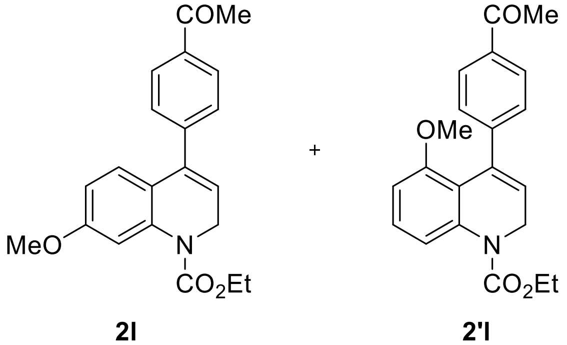

2l **2'l**

HPLC eluent = *n*-hexane/AcOEt mixture 85/15 (v/v) *(R_f = 0.22)*
Overall yield (catalyst A): 99% (121.5 mg); **2l/2'l =** 61/39
Overall yield (catalyst A'): 99 % (120.9); **2l/2'l =** 75/29
Overall yield (catalyst B): 99% (121.4 mg); **2l/2'l =** 54/46
Overall yield (catalyst C): 86% (105.6 mg); **2l/2'l =** 54/46
2l + 2'l: (isomeric mixture): yellow wax**;** IR (KBr): 3060, 2922, 1680, 1593, 1480, 1232 cm^{-1};
reported NMR spectra refer to an isomeric mixture **2l + 2'l** in the ratio 54/46; ^{1}H NMR signals have been assigned to each specific isomer while ^{13}C NMR signals have not been assigned.
^{1}H NMR (400.13 MHz) ($CDCl_3$) (selected signals): δ = 7.89 (d, *J* = 8.4 Hz, 2 H **2l**), 7.81 (d, *J* = 8.4 Hz, 2 H **2'l**), 7.35 (d, *J* = 8.4 Hz, 2 H **2l**), 7.24 – 7.17 (m, 1 H **2l** + 4 H **2'l**), 6.84 (d, *J* = 8.6 Hz, 1 H **2l**), 6.59 (d, *J* = 9.0 Hz, 1 H **2'l**), 6.52 (dd, J_1 = 8.6 Hz, J_2 = 2.6 Hz, 1 H **2l**), 6.03 (t, *J* = 5.0 Hz, 1 H **2'l**), 5.87 (t, *J* = 4.6 Hz, 1 H **2l**), 4.39 (d, *J* = 4.6 Hz, 2 H **2l**), 4.25 – 4.15 (m, 2 H **2l** + 4 H **2'l**), 3.74 (s, 3 H **2l**), 3.32 (s, 3 H **2'l**), 2.54 (s, 3 H **2l**), 2.53 (s, 3 H **2'l**), 1.29 – 1.23 (m, 3 H **2l** + 3 H **2'l**); ^{13}C NMR (100.6 MHz) ($CDCl_3$): δ = 198.1, 197.9, 159.4, 156.0, 154.1, 153.9, 146.7, 144.1, 139.8, 138.7, 138.1, 137.1, 136.6, 135.5, 129.0, 128.8, 128.7, 128.0, 126.93, 126.90, 126.0, 121.9, 121.7, 117.9, 117.1, 110.4, 109.9, 108.2, 62.43, 62.39, 55.6, 55.4, 43.2, 42.7, 26.85, 26.79, 14.8, 14.7; MS (EI ion source): *m/z* (%) = 351 (51, [M^+]), 322 (63), 278 (100), 262 (23), 235 (20), 43 (23); HRMS: *m/z* [M + H]$^+$ calcd for $C_{21}H_{22}NO_4$: 352.1543; found: 352.1536.

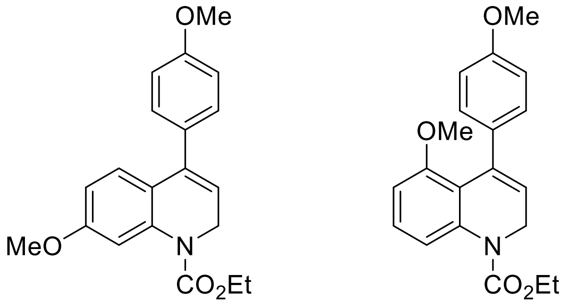

2m **2'm**

HPLC eluent = *n*-hexane/AcOEt mixture 85/15 (v/v) *(R_f = 0.23)*
Overall yield (catalyst A): 70% (83.0 mg); **2m/2'm =** 91/9
Overall yield (catalyst A'): 73% (86.5 mg); **2m/2'm =** 91/9
Overall yield (catalyst B): 83% (98.6 mg); **2m/2'm =** 63/37
Overall yield (catalyst C): 77% (91.8 mg); **2m/2'm =** 51/49
2m: colorless oil; IR (neat): 2915, 1711, 1577, 1386, 1244 cm^{-1}; ^{1}H NMR (400.13 MHz) ($CDCl_3$): δ = 7.27 – 7.24 (m, 3 H), 7.00 (d, *J* = 8.6 Hz, 1 H), 6.91 (d, *J* = 8.8 Hz, 2 H), 6.60 (dd, J_1 = 8.6 Hz, J_2 = 2.6 Hz, 1 H), 5.84 (t, *J* = 4.5 Hz, 1 H), 4.43 (d, *J* = 4.5 Hz, 2 H), 4.28 (q, *J* = 7.1 Hz, 2 H), 3.84 (s, 3 H), 3.82 (s, 3 H), 1.35 (t, *J* = 7.1 Hz, 3 H); ^{13}C NMR (100.6 MHz) ($CDCl_3$): δ = 159.3 (q), 159.0 (q), 154.1 (q), 138.6 (q), 138.2 (q), 131.6 (q), 129.9 (CH), 127.0 (CH), 122.7 (q), 119.6 (CH), 113.9 (CH), 110.2 (CH), 109.6 (CH), 62.2 (CH_2), 55.5 (CH_3), 55.4 (CH_3), 43.2 (CH_2), 14.7 (CH_3); MS (EI ion source): *m/z* (%) = 339 (1, [M^+]), 265 (100), 250 (13), 222 (10), 207 (15); HRMS: *m/z* [M + Na]$^+$ calcd for $C_{20}H_{21}NO_4Na$: 362.1363; found: 362.1357.

2'm: colorless oil; IR (neat): 2915, 1694, 1609, 1381, 1239, 1042 cm^{-1}; ^{1}H NMR (400.13 MHz) ($CDCl_3$): δ = 7.26 – 7.24 (m, 1 H), 7.17 (t, *J* = 8.2 Hz, 1 H), 7.08 (d, *J* = 8.8 Hz, 2 H), 6.75 (d, *J* = 8.8 Hz, 2 H), 6.60 (d, *J* = 8.9 Hz, 1 H), 5.93 (t, *J* = 5.1 Hz, 1 H), 4.20 (d, *J* = 5.1 Hz, 2 H), 4.18 (q, *J* = 7.1 Hz, 2 H), 3.75 (s, 3 H), 3.37 (s, 3 H), 1.25 (t, *J* = 7.1 Hz, 3 H); ^{13}C NMR (100.6 MHz) ($CDCl_3$): δ = 158.6 (q), 156.3 (q), 153.9 (q), 140.0 (q), 137.2 (q), 134.1 (q), 128.3 (CH), 127.8 (CH), 123.6 (CH), 118.7 (q), 117.0 (CH), 113.1 (CH), 108.4 (CH), 62.3 (CH_2), 55.7 (CH_3), 55.5 (CH_3), 42.7 (CH_2), 14.7 (CH_3); MS (EI ion source): *m/z* (%) = 339 (66, [M^+]), 310 (67), 266 (100), 251 (23); HRMS: *m/z* [M + H]$^+$ calcd for $C_{20}H_{22}NO_4$: 340.1543; found: 340.1539.

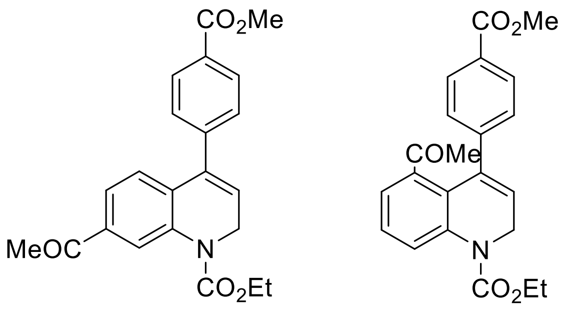

2n **2'n**

HPLC eluent = *n*-hexane/AcOEt mixture 80/20 (v/v) *(R_f = 0.23)*
Overall yield (catalyst A): 85% (112.9 mg); **2n/2'n =** 88/12
Overall yield (catalyst A'): 75% (99.7); **2n/2'n =** 64/36
Overall yield (catalyst B): 99% (131.6 mg); **2n/2'n =** 40/60
Overall yield (catalyst C): 72% (95.1 mg); **2n/2'n =** 19/81
2n: pale yellow solid; mp = 140 – 141 °C; IR (neat): 2930, 1751, 1657, 1583, 1298 cm^{-1}; ^{1}H NMR (400.13 MHz) ($CDCl_3$): δ = 8.27 (bs, 1 H), 8.08 (d, *J* = 8.4 Hz, 2 H), 7.62 (dd, J_1 = 8.2 Hz, J_2 = 1.7 Hz, 1 H), 7.41 (d, *J* = 8.4 Hz, 2 H), 7.08 (d, *J* = 8.2 Hz, 1 H), 6.21 (t, *J* = 4.5 Hz, 1 H), 4.53 (d, *J* = 4.5 Hz, 2 H), 4.31 (q, *J* = 7.1 Hz, 2 H), 3.94 (s, 3 H), 2.60 (s, 3 H), 1.37 (t, *J* = 7.1 Hz, 3 H); ^{13}C NMR (100.6 MHz) ($CDCl_3$): δ = 197.3 (q), 166.7 (q), 153.9 (q), 142.8 (q), 137.7 (q), 137.3 (q), 136.4 (q), 132.6, (q) 129.9 (CH), 129.8 (CH), 128.7 (CH), 126.9 (q), 125.9 (CH), 124.4 (CH), 123.9 (CH), 62.5 (CH_2), 52.2 (CH_3), 43.0 (CH_2), 26.7 (CH_3), 14.5 (CH_3); MS (EI ion source): *m/z* (%) = 379 (67, [M^+]), 350 (15), 306 (100), 290 (52), 264 (70), 204 (26); HRMS: *m/z* [M + Na]$^+$ calcd for $C_{22}H_{21}NO_5Na$: 402.1312; found: 402.1312.

2'n: yellow oil; IR (neat): 2919, 1725, 1599, 1268, 1023 cm^{-1}; ^{1}H NMR (400.13 MHz) ($CDCl_3$): δ = 7.90 (d, *J* = 8.5 Hz, 2 H), 7.77 (m, 1 H), 7.30 (t, *J* = 7.8 Hz, 1 H), 7.23 – 7.19 (m, 3 H), 6.21 (t, *J* = 5.2 Hz, 1 H), 4.33 (d, *J* = 5.2 Hz, 2 H), 4.21 (q, *J* = 7.1 Hz, 2 H), 3.84 (s, 3 H), 2.00 (s, 3 H), 1.27 (t, *J* = 7.1 Hz, 3 H); ^{13}C NMR (100.6 MHz) ($CDCl_3$): δ = 202.2 (q), 166.7 (q), 153.6 (q), 145.4 (q), 139.8 (q), 139.5 (q), 138.0 (q), 129.9 (q), 129.1 (CH), 127.6 (CH), 127.3 (CH), 127.1 (q), 126.7 (CH), 123.9 (CH), 62.5 (CH_2), 52.1 (CH_3), 42.4 (CH_2), 29.1 (CH_3), 14.5 (CH_3); MS (EI ion source): *m/z* (%) = 379 (58, [M^+]), 350 (100), 306 (95), 264 (44), 204 (36), 43 (27); HRMS: *m/z* [M + Na]$^+$ calcd for $C_{22}H_{21}NO_5Na$: 402.1312; found: 402.1310.

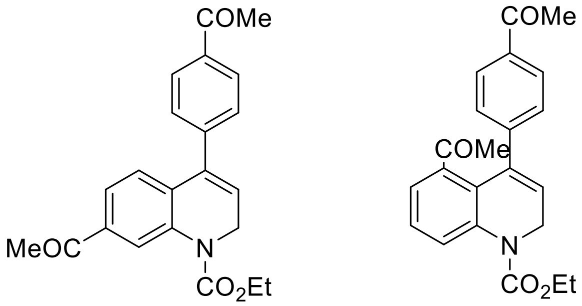

2o **2'o**

HPLC eluent = *n*-hexane/AcOEt mixture 75/25 (v/v) *(R_f = 0.22)*
Overall yield (catalyst A): 88% (112.0 mg); **2o/2'o =** 88/12
Overall yield (catalyst A'): 90% (114.3); **2o/2'o =** 65/35
Overall yield (catalyst B): 70% (89.0 mg); **2o/2'o =** 33/67
Overall yield (catalyst C): 82% (104.4 mg); **2o/2'o =** 20/80

2o: yellow wax; IR (neat): 2982, 1680, 1607, 1556, 1256 cm^{-1}; ^{1}H NMR (400.13 MHz) ($CDCl_3$): δ = 8.27 (bs, 1 H), 8.00 (d, *J* = 8.4 Hz, 2 H), 7.63 (dd, J_1 = 8.2 Hz, J_2 = 1.7 Hz, 1 H), 7.43 (d, *J* = 8.4 Hz, 2 H), 7.08 (d, *J* = 8.2 Hz, 1 H), 6.22 (t, *J* = 4.6 Hz, 1 H), 4.54 (d, *J* = 4.6 Hz, 2 H), 4.32 (q, *J* = 7.1 Hz, 2 H), 2.64 (s, 3 H), 2.60 (s, 3 H), 1.37 (t, *J* = 7.1 Hz, 3 H); ^{13}C NMR (100.6 MHz) ($CDCl_3$): δ = 197.8 (q), 197.5 (q), 154.0 (q), 143.1 (q), 137.9 (q), 137.5 (q), 136.9 (q), 136.7 (q), 132.7 (q), 129.1 (CH), 128.9 (CH), 127.2 (CH), 126.1 (CH), 124.6 (CH), 124.0 (CH), 62.7 (CH_2), 43.3 (CH_2), 26.9 (CH_3, 2C), 14.7 (CH_3); MS (EI ion source): *m/z* (%) = 363 (62, [M^+]), 333 (12), 290 (83), 248 (70), 43 (100); HRMS: *m/z* $[M + Na]^+$ calcd for $C_{22}H_{21}NO_4Na$: 386.1363; found: 386.1359.

2'o: yellow wax; IR (neat): 2981, 1682, 1603, 1450, 1376, 1265 cm^{-1}; ^{1}H NMR (400.13 MHz) ($CDCl_3$): δ = 7.89 (d, *J* = 8.6 Hz, 2 H), 7.85 (m, 1 H) 7.38 (t, *J* = 7.7 Hz, 1 H), 7.31 – 7.27 (m, 3 H), 6.28 (t, *J* = 5.2 Hz, 1 H), 4.40 (d, *J* = 5.2 Hz, 2 H), 4.28 (q, *J* = 7.1 Hz, 2 H), 2.59 (s, 3 H), 2.07 (s, 3 H), 1.34 (t, *J* = 7.1 Hz, 3 H); ^{13}C NMR (100.6 MHz) ($CDCl_3$): δ = 202.3 (q), 197.6 (q), 153.7 (q), 145.8 (q), 139.9 (q), 139.6 (q), 138.1 (q), 136.1 (q), 128.8 (CH), 127.7 (CH), 127.39 (CH), 127.34 (q), 126.9 (CH), 124.1 (CH), 62.6 (CH_2), 42.5 (CH_2), 29.3 (CH_3), 26.7 (CH_3), 14.6 (CH_3); MS (EI ion source): *m/z* (%) = 363 (26, [M^+]), 334 (41), 290 (54), 232 (28), 204 (30), 43 (100); HRMS: *m/z* $[M + Na]^+$ calcd for $C_{22}H_{21}NO_4Na$: 386.1363; found: 386.1361.

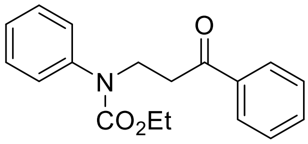

3a

Yield: 75% (78.2 mg); *n*-hexane/AcOEt mixture 65/35 (v/v) *(R_f = 0.27)*
3a: yellow oil; IR (neat): 2980, 1681, 1597, 1496, 1298, 1023 cm^{-1}; ^{1}H NMR (400.13 MHz) ($CDCl_3$): δ = 7.95 (d, *J* = 7.7 Hz, 2 H), 7.57 (t, *J* = 7.4 Hz, 1 H), 7.46 (t, *J* = 7.7 Hz, 2 H), 7.38 (t, *J* = 7.7 Hz, 2 H), 7.28 – 7.23 (m, 3 H), 4.21 – 4.12 8m, 4 H), 3.34 (t, *J* = 7.6 Hz, 2 H), 1.23 (t, *J* = 7.0 Hz, 3 H); ^{13}C NMR (100.6 MHz) ($CDCl_3$): δ = 198.4 (q), 155,6 (q), 141.9 (q), 136.7 (q), 133.3 (CH), 129.1 (CH), 128.7 (CH), 128.1 (CH), 127.2 (CH), 126.7 (CH), 61.8 (CH_2), 46.6 (CH_2), 37.4 (CH_2), 14.6 (CH_3); HRMS: *m/z* $[M + Na]^+$ calcd for $C_{18}H_{19}NO_3Na$: 320.1257; found: 320.1253.

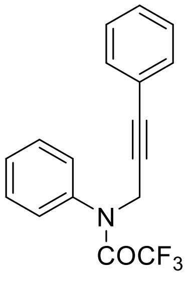

4a

Yield: 71 %; *n*-hexane/AcOEt mixture 85/15 (v/v) *(R_f = 0.25)*
4a: yellow oil; IR (neat): 2980, 2238, 1694, 1595, 1491, 1314, 1206, 1181 cm^{-1}; ^{1}H NMR (400.13 MHz) ($CDCl_3$): δ = 7.50 – 7.49 (m, 3 H), 7.42 – 7.28 (m, 7 H), 4.78 (s, 2 H); ^{13}C NMR (100.6 MHz) ($CDCl_3$): δ =156.6 (q, q, *J* = 36.0 Hz), 138.3 (q), 131.7 (CH), 129.4 (CH), 128.71 (CH), 128.69 (CH), 128.3 (CH), 122.2 (q), 116.3 (q, q, *J* = 286.5 Hz), 113.7 (CH), 85.6 (q), 82.2 (q), 41.8 (CH_2); ^{19}F NMR (376.5 MHz) ($CDCl_3$): δ = -67.2; HRMS: *m/z* $[M + Na]^+$ calcd for $C_{17}H_{12}F_3NONa$: 326.0763; found: 326.0764.

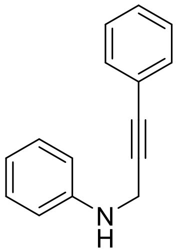

4b

Yield: 85%; *n*-hexane/AcOEt mixture 75/25 (v/v) *(R_f = 0.27)*

4b: known compound; [3] yellow oil; IR (neat): 3406, 3052, 2980, 2241, 1601, 1489, 1313, 1180 cm^{-1}; ^1H NMR (400.13 MHz) ($CDCl_3$): δ = 7.46 – 7.44 (m, 2 H), 7.34 – 7.26 (m, 5 H), 6.84 (t, *J* = 7.3 Hz, 1 H), 6.78 (d, *J* = 7.8 Hz, 2 H), 4.19 (s, 2 H), 4.00 (bs, 1 H); ^{13}C NMR (100.6 MHz) ($CDCl_3$): δ = 147.2 (q), 131.7 (CH), 129.3 (CH), 128.30 (CH), 138.27 (CH), 122.9 (q), 118.5 (CH), 113.6 (CH), 86.4 (q), 83.3 (q), 34.6 (CH_2); HRMS: *m/z* $[M + Na]^+$ calcd for $C_{15}H_{13}NNa$: 230.0940; found: 230.0940.

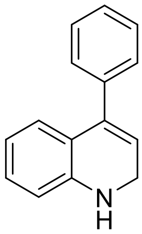

5b

Yield: 25% (18.1 mg); *n*-hexane/AcOEt mixture 85/15 (v/v) *(R_f = 0.25)*

5b: known compound; [4] pale yellow oil; IR (neat): 3057, 1955, 1603, 1583, 1490, 1389 cm^{-1}; ^1H NMR (400.13 MHz) ($CDCl_3$): δ = 7.62 – 7.56 (m, 1 H), 7.42 – 7.35 (m, 5 H), 7.02 (t, *J* = 7.6 Hz, 1 H), 6.86 (d, *J* = 7.6 Hz, 1 H), 6.60 (t, *J* = 7.5 Hz, 1 H), 6.54 (d, *J* = 7.9 Hz, 1 H), 5.71 (t, *J* = 4.2 Hz, 1 H), 4.24 (d, *J* = 4.2 Hz, 2 H), 3.70 (bs, 1 H), ; ^{13}C NMR (100.6 MHz) ($CDCl_3$): δ = 145.6 (q), 139.7 (q), 138.4 (q), 128.8 (CH), 128.7 (CH), 128.2 (CH), 127.3 (CH), 126.1 (CH), 122.4 (q), 120.9 (CH), 117.9 (CH), 113.3 (CH), 43. (CH_2); HRMS: *m/z* $[M + H]^+$ calcd for $C_{15}H_{14}N$: 208.1121; found: 208.1120.

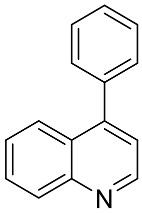

6b

Yield: 56% (40.0 mg); *n*-hexane/AcOEt mixture 85/15 (v/v) *(R_f = 0.23)*

6b: known compound; [5] lit. mp = 62 – 64 °C; pale yellow solid; mp = 62 – 63°C; IR (neat): 1919, 1611, 1572, 1583, 1489, 1388 cm^{-1}; ^1H NMR (400.13 MHz) ($CDCl_3$): δ = 8.95 (d, *J* = 4.4 Hz, 1 H), 8.18 (d, *J* = 8.4 Hz, 1 H), 7.93 (d, *J* = 8.4 Hz, 1 H), 7.73 (t, *J* = 7.2 Hz, 1 H), 7.56 – 7.49 (m, 6 H), 7.34 (d, *J* = 4.4 Hz, 1 H); ^{13}C NMR (100.6 MHz) ($CDCl_3$): δ = 150.1 (CH), 148.8 (q), 148.6 (q), 138.1 (q), 130.0 (CH), 129.7 (CH), 129.5 (CH), 128.7 (CH), 128.6 (CH), 126.9 (q), 126.8 (CH), 126.0 (CH), 121.5 (CH); HRMS: *m/z* $[M + H]^+$ calcd for $C_{15}H_{12}N$: 206.0964; found: 206.0965.

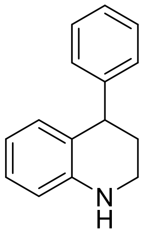

7b

Yield: 7% (5.1 mg); *n*-hexane/AcOEt mixture 85/15 (v/v) *(R_f* = 0.28)
7b: known compound;[4] pale yellow oil; IR (neat): 3415, 2920, 2832, 1607, 1504, 1316 cm^{-1}; ^{1}H NMR (400.13 MHz) ($CDCl_3$): δ = 7.30 (t, *J* = 7.3 Hz, 2 H), 7.21 (t, *J* = 7.3 Hz, 1 H), 7.15 (d, *J* = 7.4 Hz, 2 H), 7.02 (t, *J* = 7.4 Jz, 1 H), 6.76 (d, *J* = 7.4 Hz, 1 H), 6.59 – 6.55 (m, 2 H), 4.15 (t, *J* = 6.1 Hz, 1 H), 3.95 (bs, 1 H), 3.34 – 3.21 (m, 2 H), 2.26 – 2.19 (m, 1 H), 2.10 – 2.02 (m, 1 H), ; ^{13}C NMR (100.6 MHz) ($CDCl_3$): δ = 146.8 (q), 145.1 (q), 130.6 (CH), 128.8 (CH), 128.4 (CH), 127.4 (CH), 126.2 (CH), 123.5 (q), 117.1 (CH), 114.3 (CH), 42.9 (CH), 39.3 (CH_2), 31.2 (CH_2); HRMS: *m/z* $[M + H]^+$ calcd for $C_{15}H_{16}N$: 210.1277; found: 210.1277.

References

1. Di Nicola, A.; Arcadi, A.; Rossi, L., BMIm HCO3: an ionic liquid with carboxylating properties. Synthesis of carbamate esters from amines. *New Journal of Chemistry* **2016,** *40* (12), 9895-9898.
2. Tam, T. F.; Thomas, E.; Krantz, A., Synthesis of 5-alkynylidene-oxazolidin-2-ones. *Tetrahedron Letters* **1987,** *28* (11), 1127-1130.
3. Buzzetti, L.; Puriņš, M.; Greenwood, P. D. G.; Waser, J., Enantioselective Carboetherification/Hydrogenation for the Synthesis of Amino Alcohols via a Catalytically Formed Chiral Auxiliary. *Journal of the American Chemical Society* **2020,** *142* (41), 17334-17339.
4. Pearson, W. H.; Fang, W.-K., Reactions of Azides with Electrophiles: New Methods for the Generation of Cationic 2-Azabutadienes. Synthesis of 1,2, 3,4-Tetrahydroquinolines and 1,2-Dihydroquinolines via a Hetero Diels–Alder Reaction. **1997,** *37* (1), 39-46.
5. Panda, S.; Coffin, A.; Nguyen, Q. N.; Tantillo, D. J.; Ready, J. M., Synthesis and Utility of Dihydropyridine Boronic Esters. **2016,** *55* (6), 2205-2209.

^{1}H, ^{13}C ^{19}F NMR SPECTRA OF COMPOUNDS 2a – j, 2k – o, 2’k – o, 3a, 5b, 6b, 7b

^{1}H NMR (400.13 MHz), $CDCl_3$ (2a)

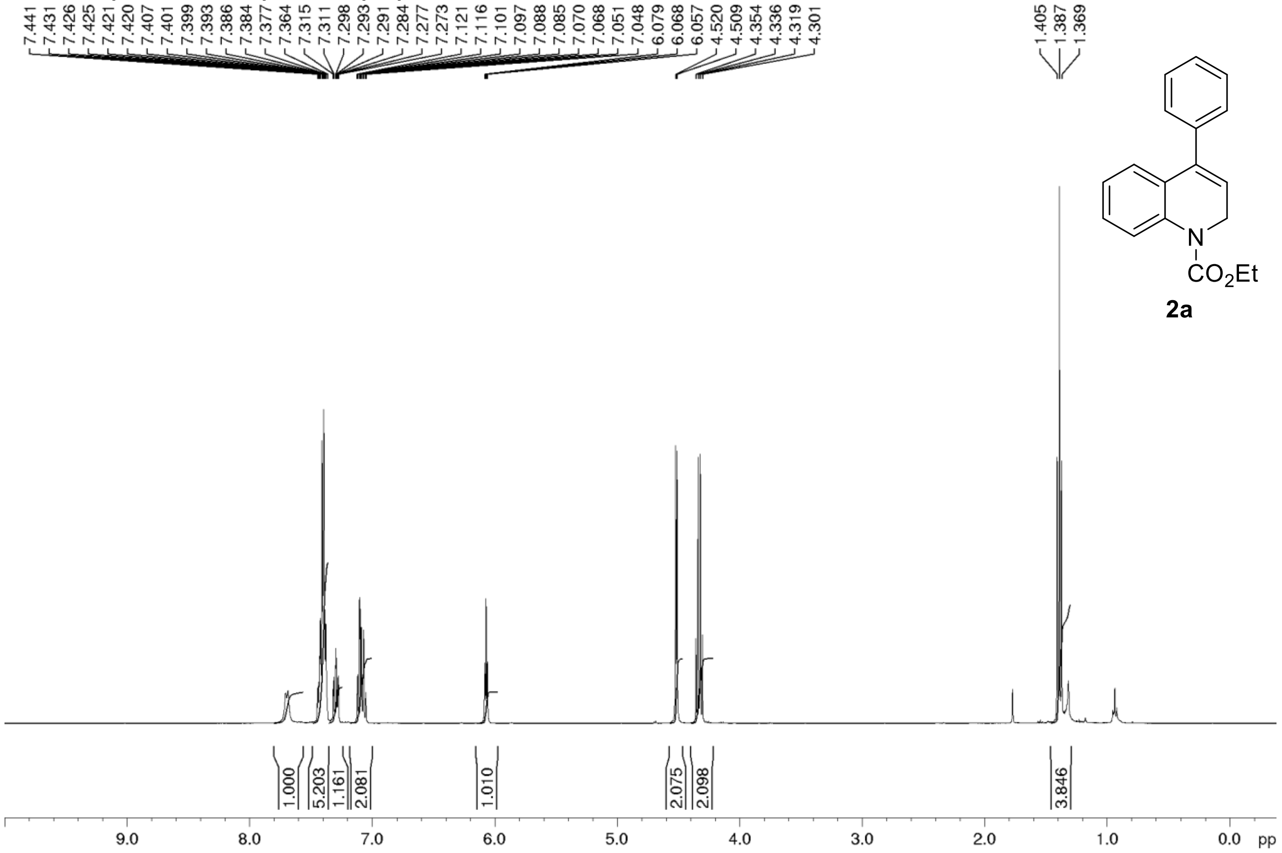

2a

^{13}C NMR (100.6 MHz), $CDCl_3$ (2a)

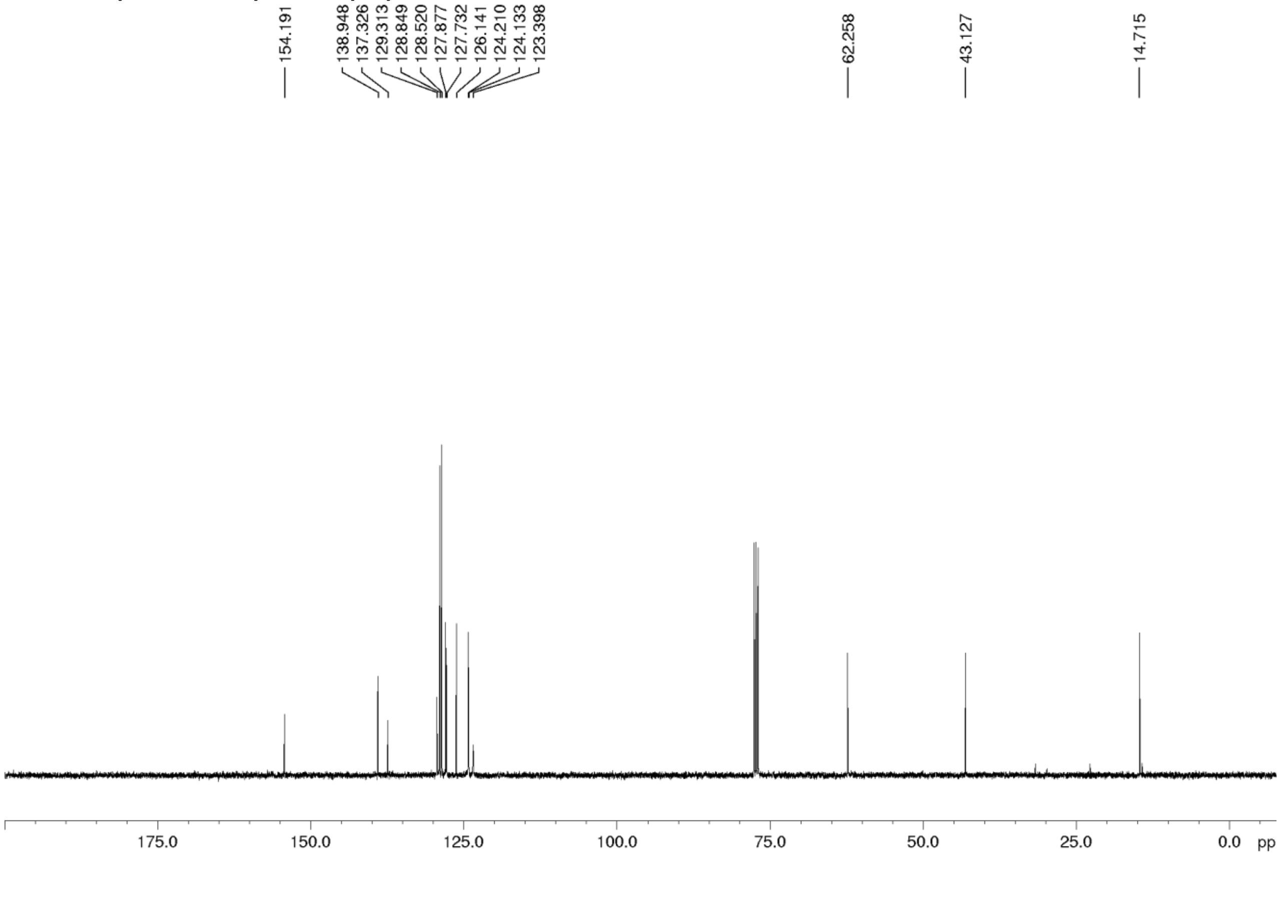

^{1}H NMR (400.13 MHz), $CDCl_3$ (2b)

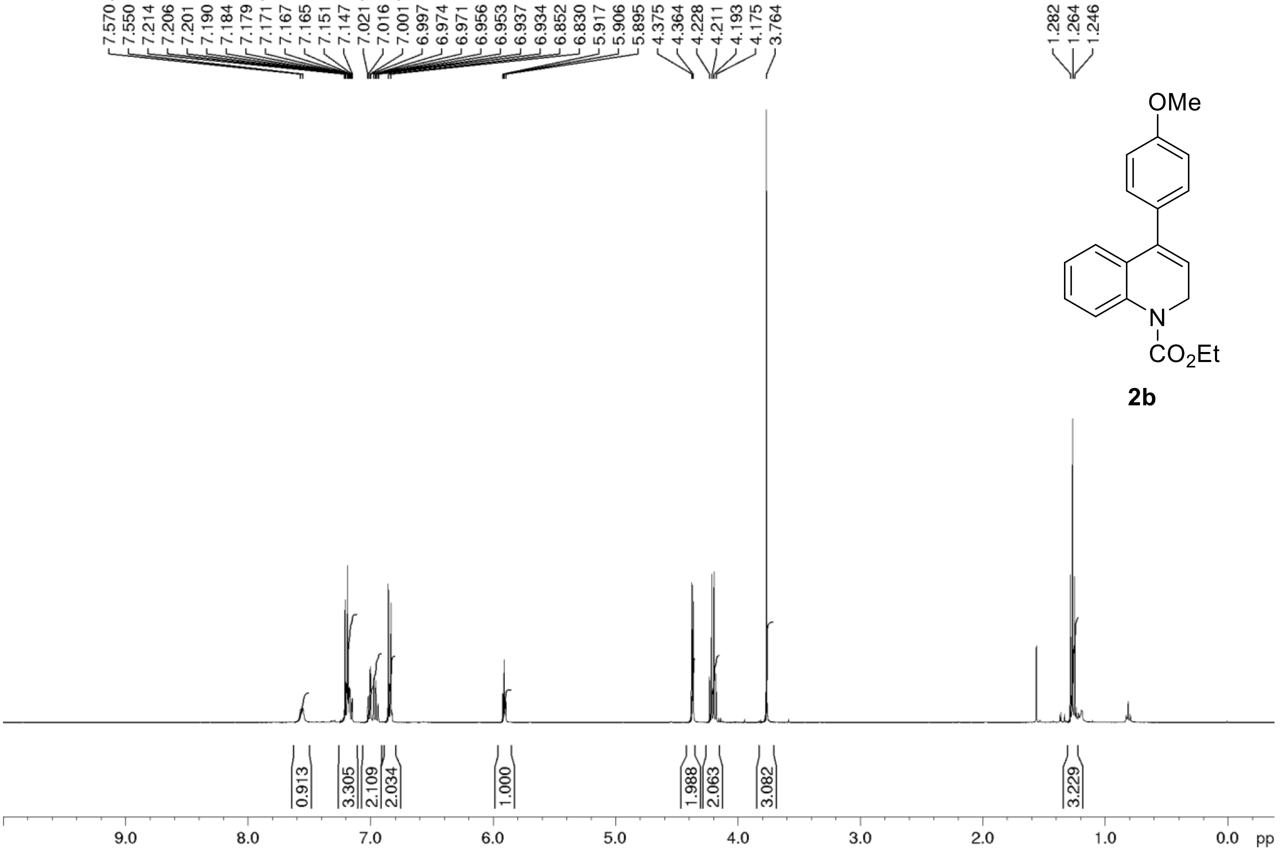

2b

^{13}C NMR (100.6 MHz), $CDCl_3$ (2b)

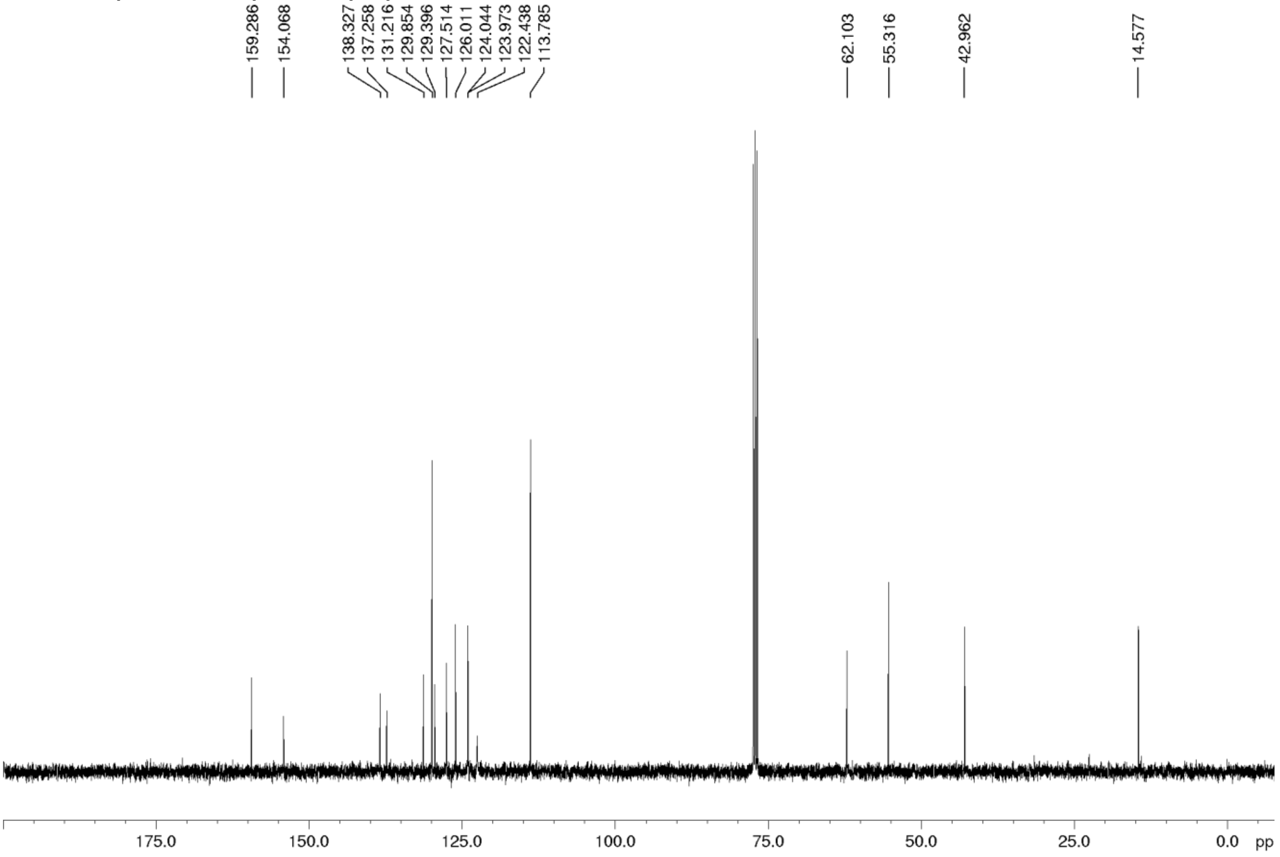

^{1}H NMR (400.13 MHz), $CDCl_3$ (2c)

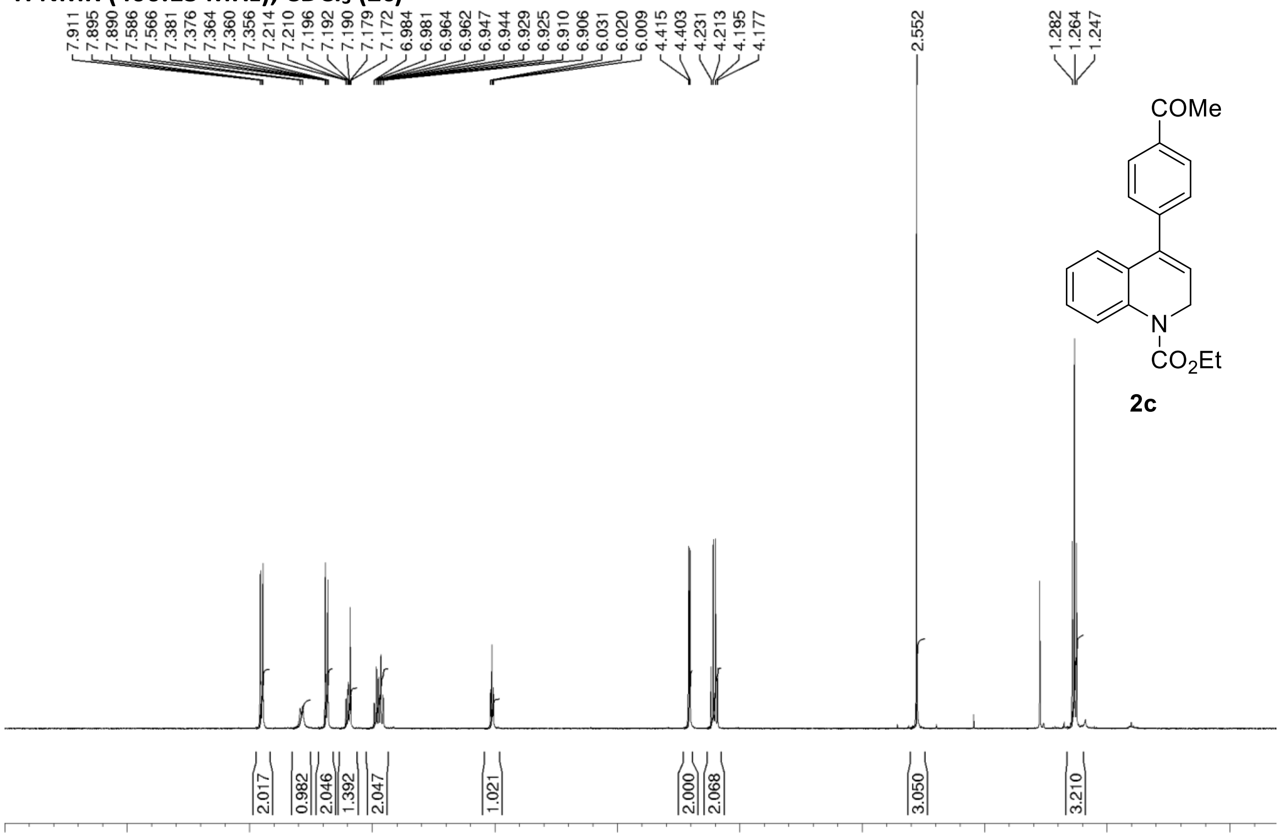

^{13}C NMR (100.6 MHz), $CDCl_3$ (2c)

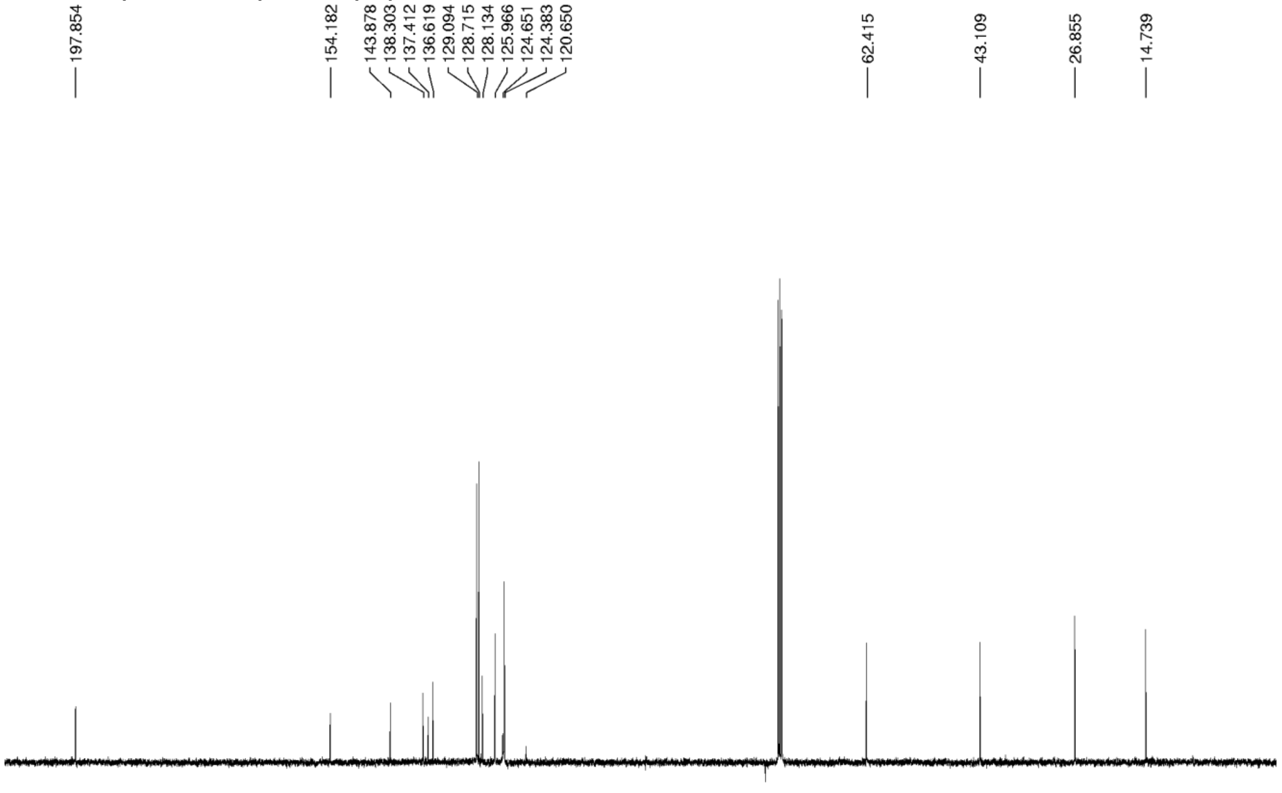

^{1}H NMR (400.13 MHz), $CDCl_3$ (2d)

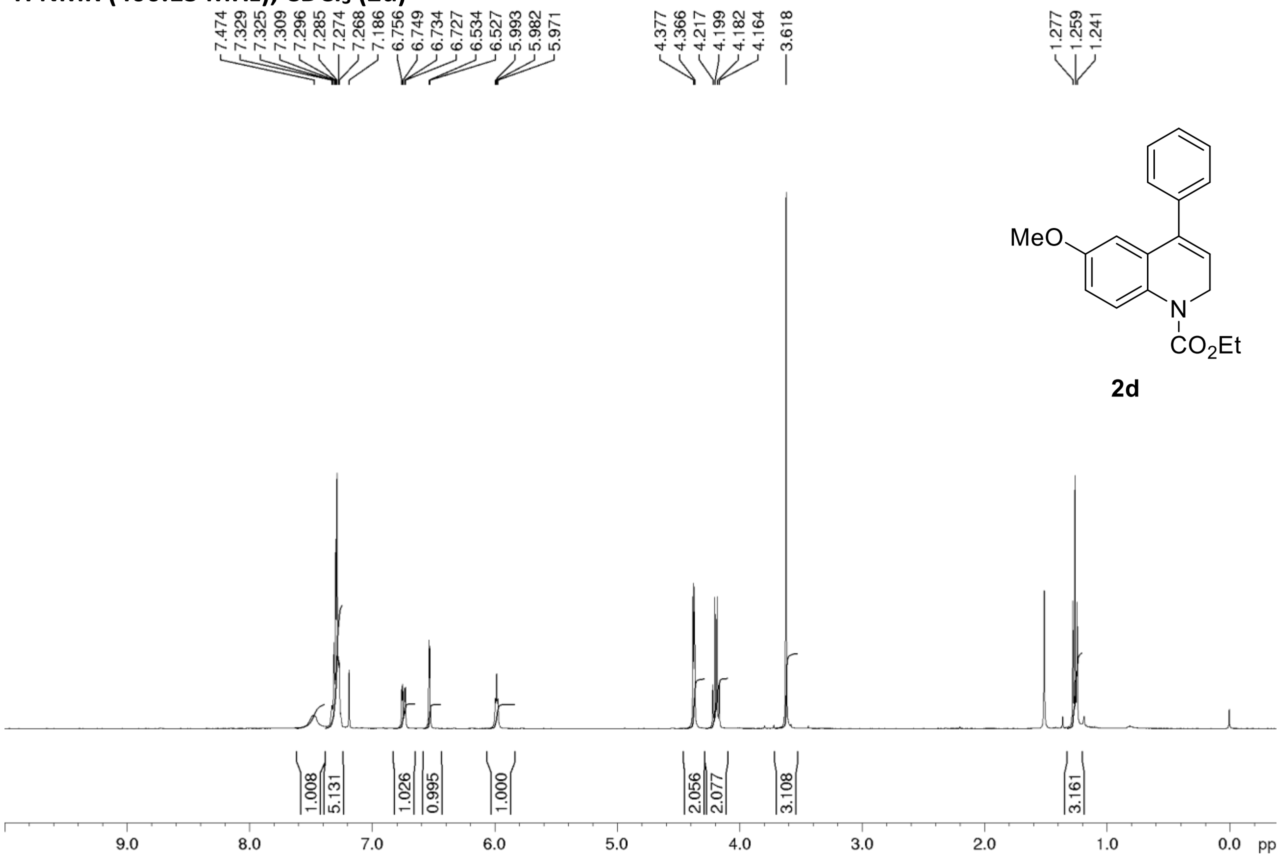

^{13}C NMR (100.6 MHz), $CDCl_3$ (2d)

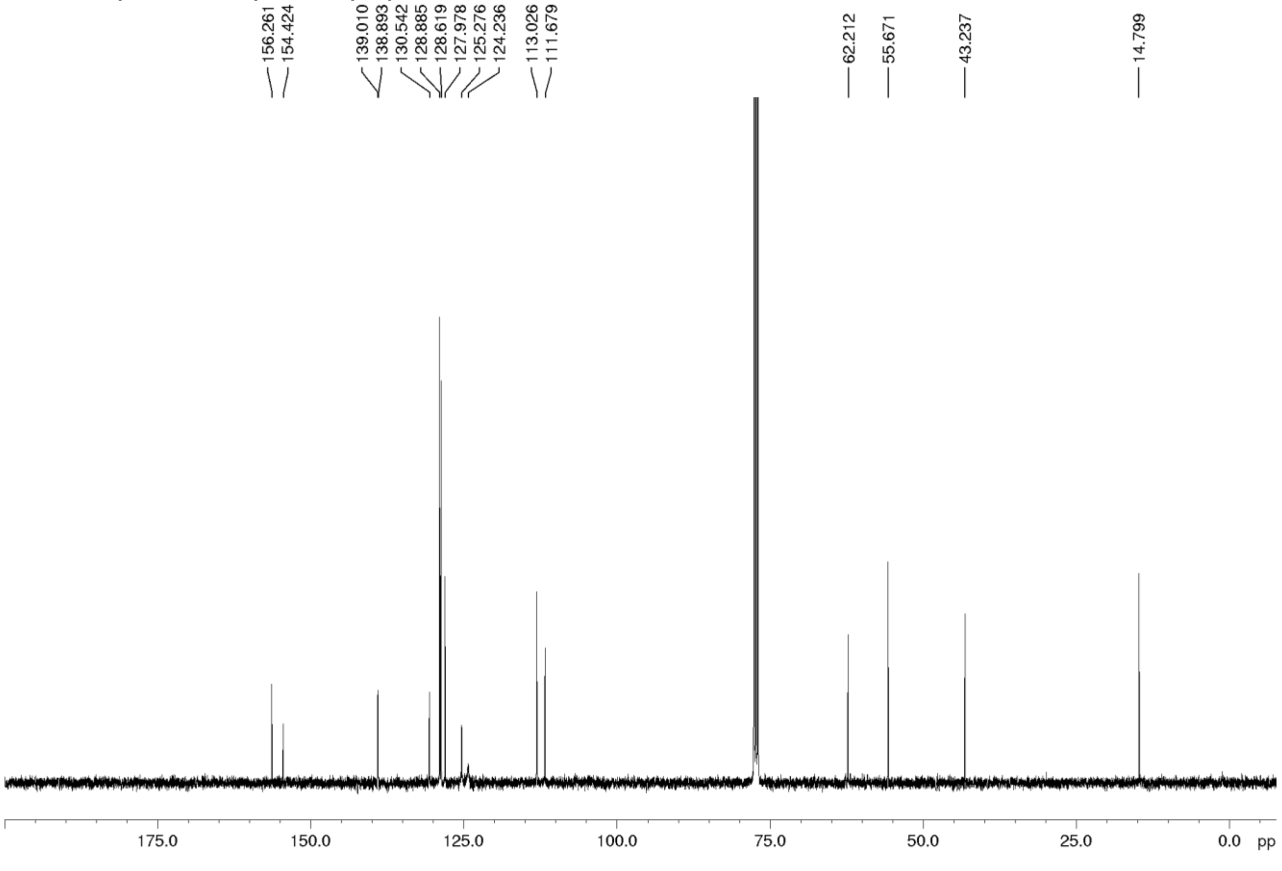

^{1}H NMR (400.13 MHz), $CDCl_3$ (2e)

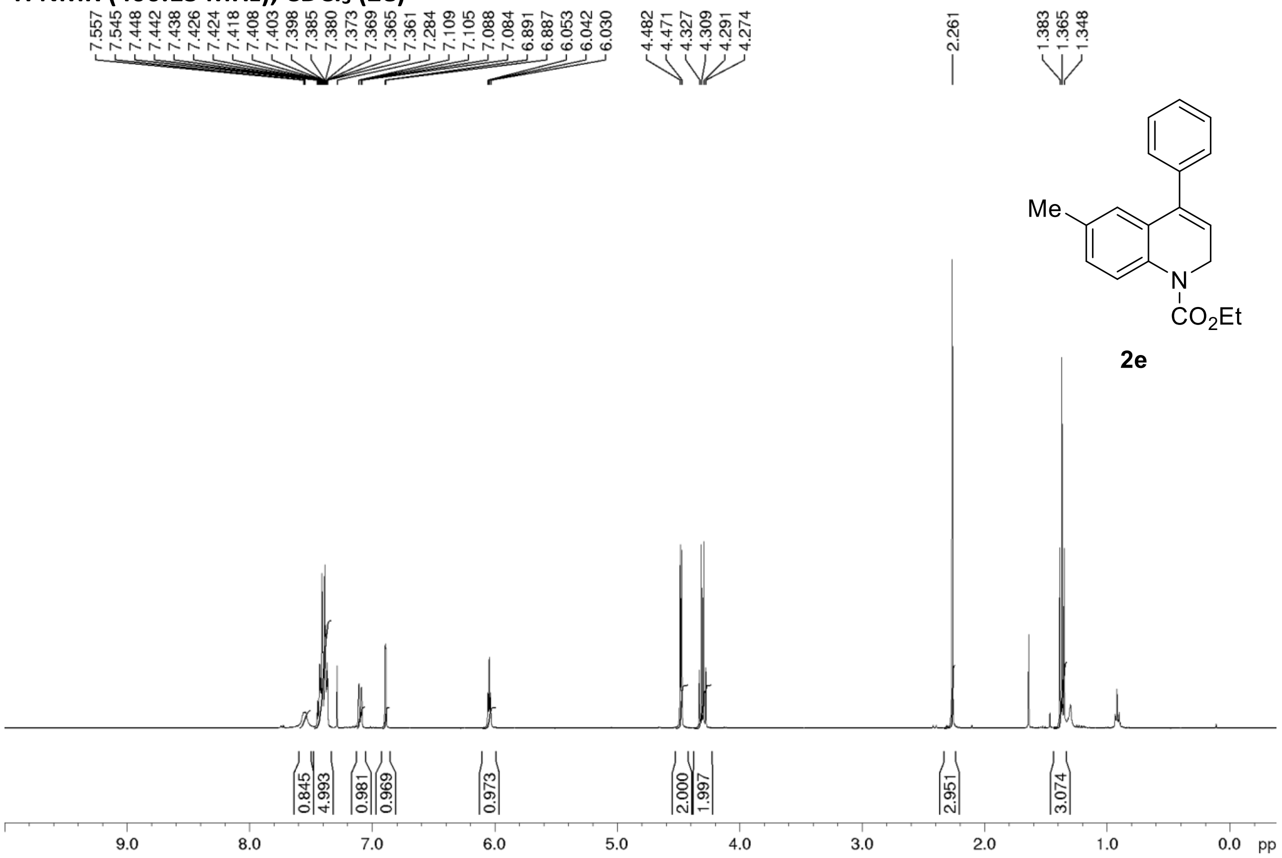

^{13}C NMR (100.6 MHz), $CDCl_3$ (2e)

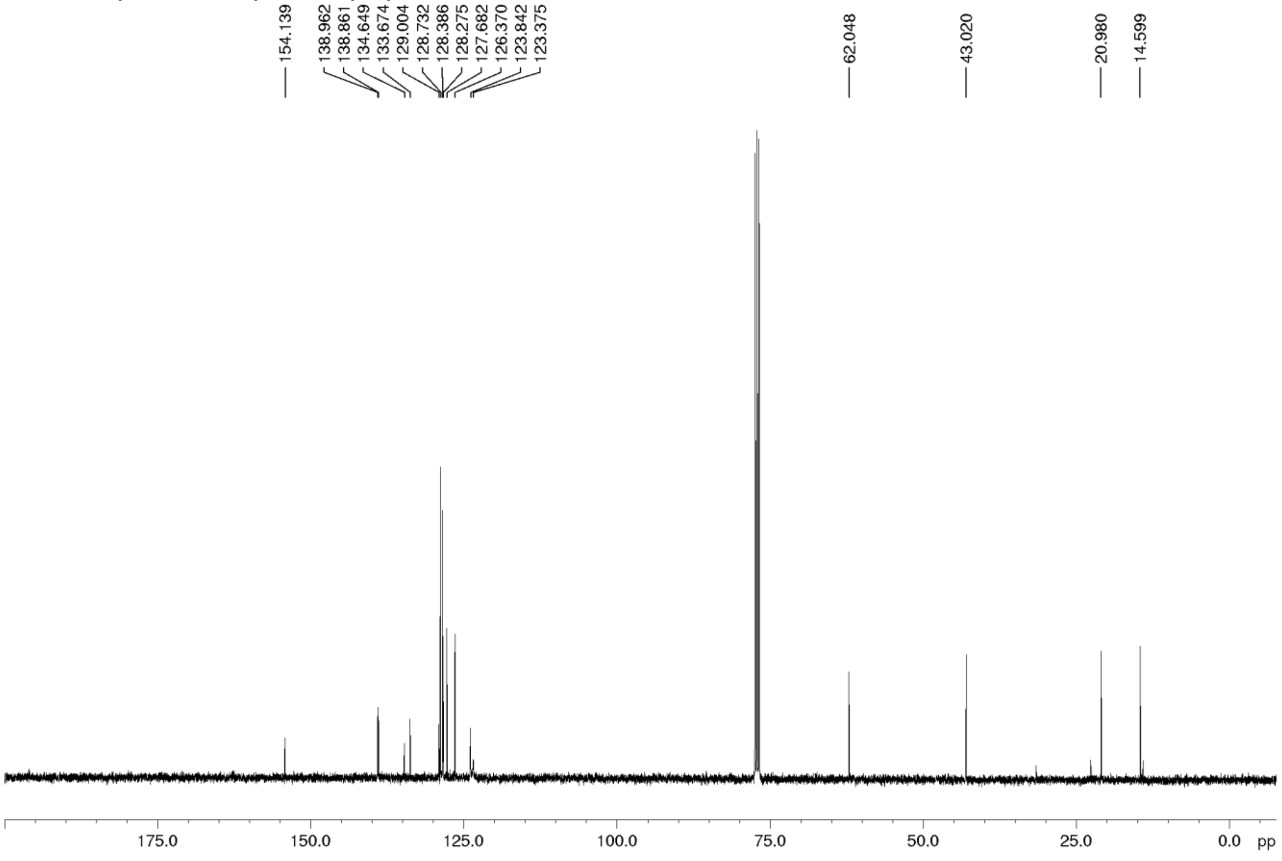

^1H NMR (400.13 MHz), $CDCl_3$ (2f)

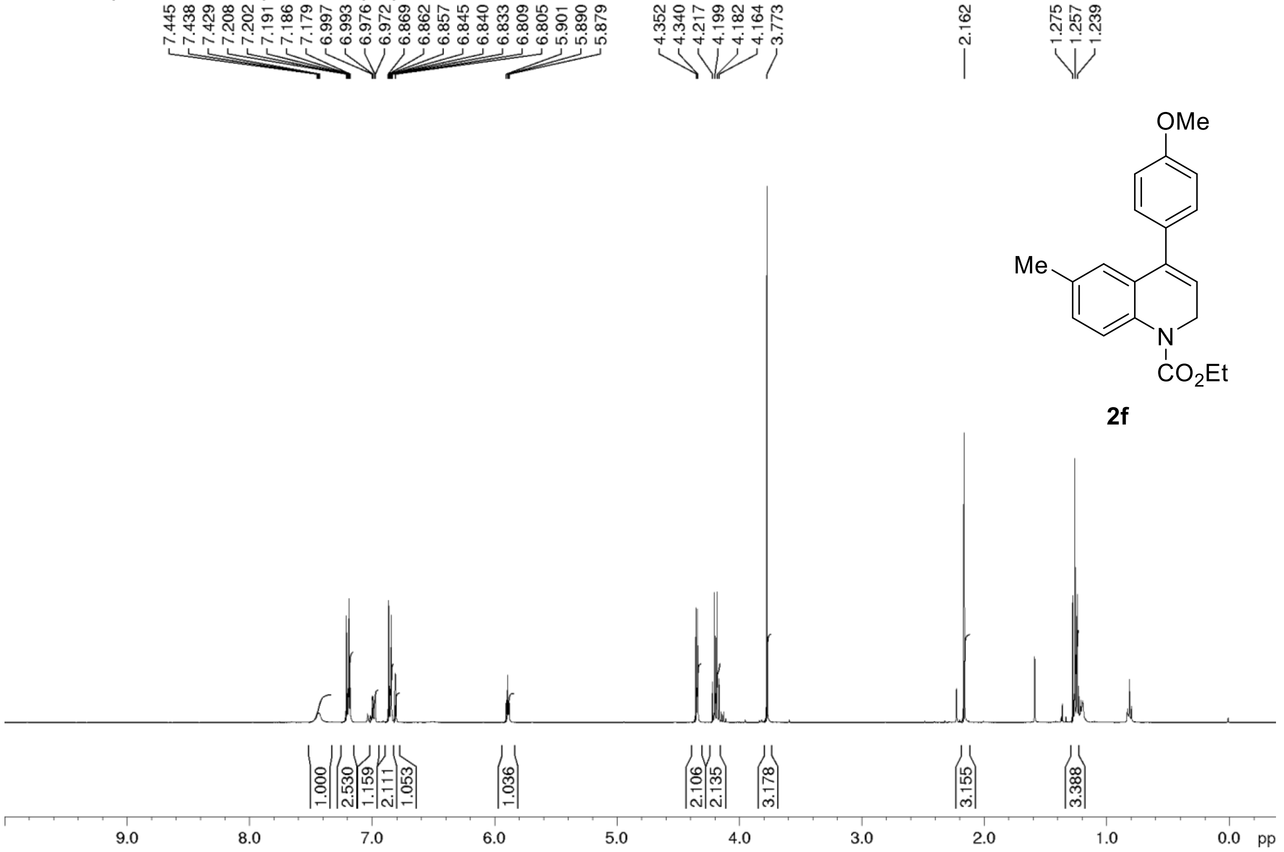

^{13}C NMR (100.6 MHz), $CDCl_3$ (2f)

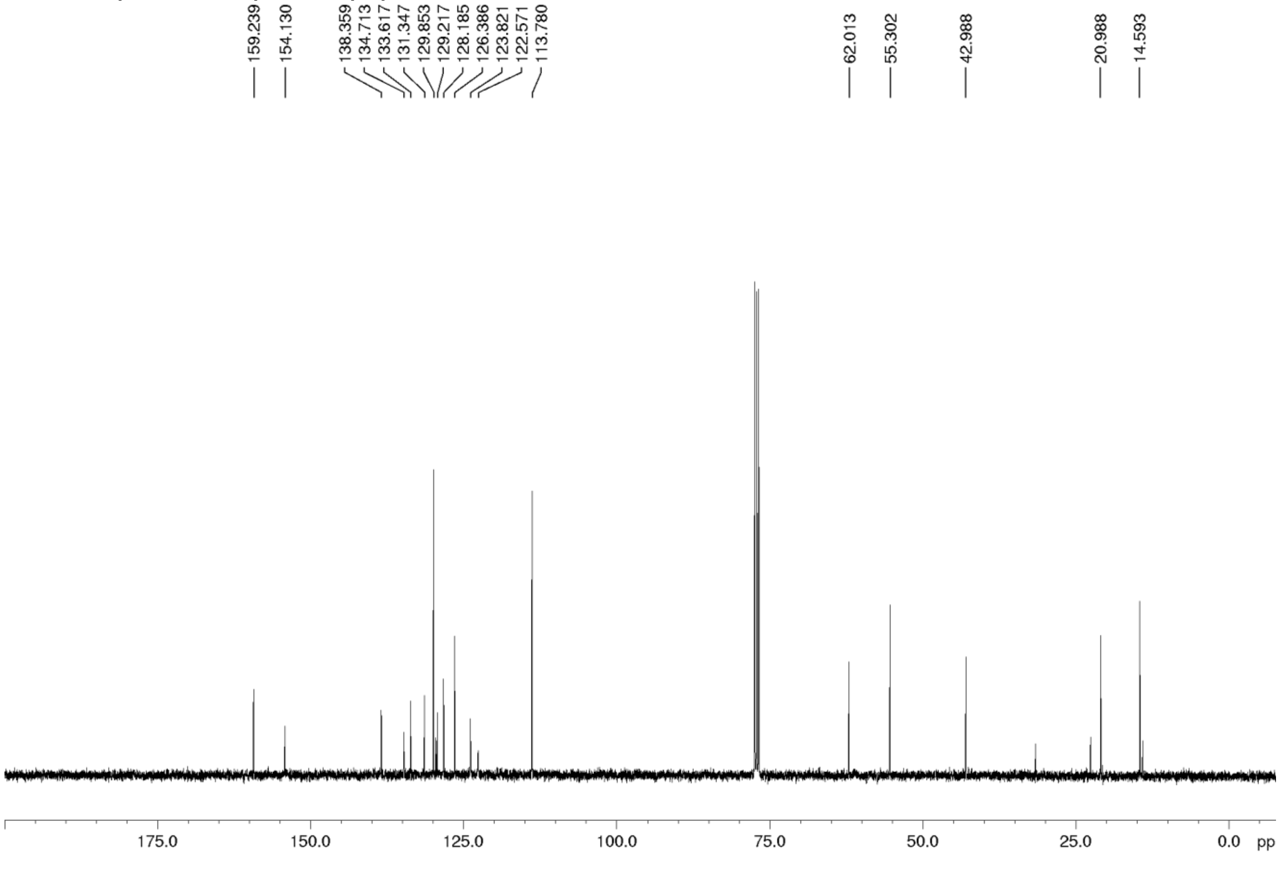

^{1}H NMR (400.13 MHz), $CDCl_3$ (2g)

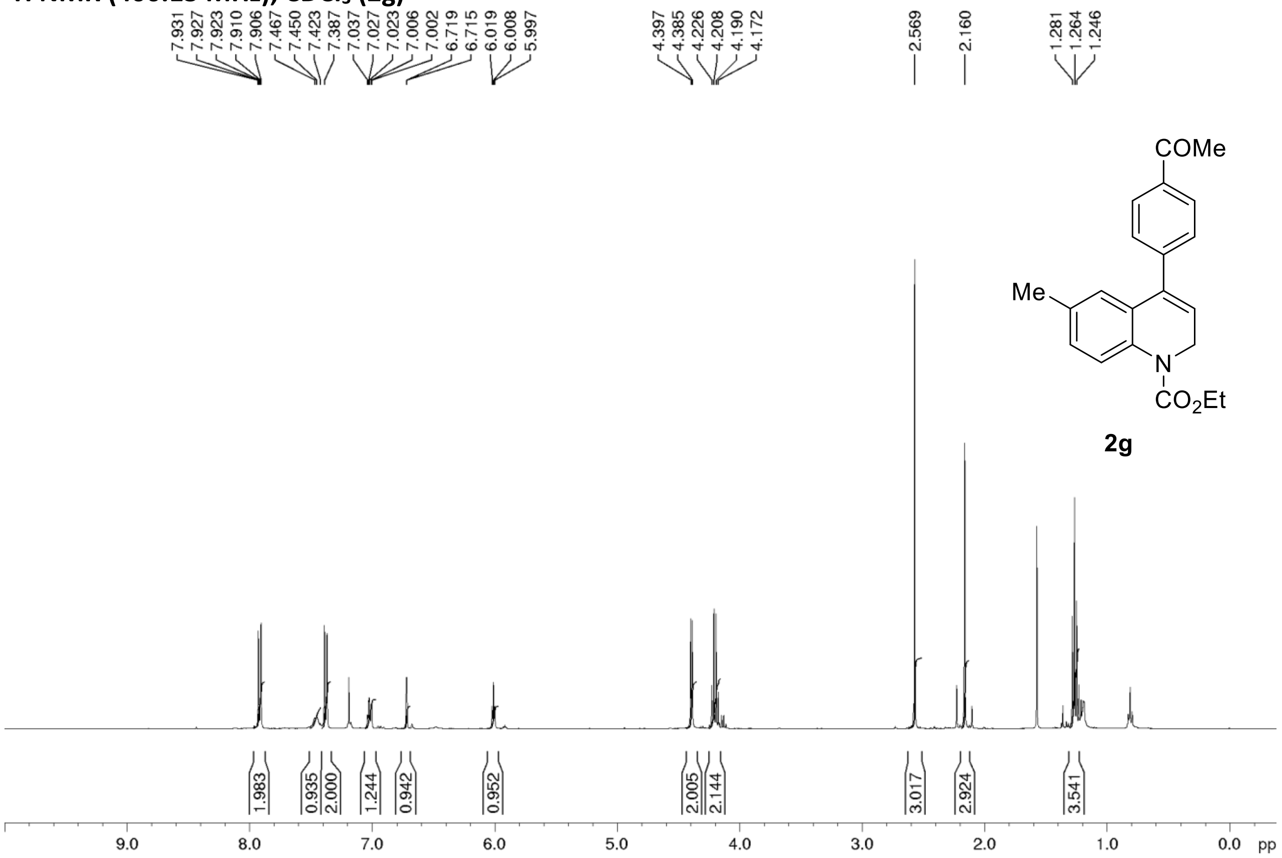

^{13}C NMR (100.6 MHz), $CDCl_3$ (2g)

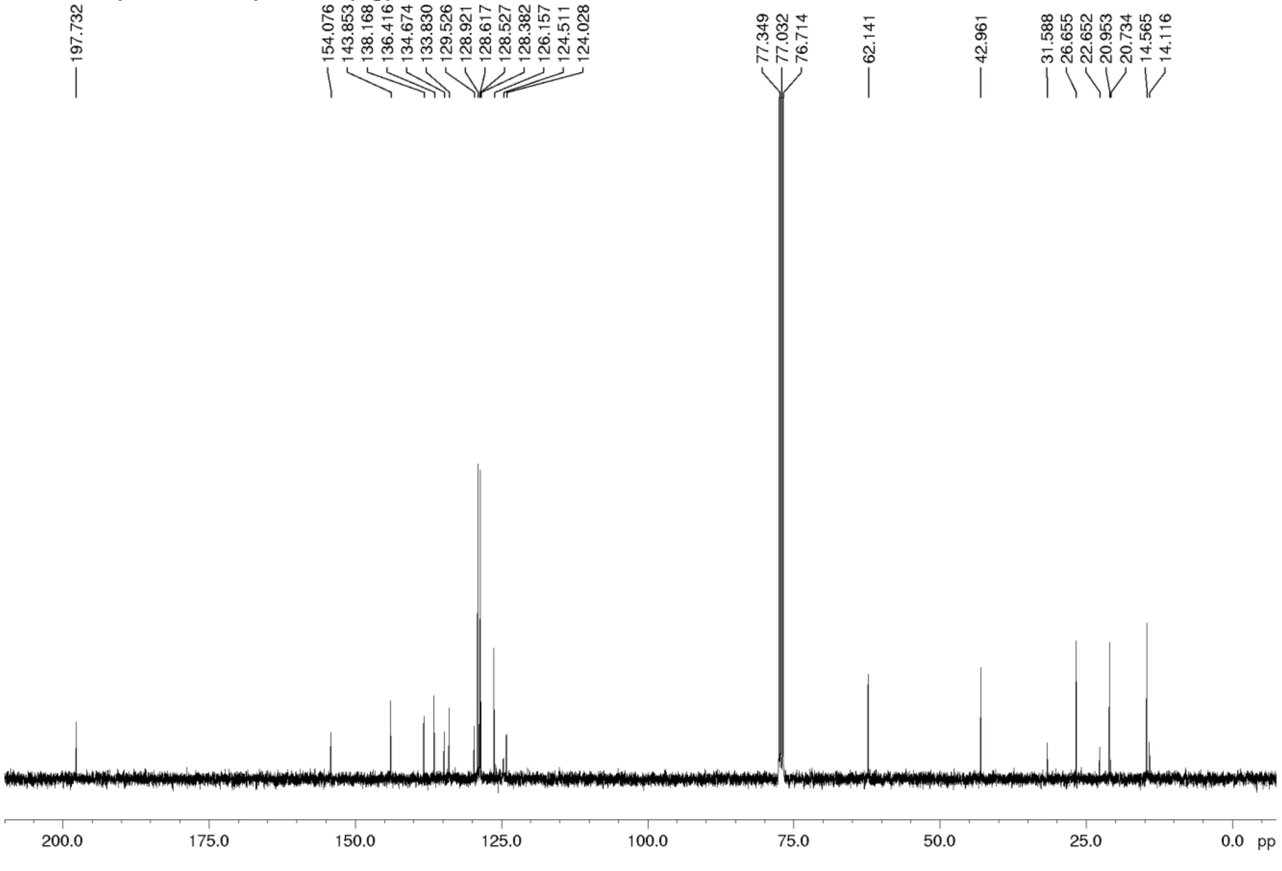

^{1}H NMR (400.13 MHz), $CDCl_3$ (2h)

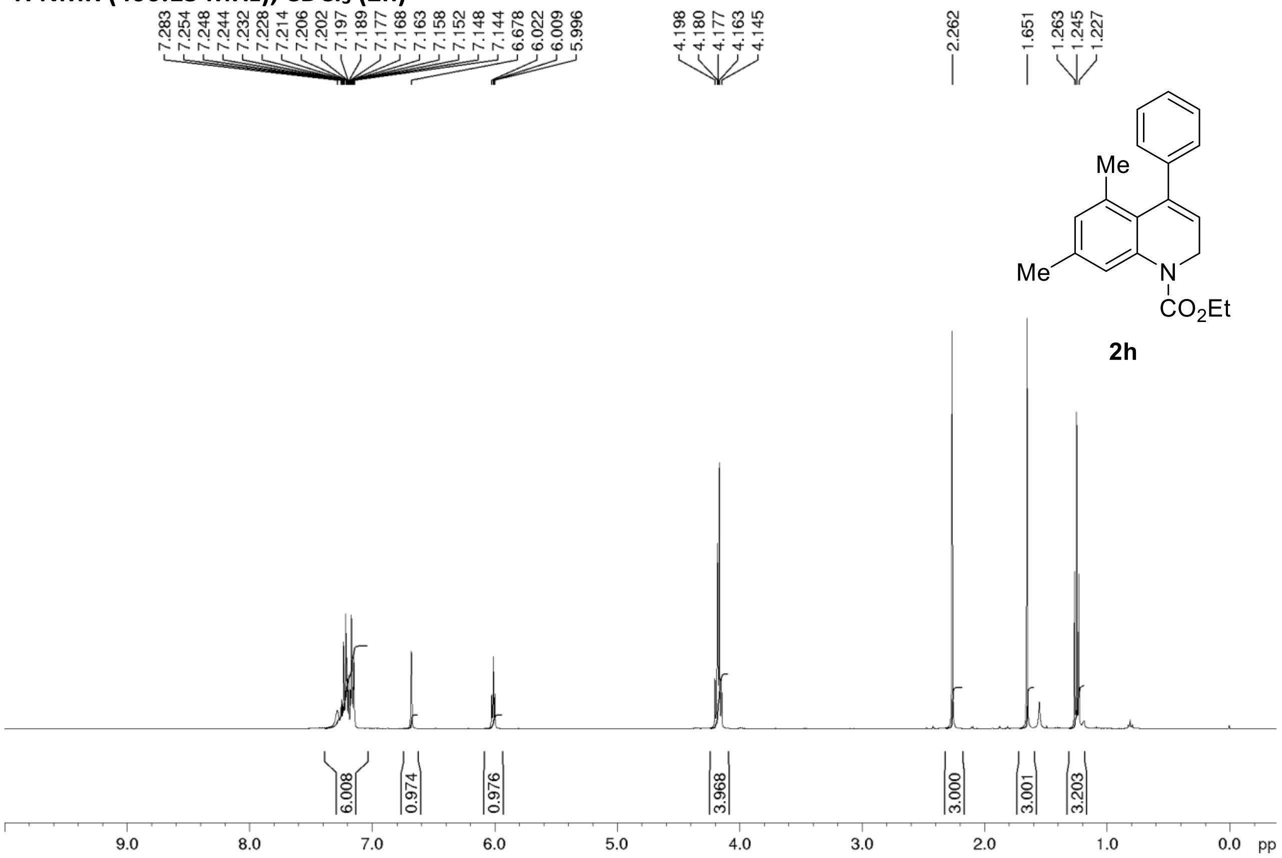

2h

^{13}C NMR (100.6 MHz), $CDCl_3$ (2h)

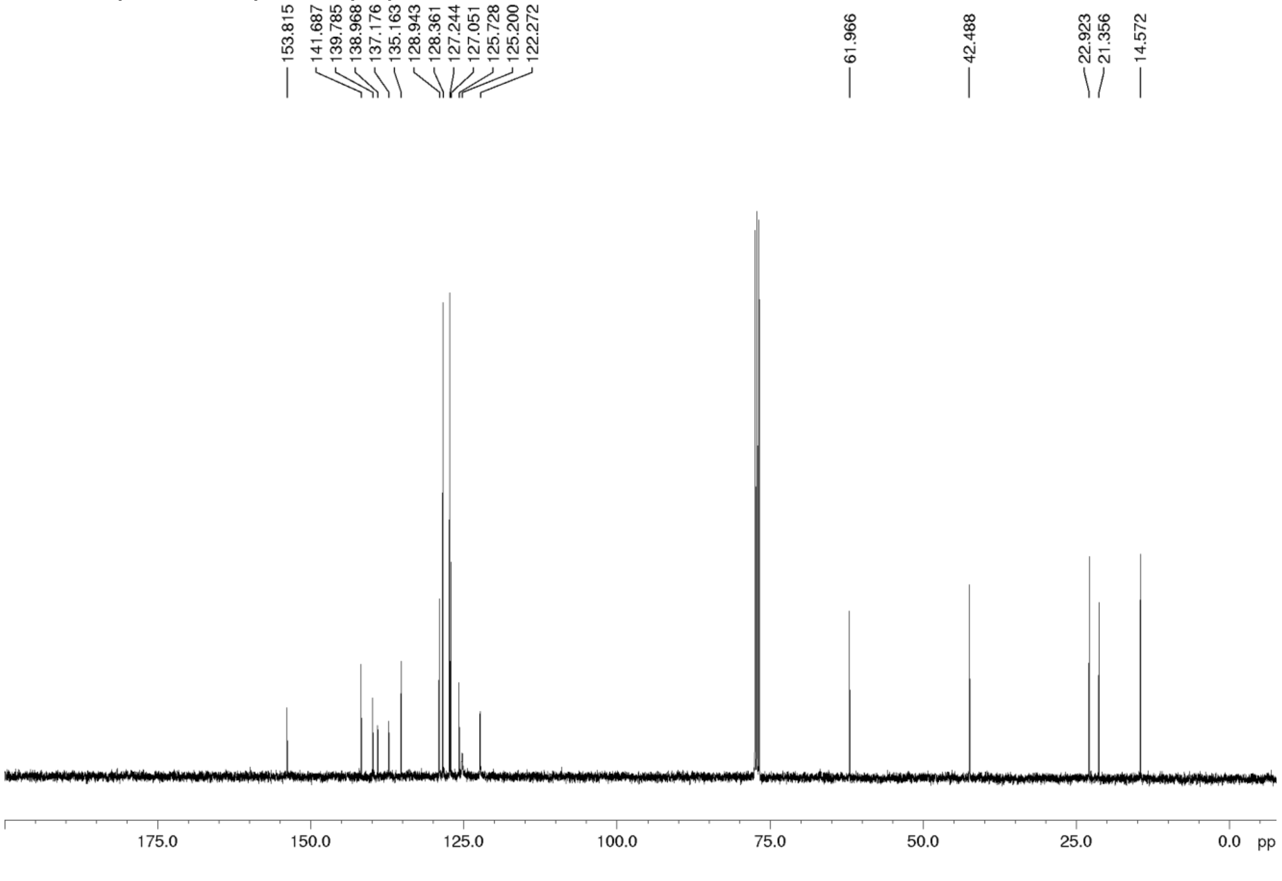

^{1}H NMR (400.13 MHz), $CDCl_3$ (2i)

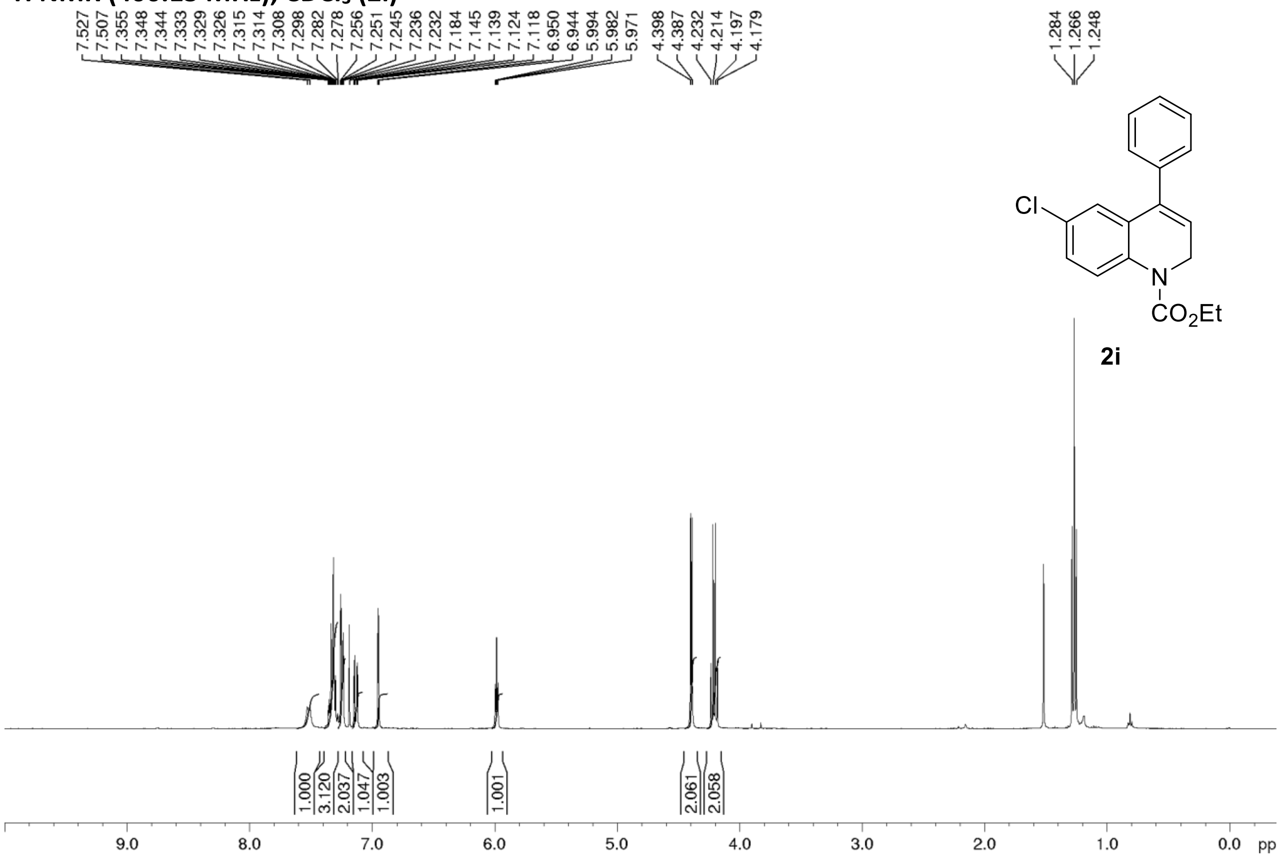

^{13}C NMR (100.6 MHz), $CDCl_3$ (2i)

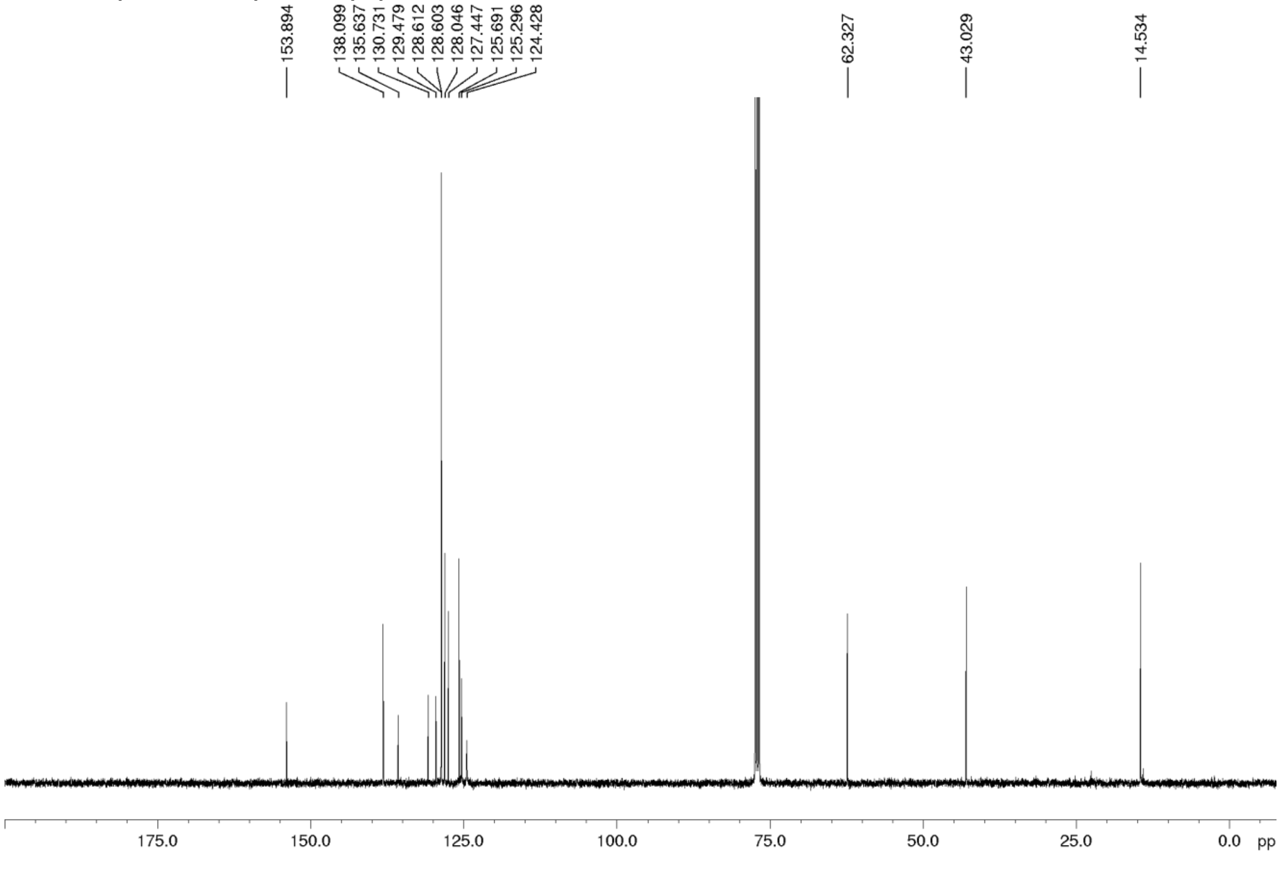

^{1}H NMR (400.13 MHz), $CDCl_3$ (2j)

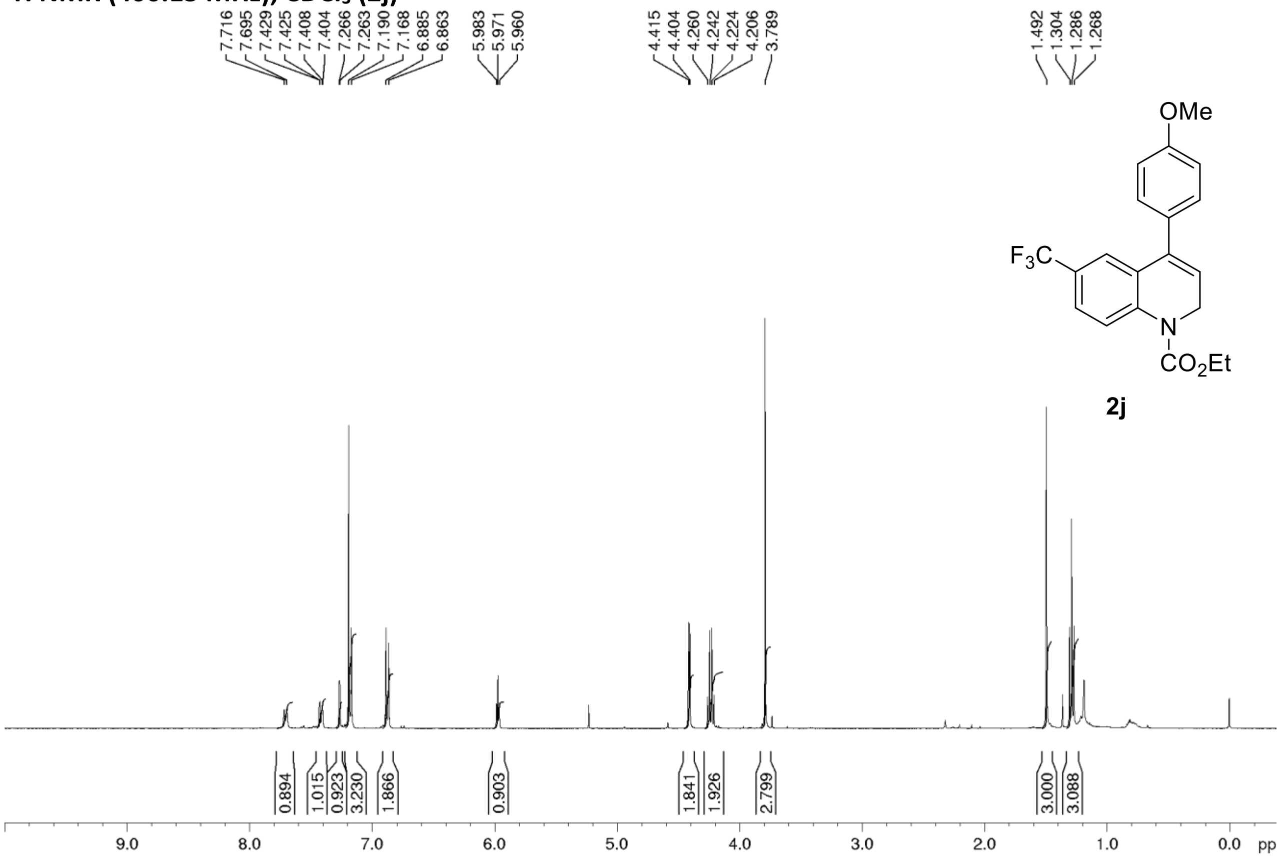

^{13}C NMR (100.6 MHz), $CDCl_3$ (2j)

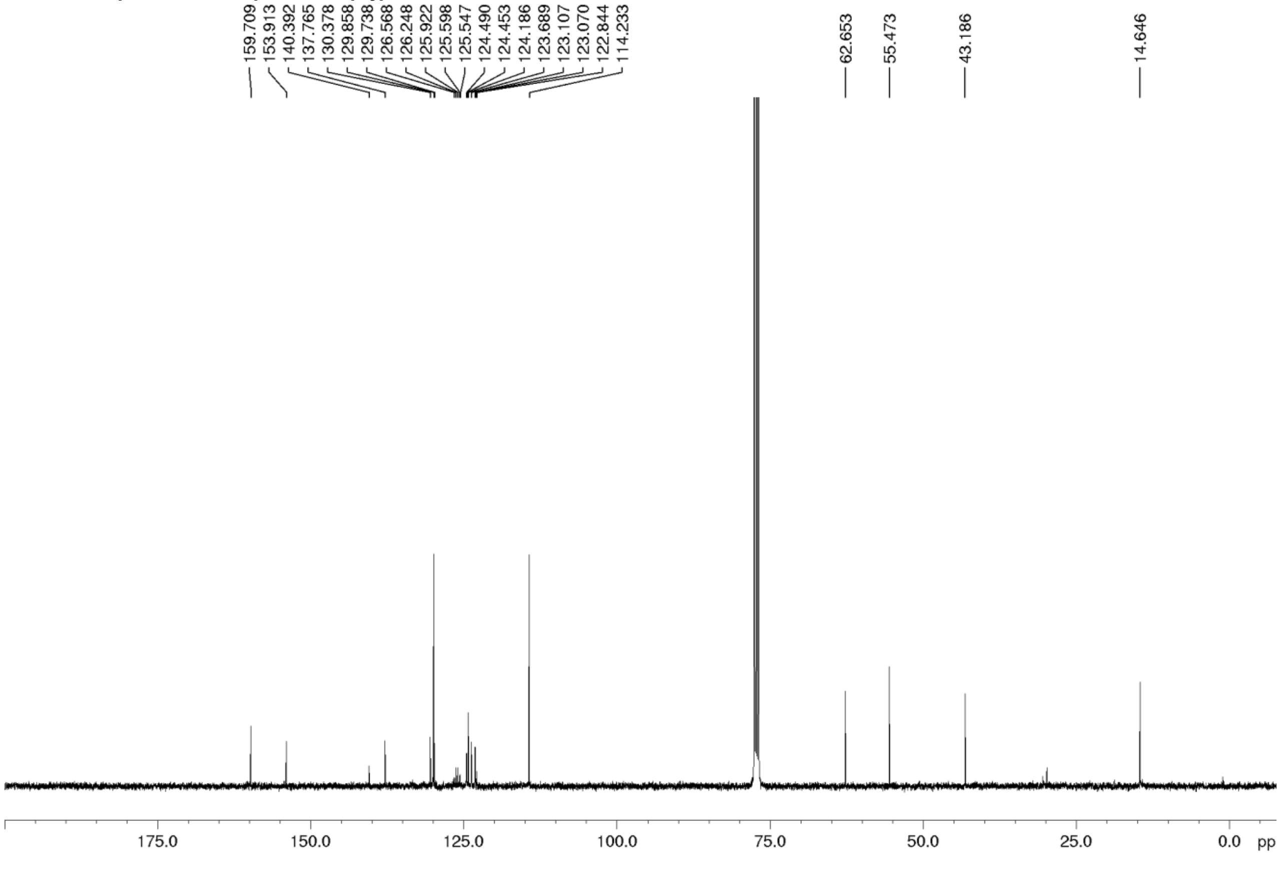

^{19}F NMR (376.5 MHz), $CDCl_3$ (2j)

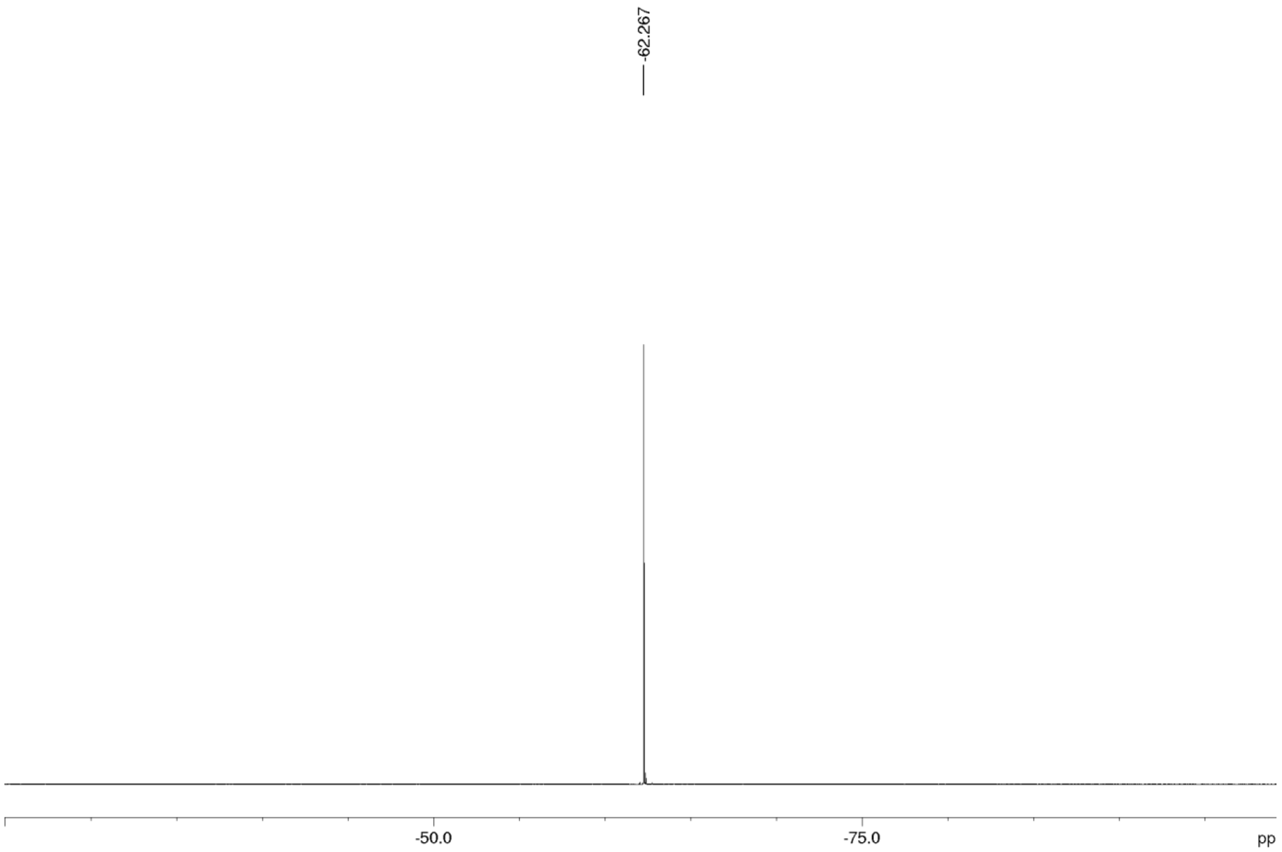

^{1}H NMR (400.13 MHz), CDCl$_3$ (2k)

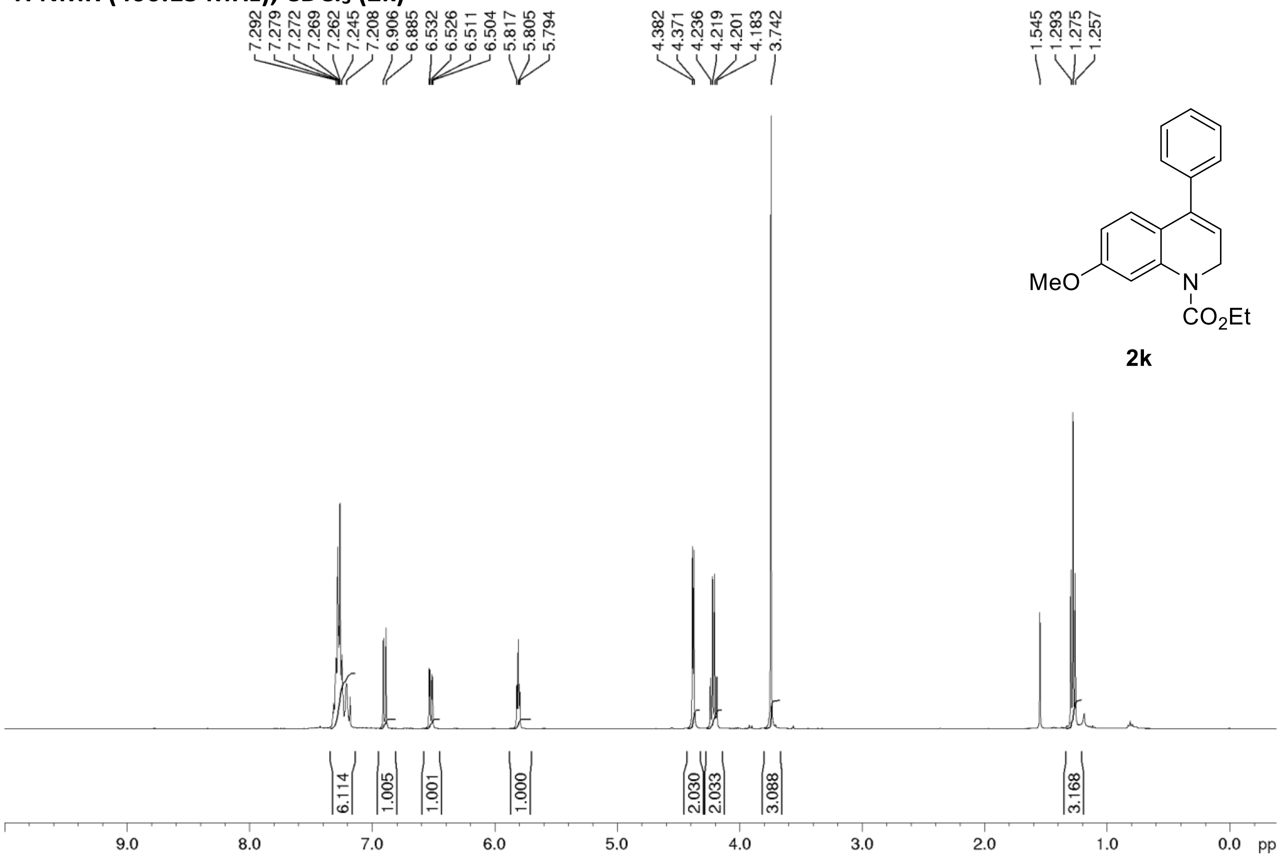

^{13}C NMR (100.6 MHz), CDCl$_3$ (2k)

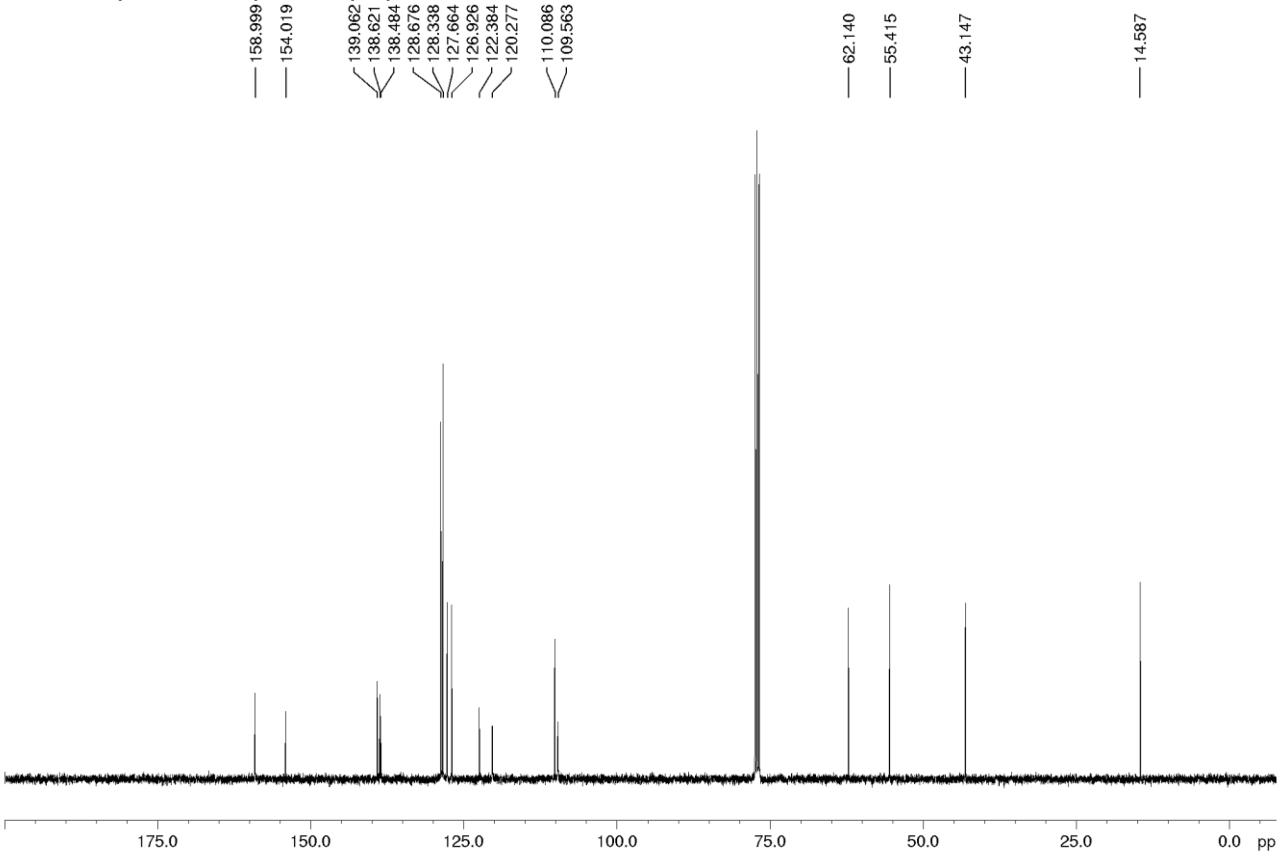

^{1}H NMR (400.13 MHz), $CDCl_3$ (2'k)

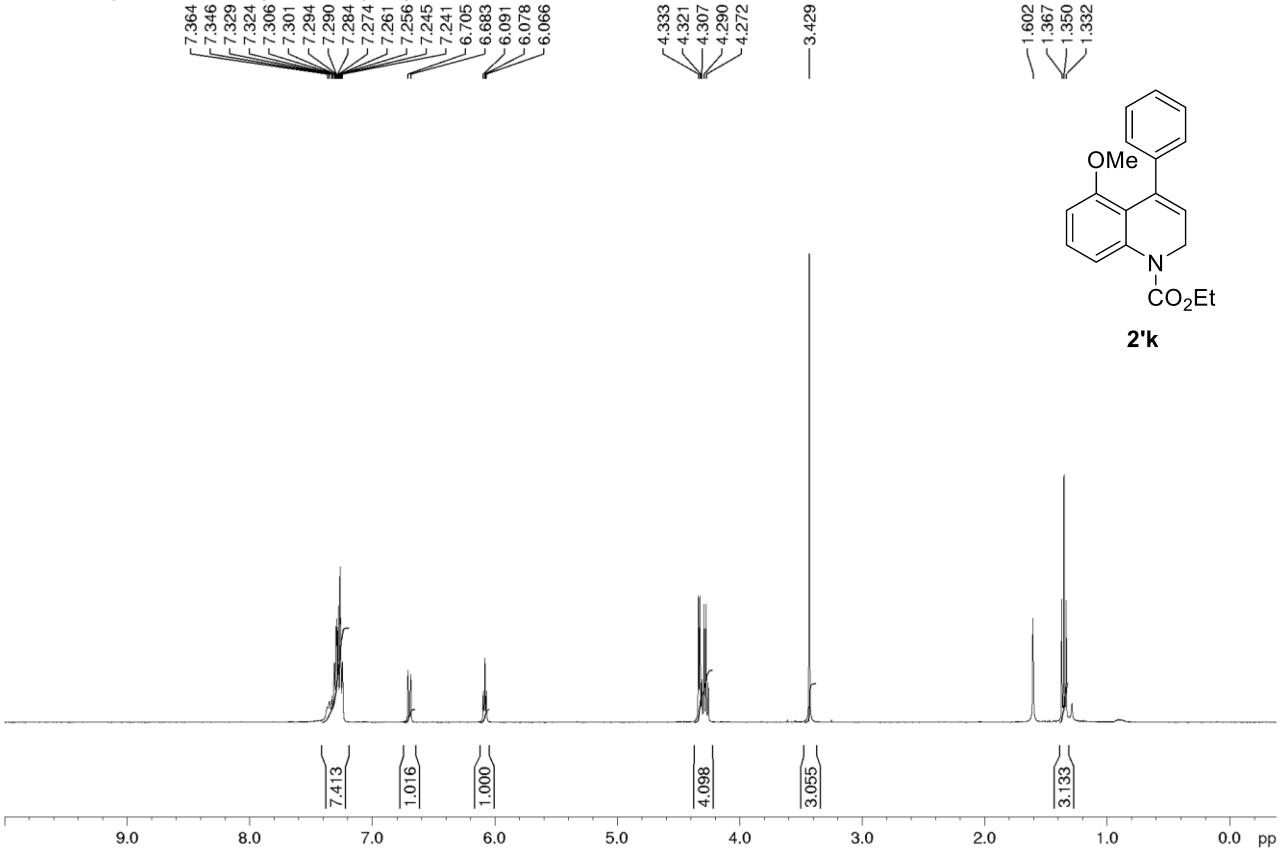

^{13}C NMR (100.6 MHz), $CDCl_3$ (2'k)

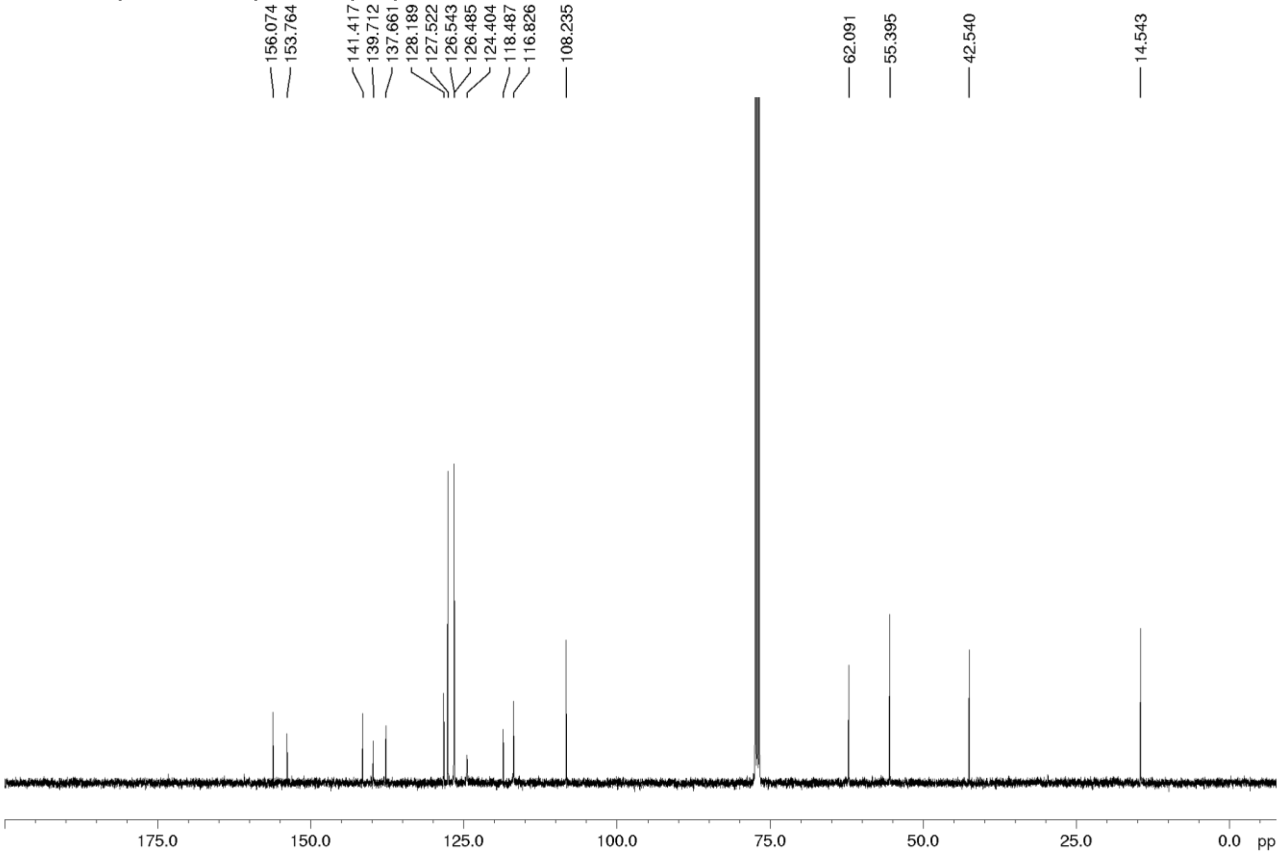

^{1}H NMR (400.13 MHz), $CDCl_3$ (2l + 2'l)

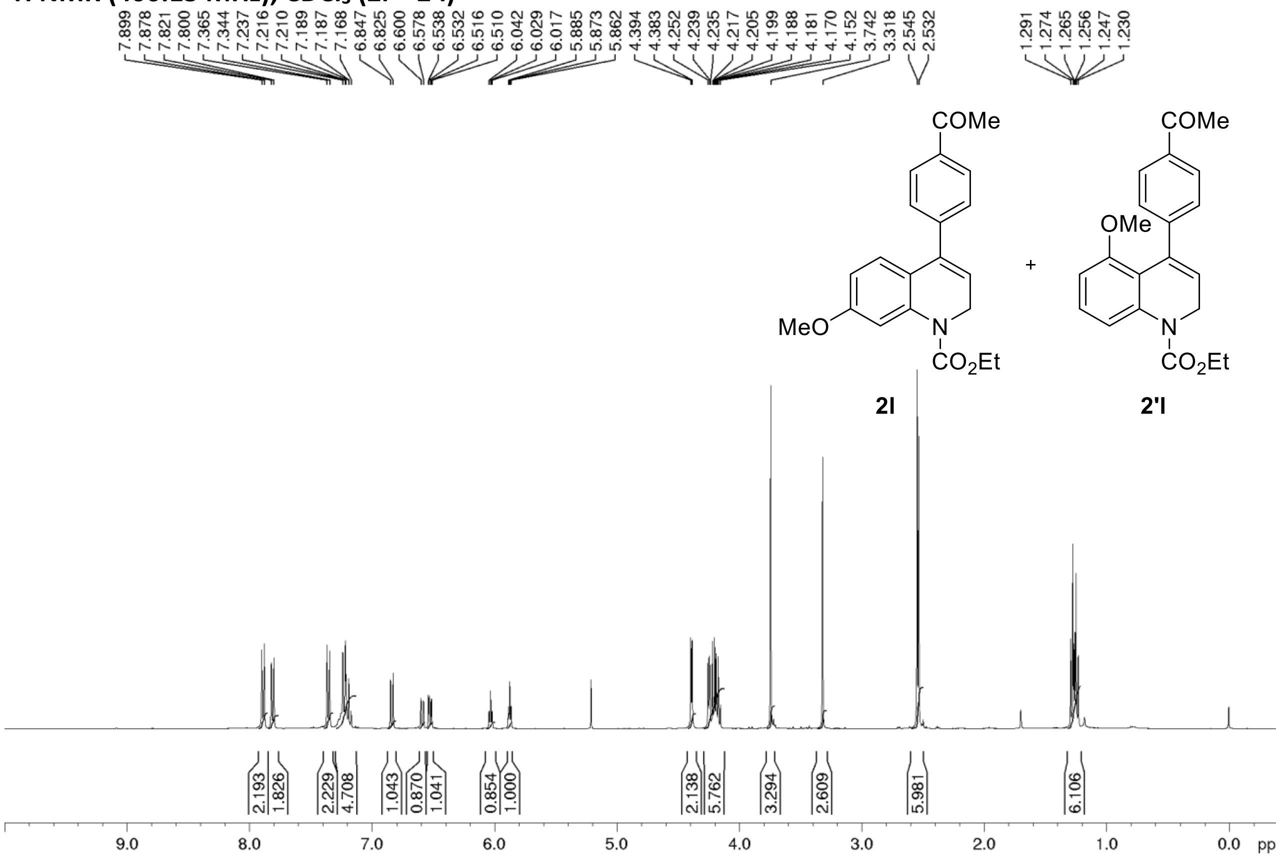

^{13}C NMR (100.6 MHz), $CDCl_3$ (2l + 2'l)

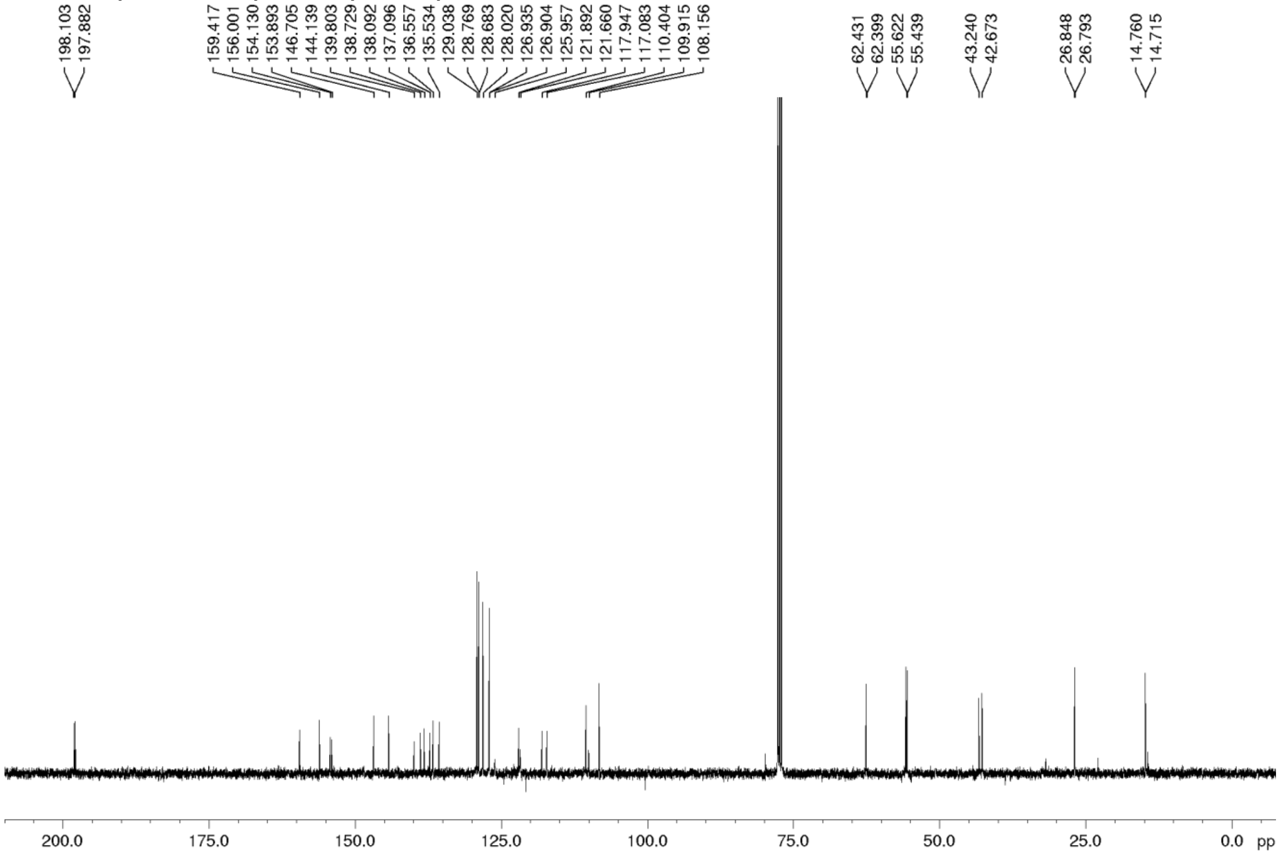

^{1}H NMR (400.13 MHz), $CDCl_3$ (2m)

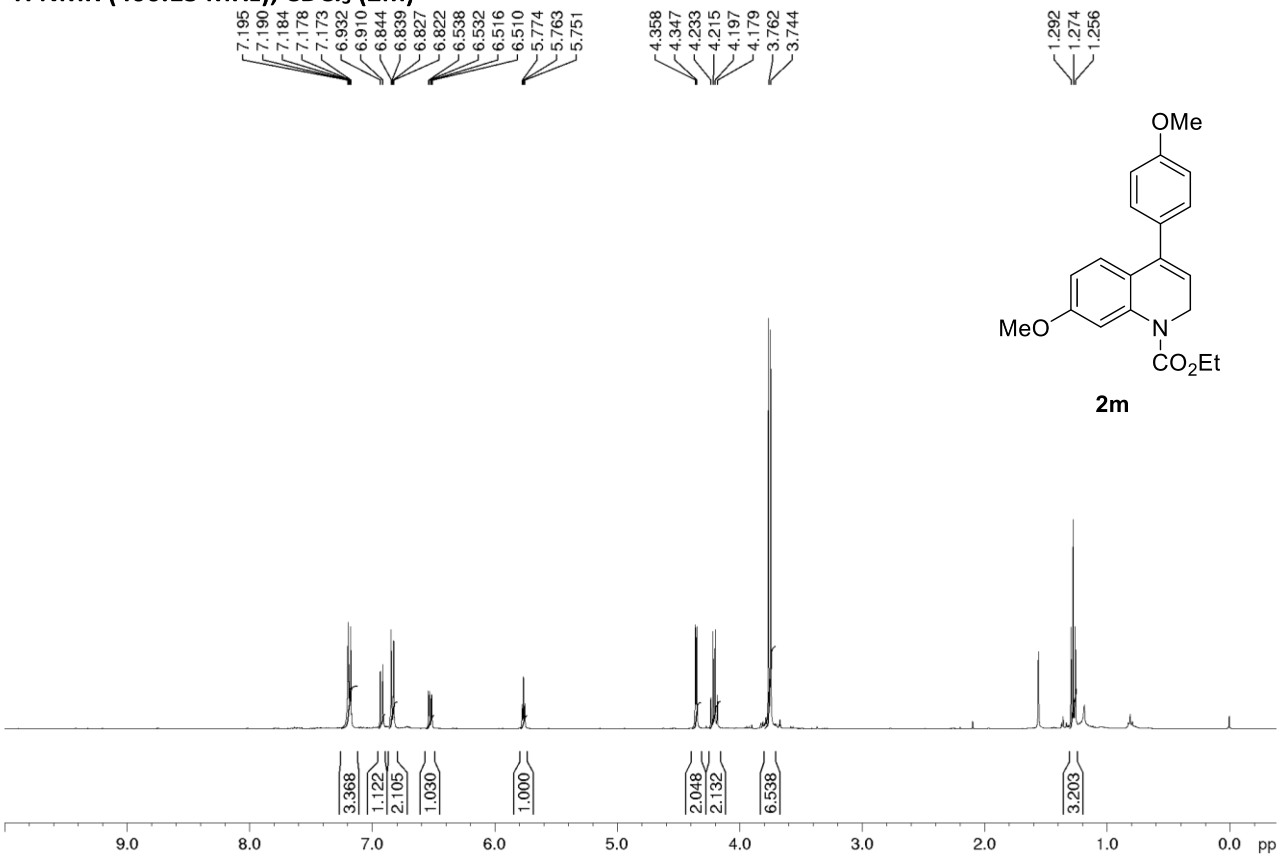

2m

^{13}C NMR (100.6 MHz), $CDCl_3$ (2m)

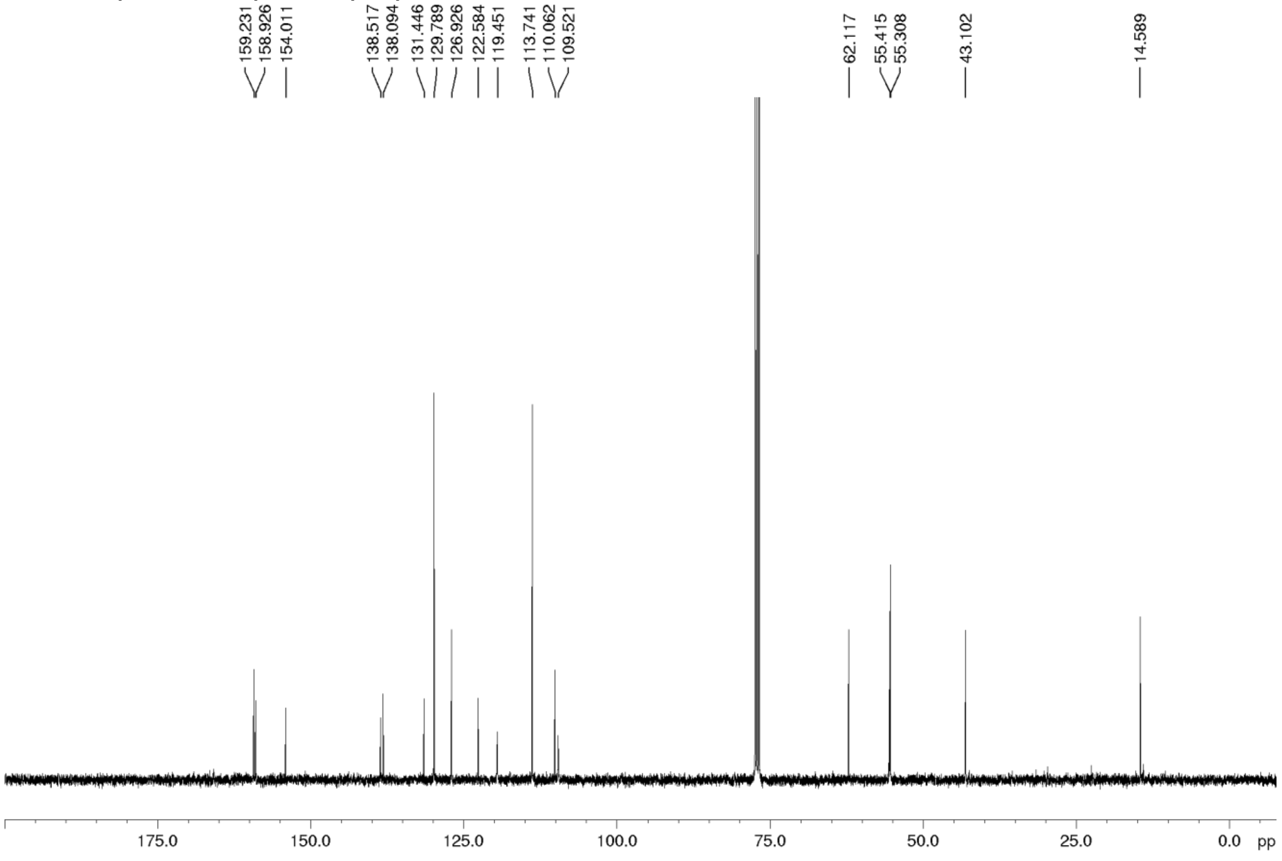

^{1}H NMR (400.13 MHz), $CDCl_3$ (2’m)

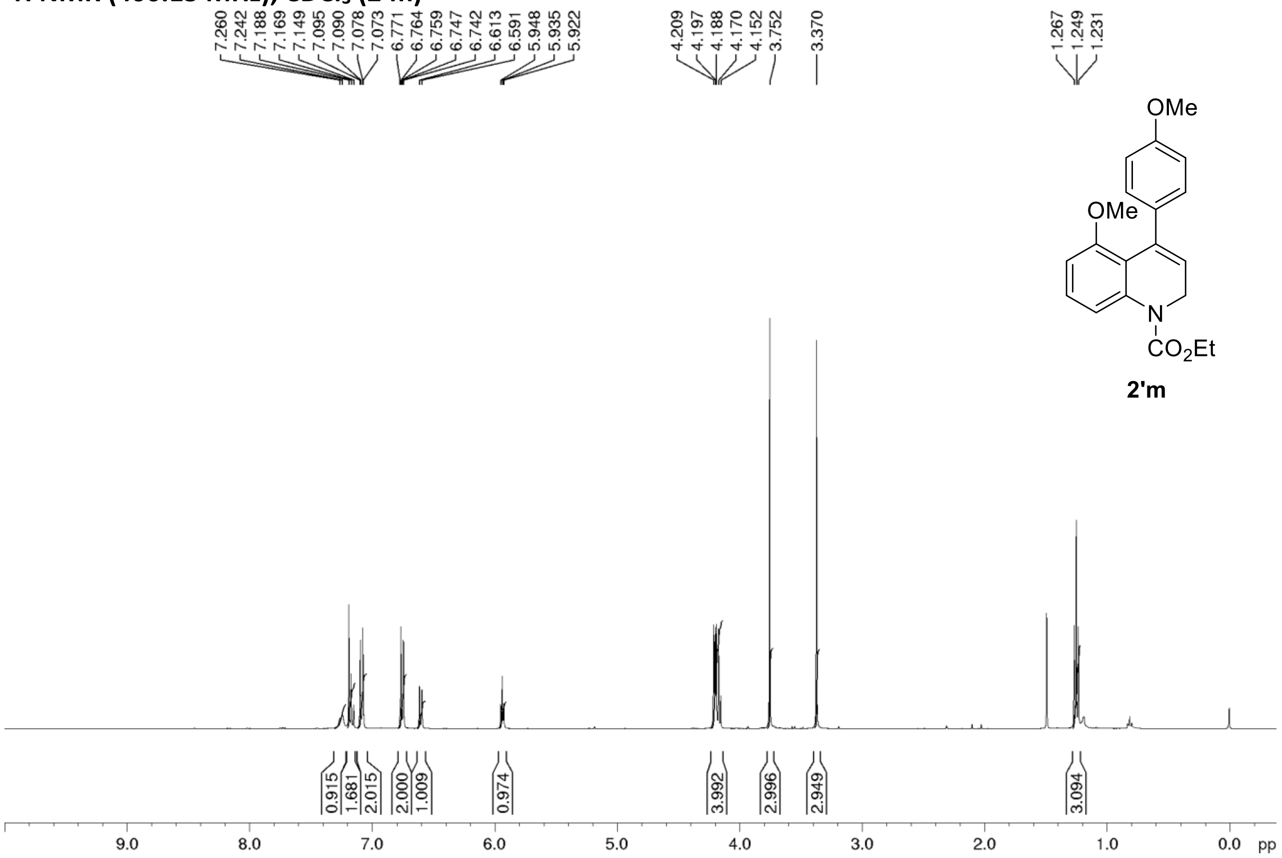

^{13}C NMR (100.6 MHz), $CDCl_3$ (2’m)

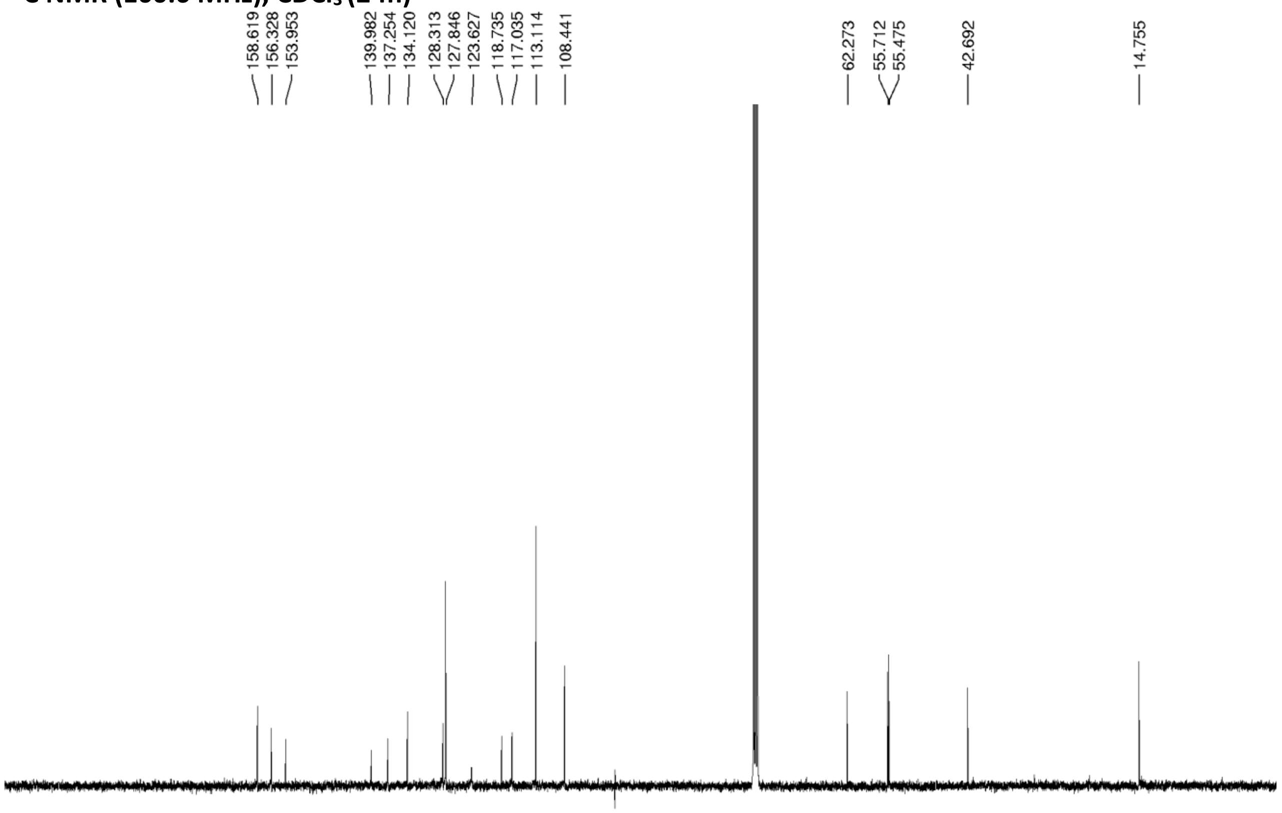

^{1}H NMR (400.13 MHz), $CDCl_3$ (2n)

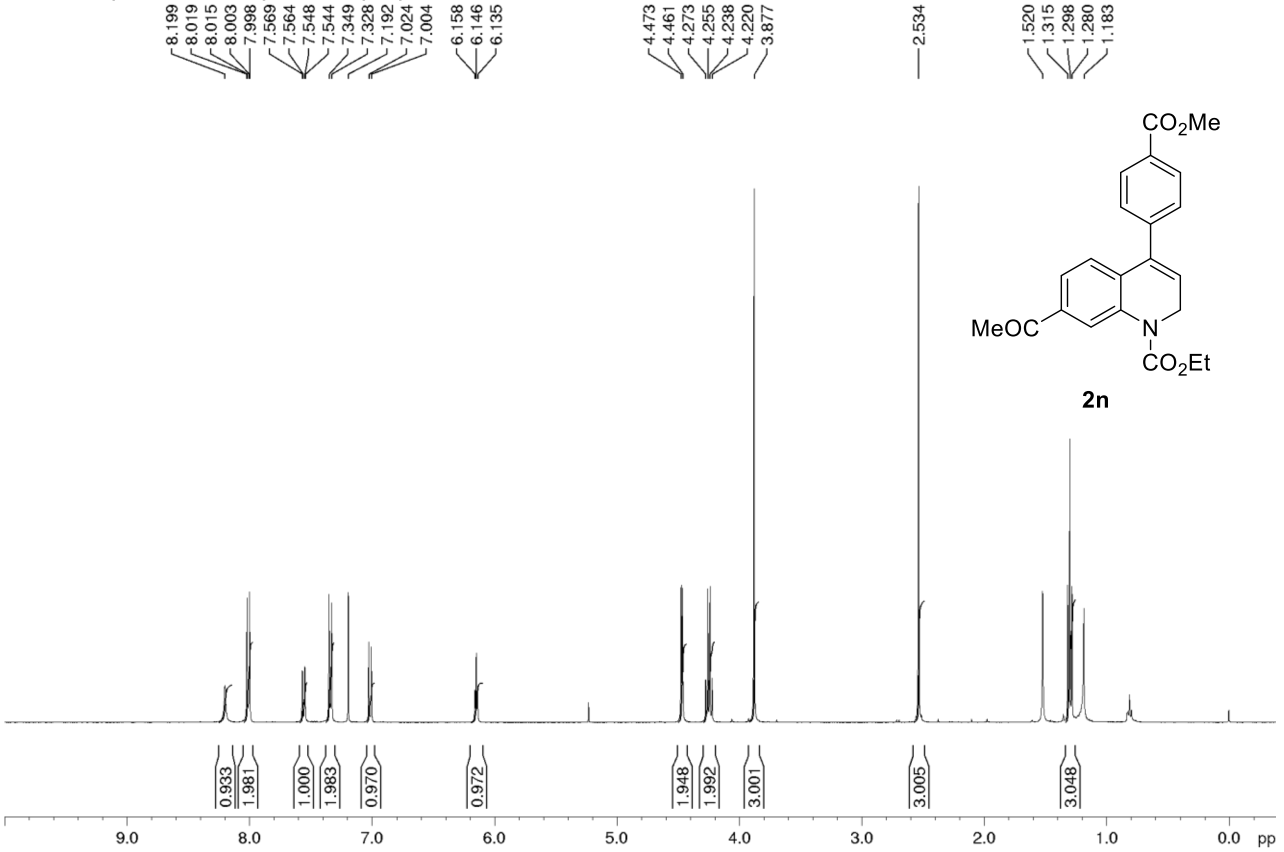

^{13}C NMR (100.6 MHz), $CDCl_3$ (2n)

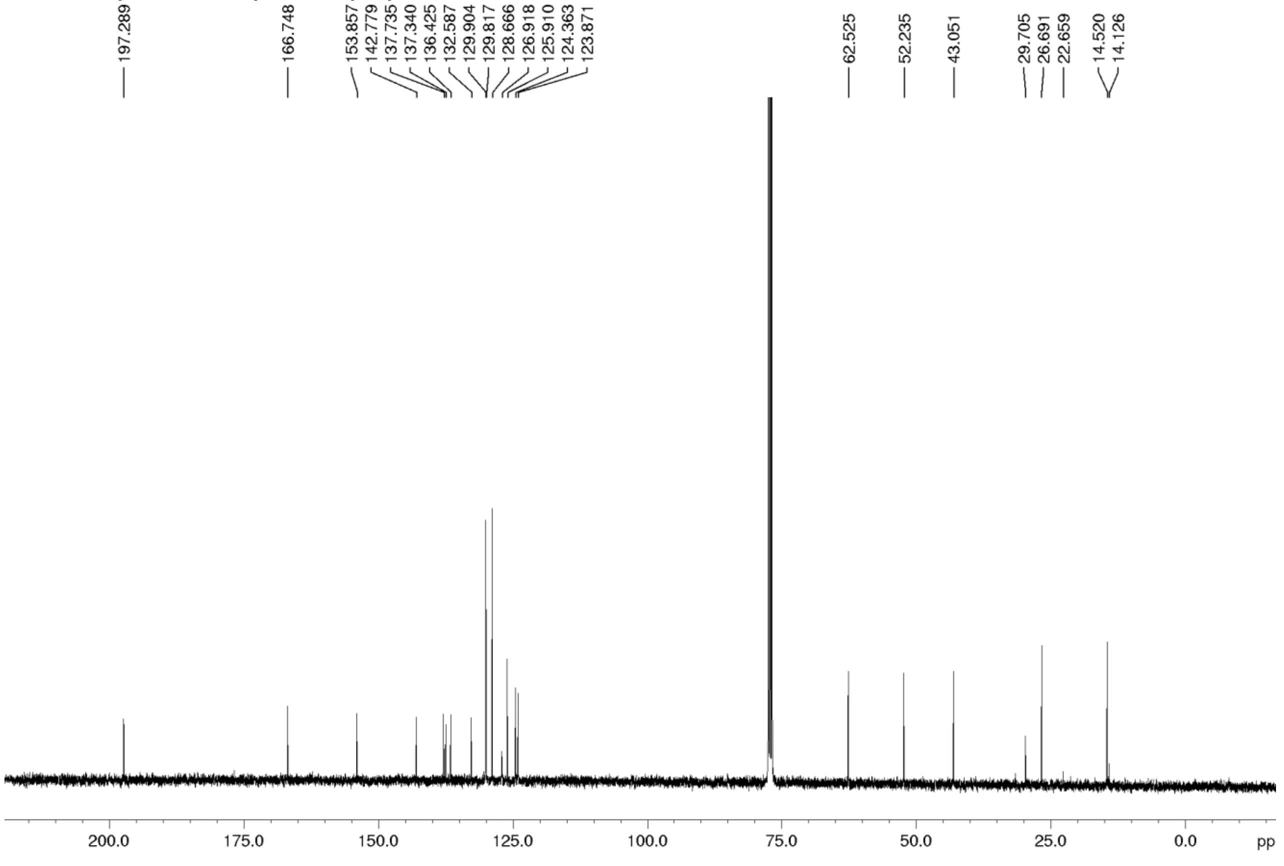

^{1}H NMR (400.13 MHz), $CDCl_3$ (2'n)

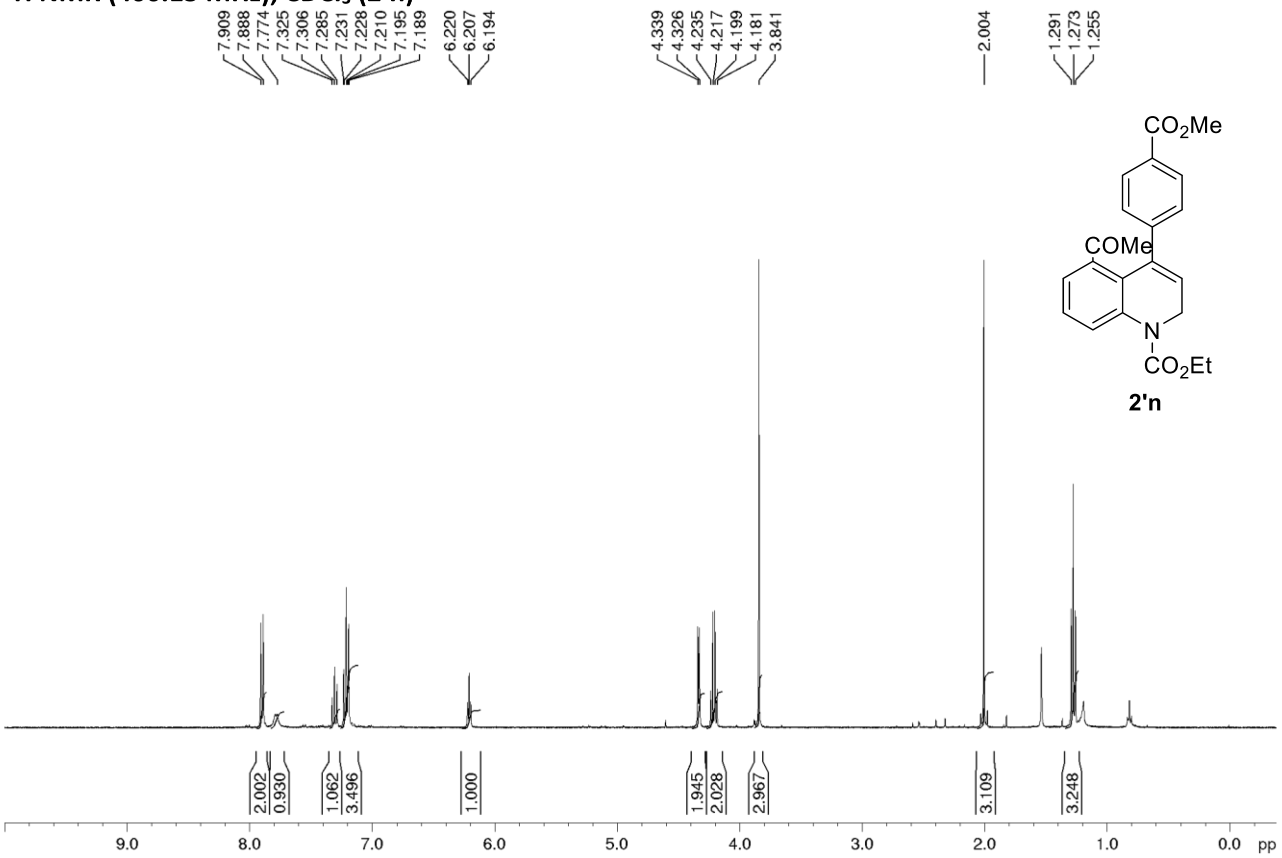

2'n

^{13}C NMR (100.6 MHz), $CDCl_3$ (2'n)

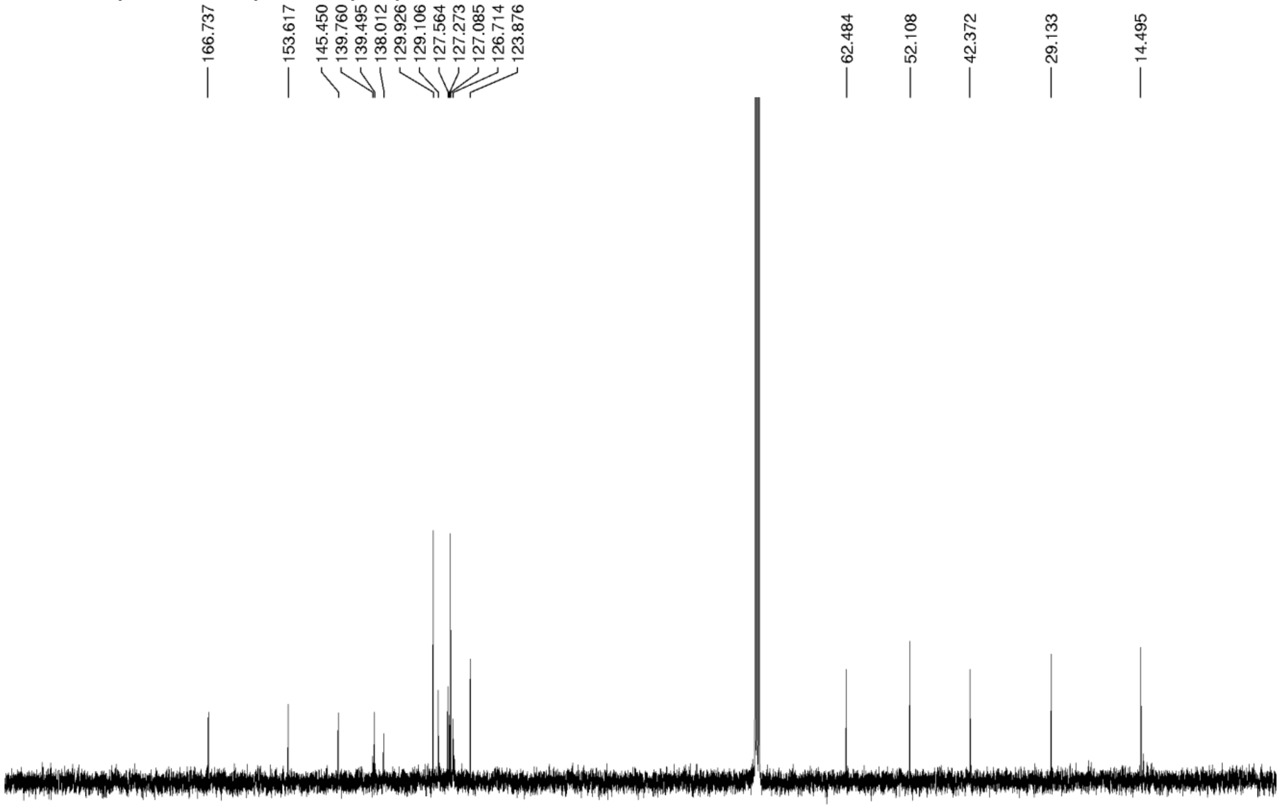

^{1}H NMR (400.13 MHz), $CDCl_3$ (2o)

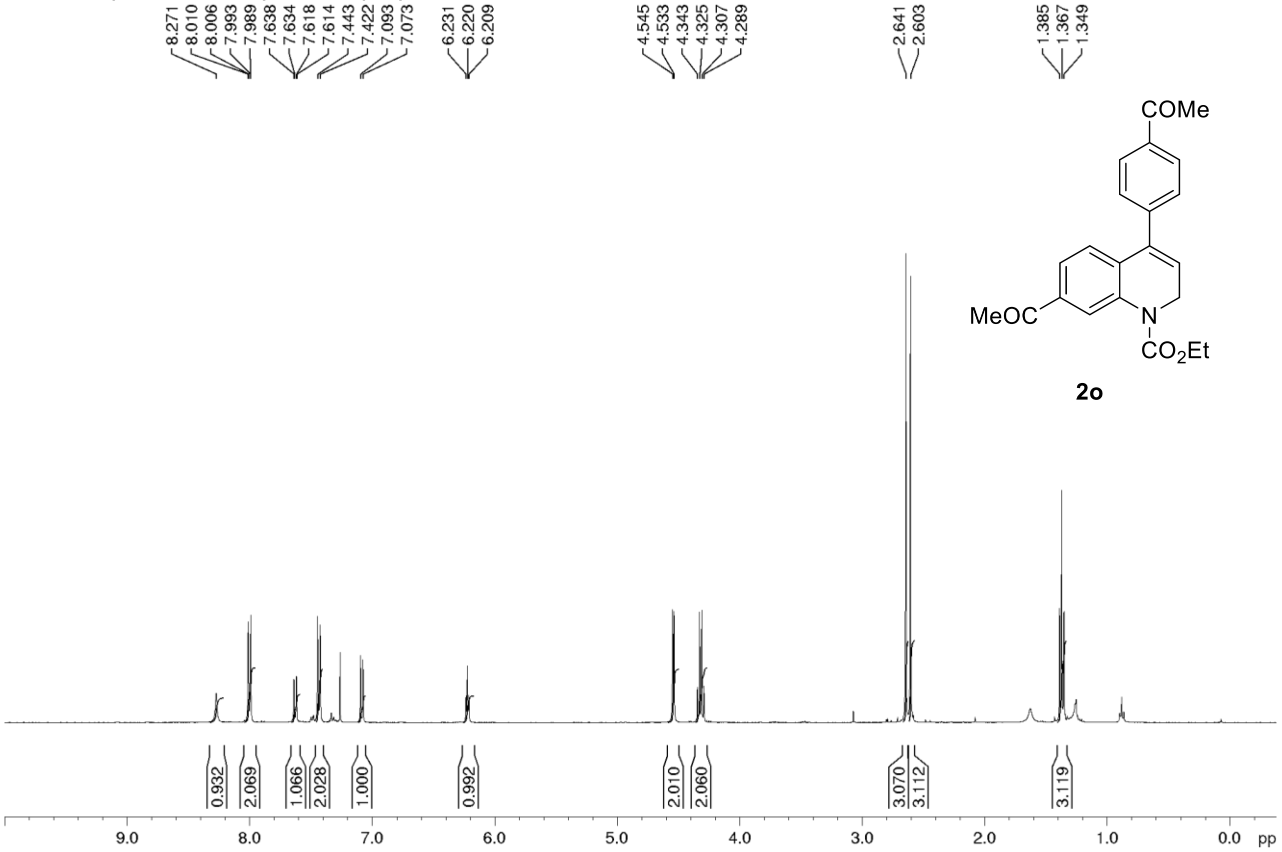

^{13}C NMR (100.6 MHz), $CDCl_3$ (2o)

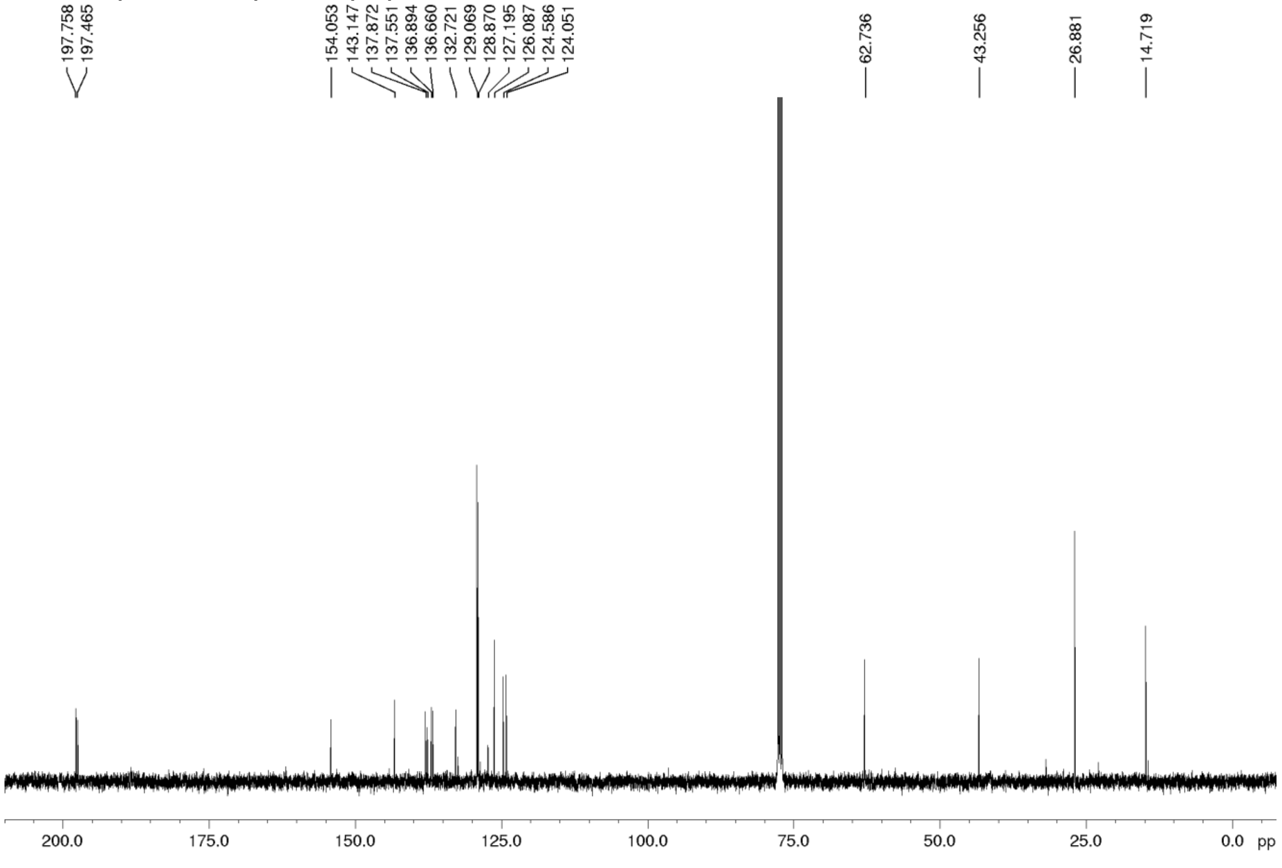

^{1}H NMR (400.13 MHz), $CDCl_3$ (2'o)

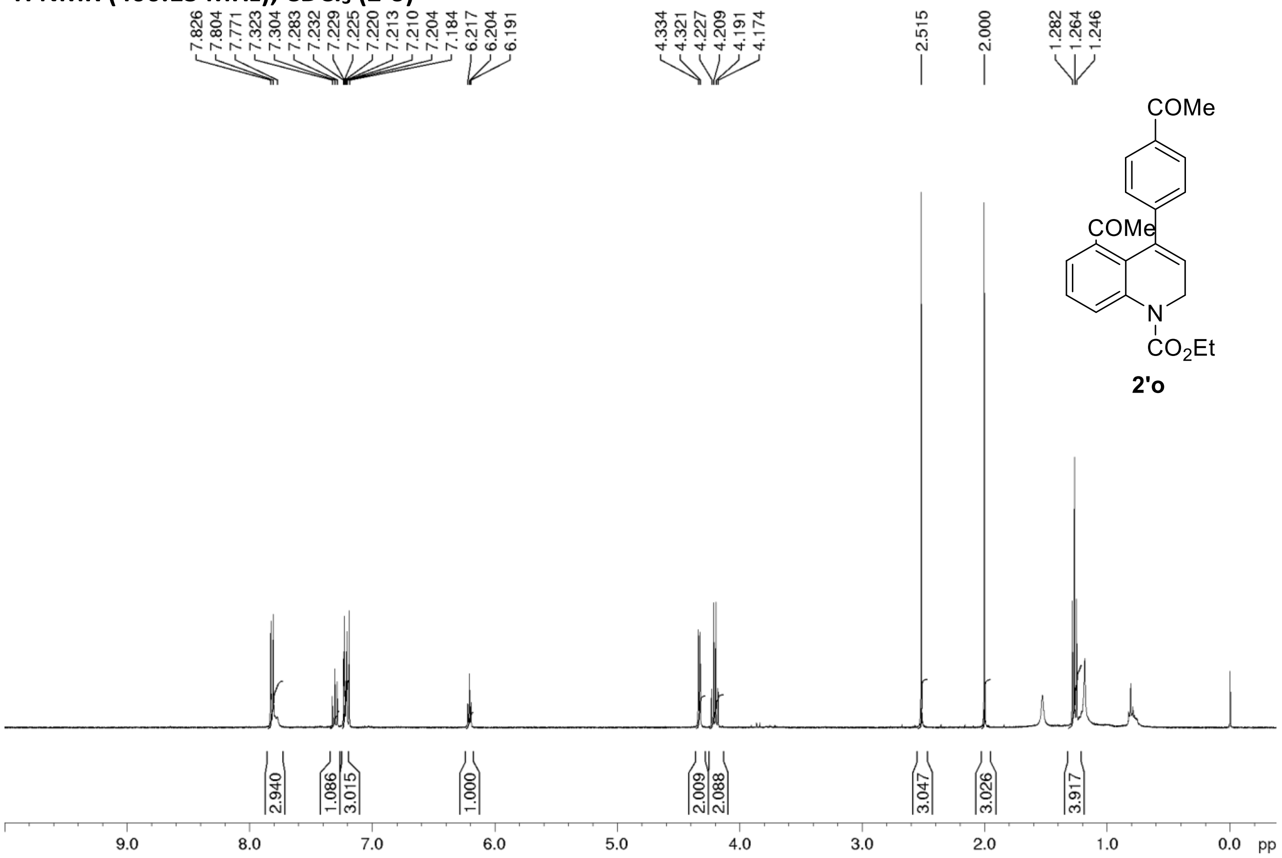

^{13}C NMR (100.6 MHz), $CDCl_3$ (2'o)

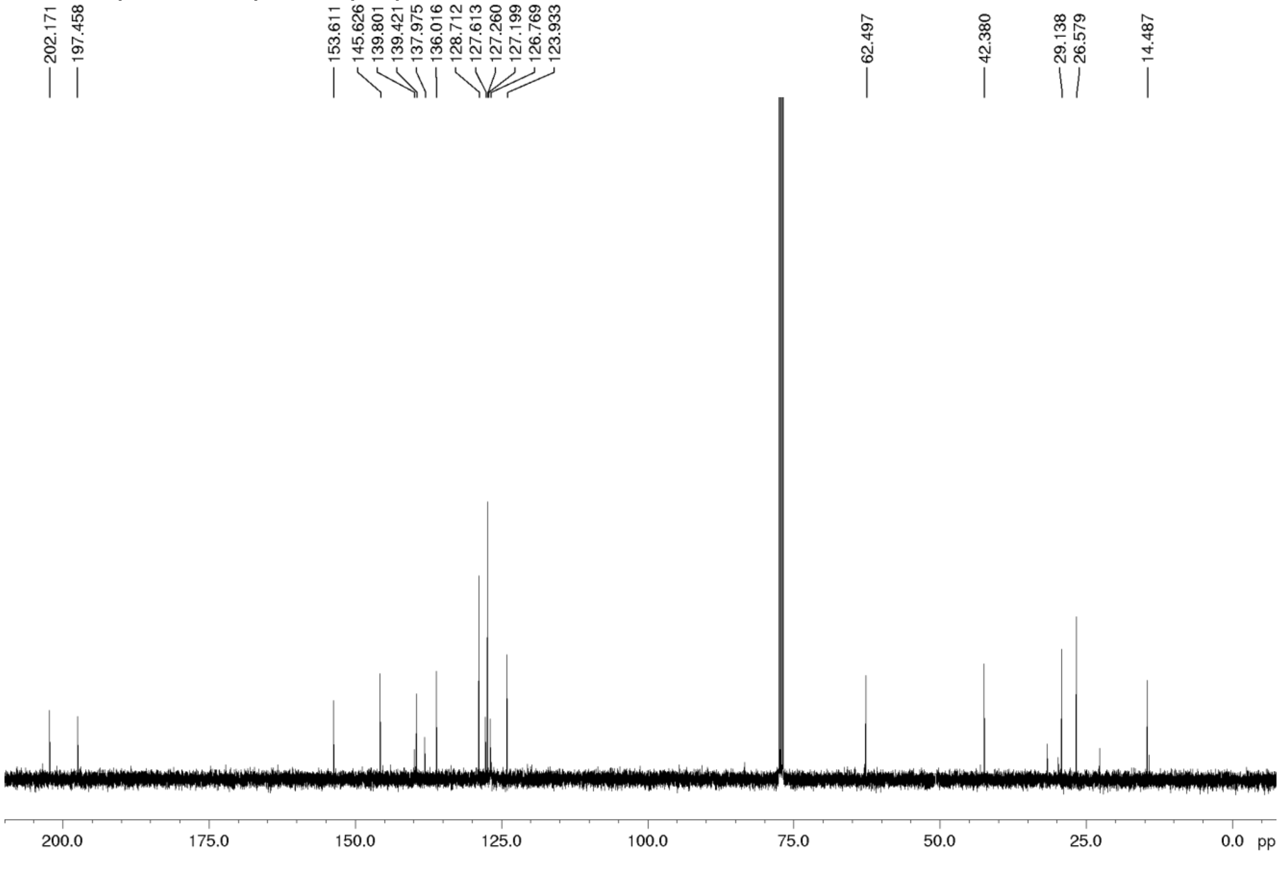

^{1}H NMR (400.13 MHz), $CDCl_3$ (3a)

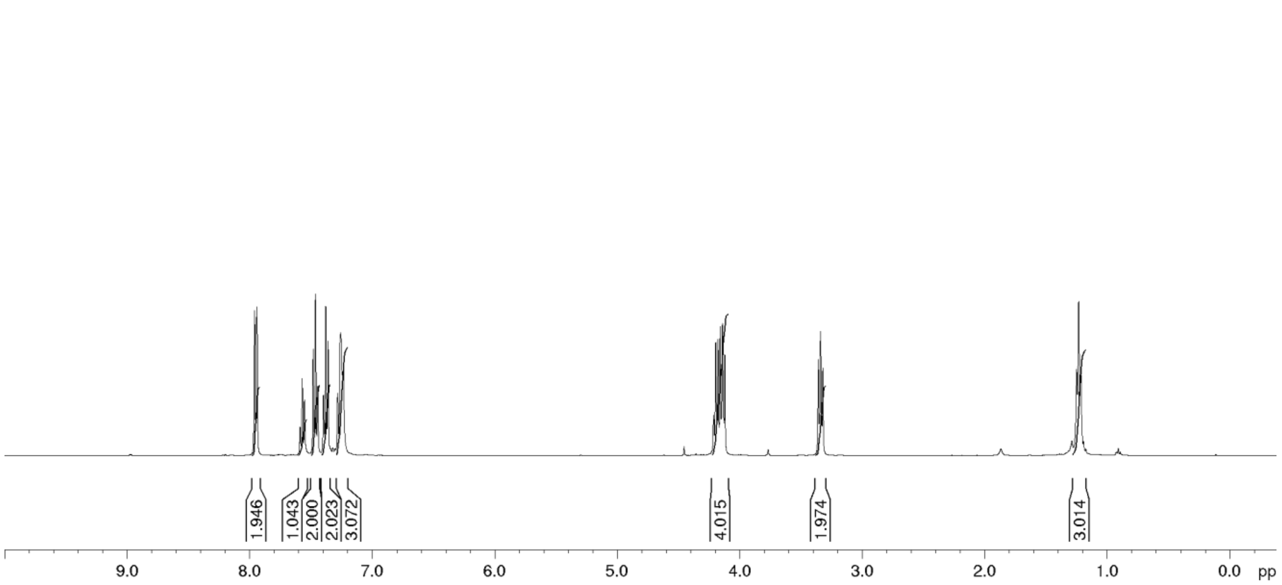

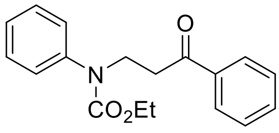

3a

^{13}C NMR (100.6 MHz), $CDCl_3$ (3a)

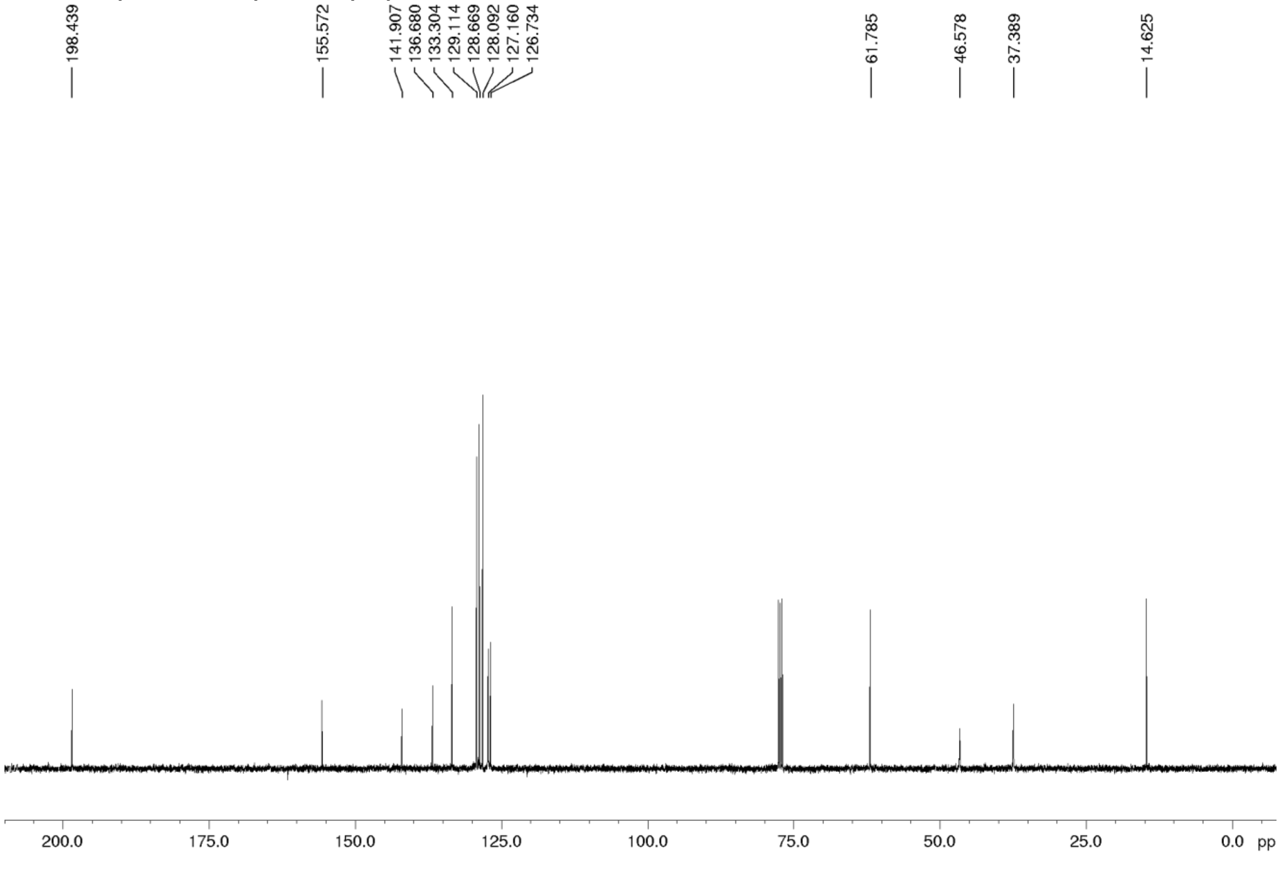

^{1}H NMR (400.13 MHz), CDCl$_3$ (5b)

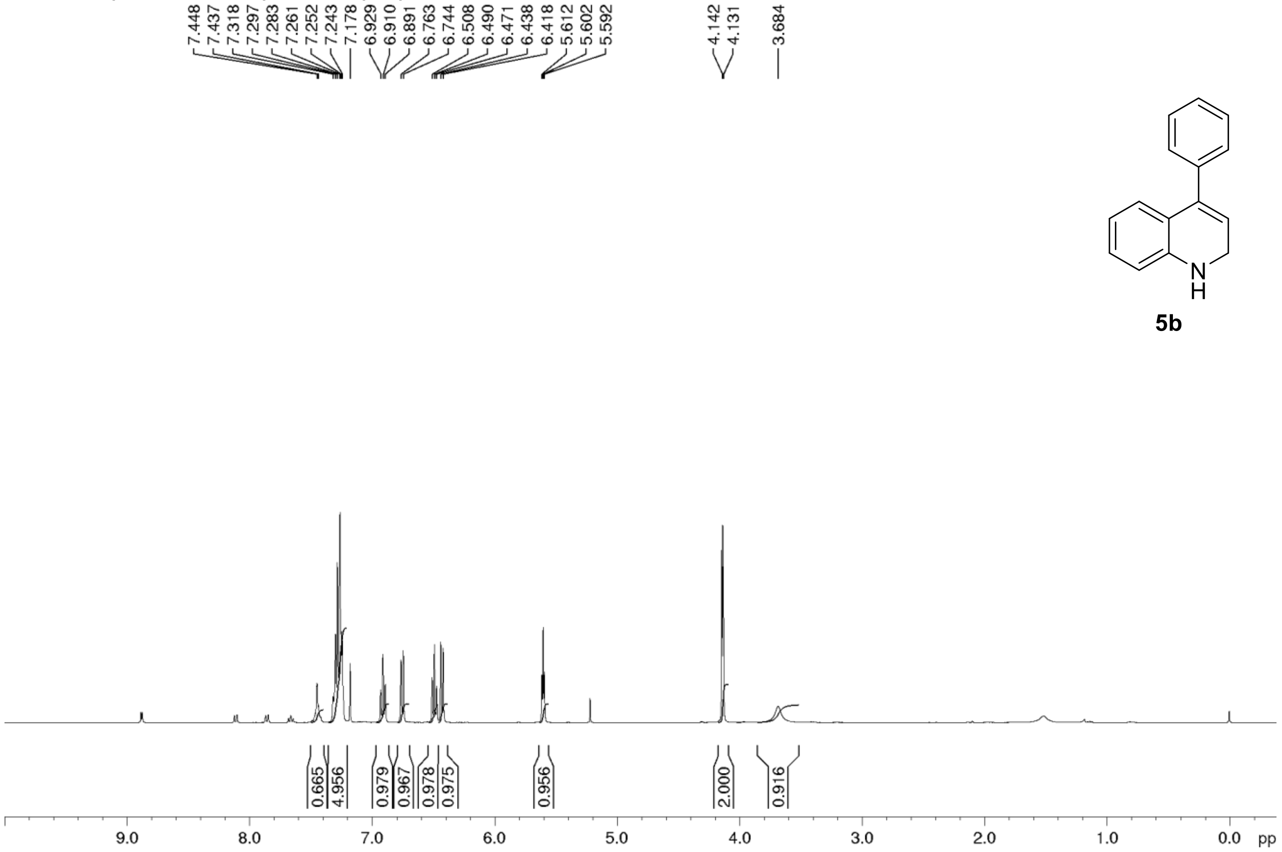

5b

^{13}C NMR (100.6 MHz), CDCl$_3$ (5b)

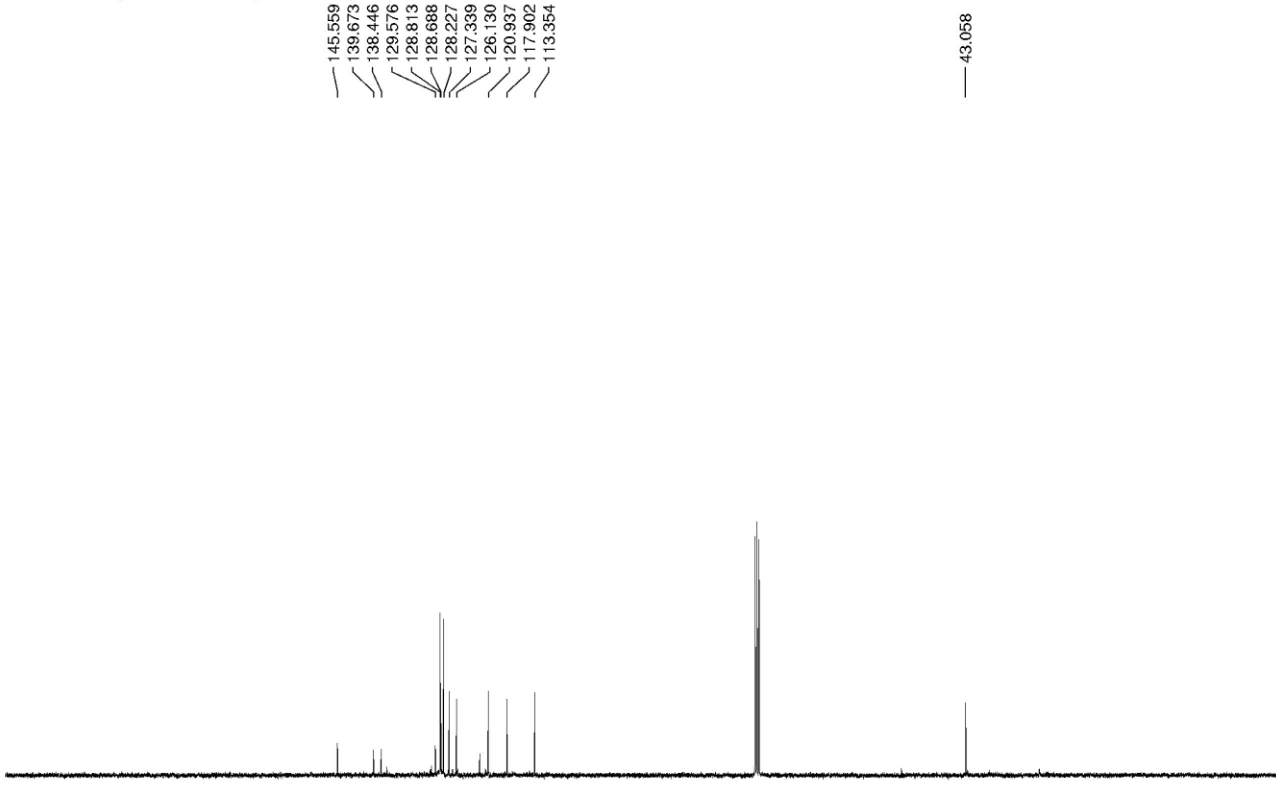

^{1}H NMR (400.13 MHz), $CDCl_3$ (6b)

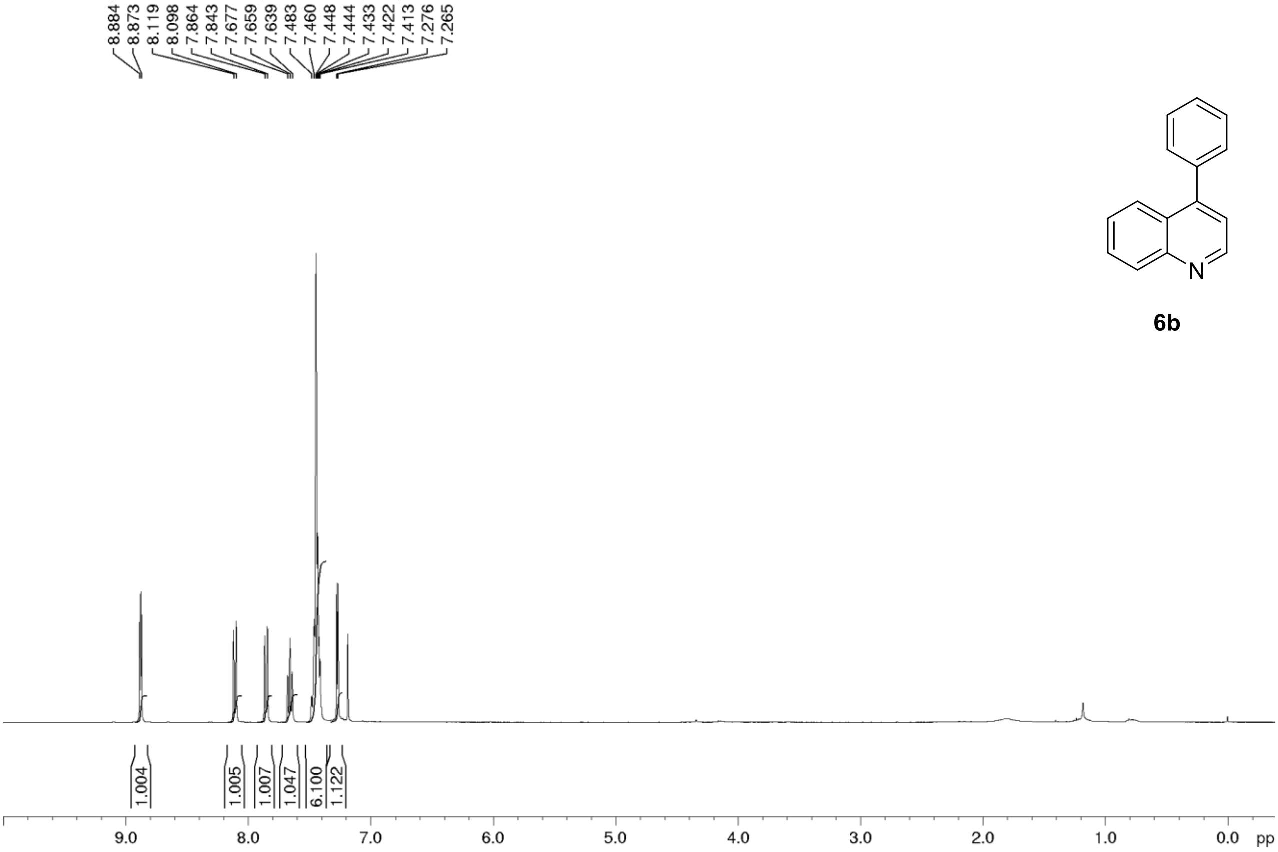

6b

^{13}C NMR (100.6 MHz), $CDCl_3$ (6b)

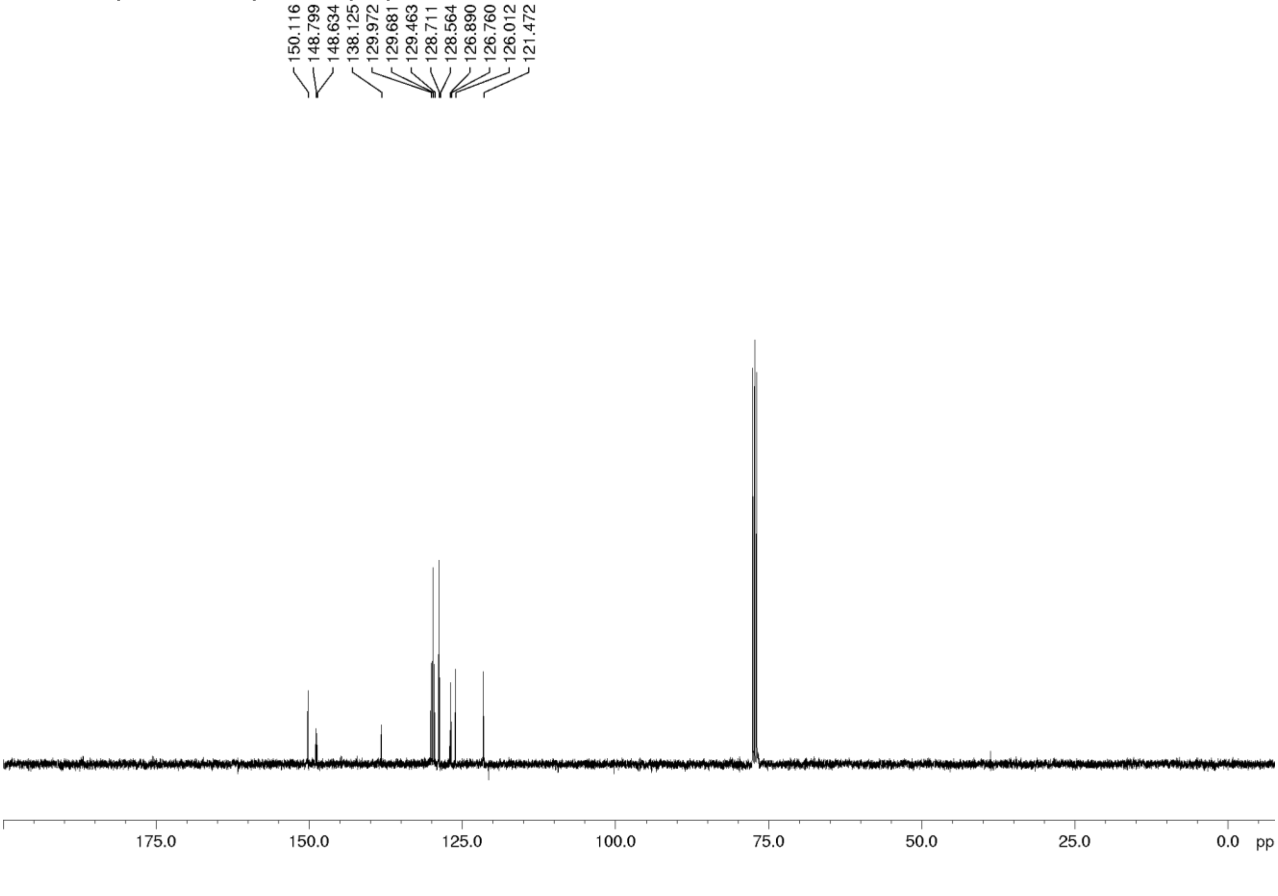

^{1}H NMR (400.13 MHz), $CDCl_3$ (7b)

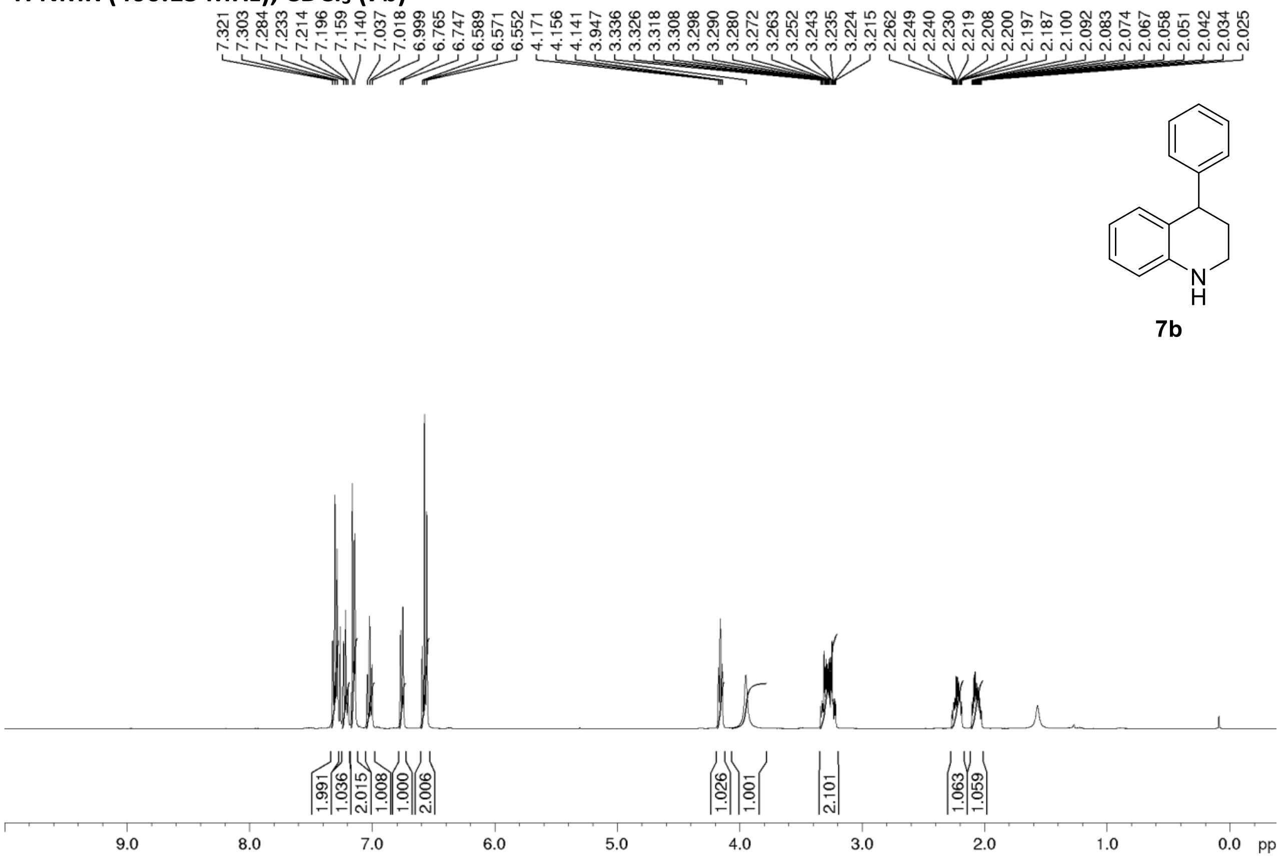

^{13}C NMR (100.6 MHz), $CDCl_3$ (7b)

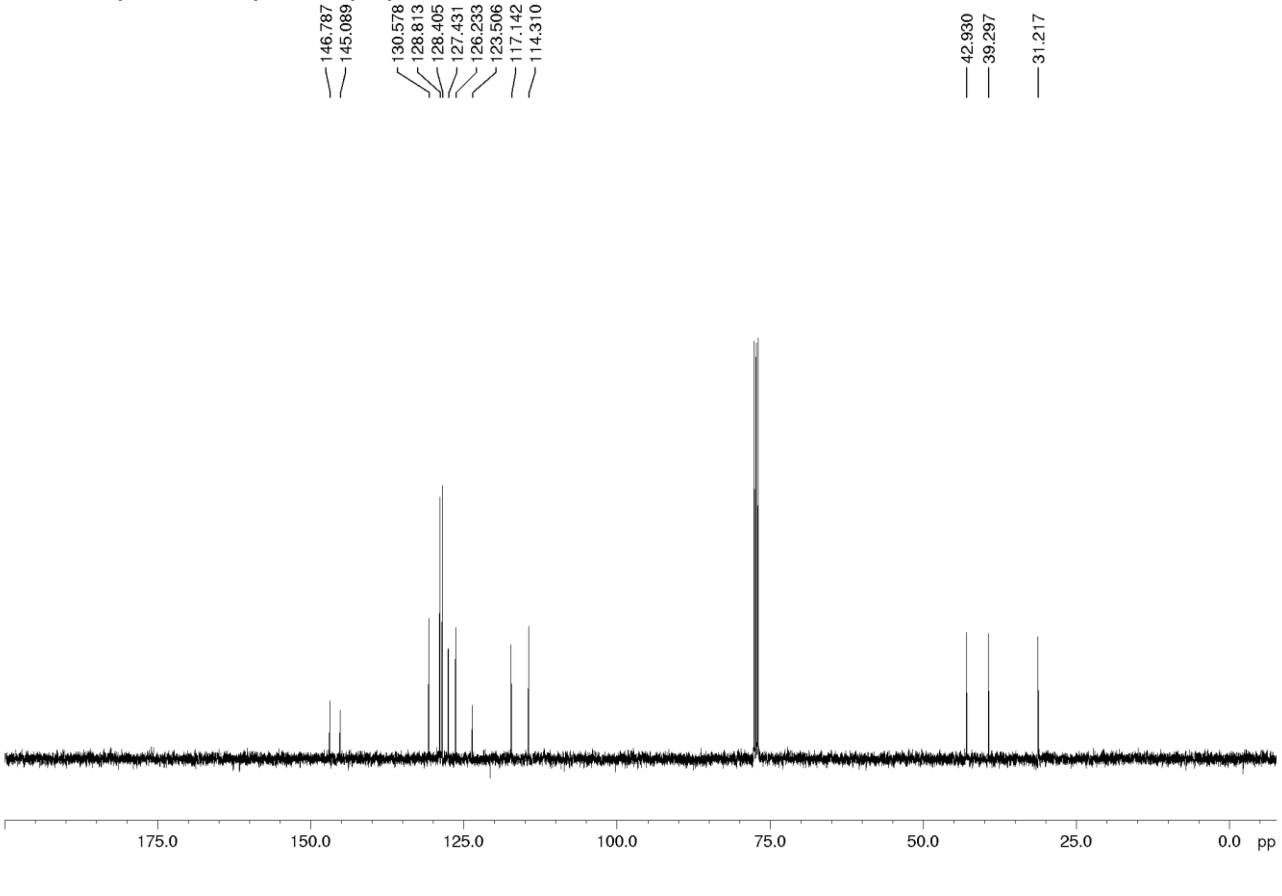

HRMS SPECTRA OF COMPOUNDS 2a – j, 2k – o, 2'k – o, 3a, 5b, 6b, 7b

HRMS (2a)

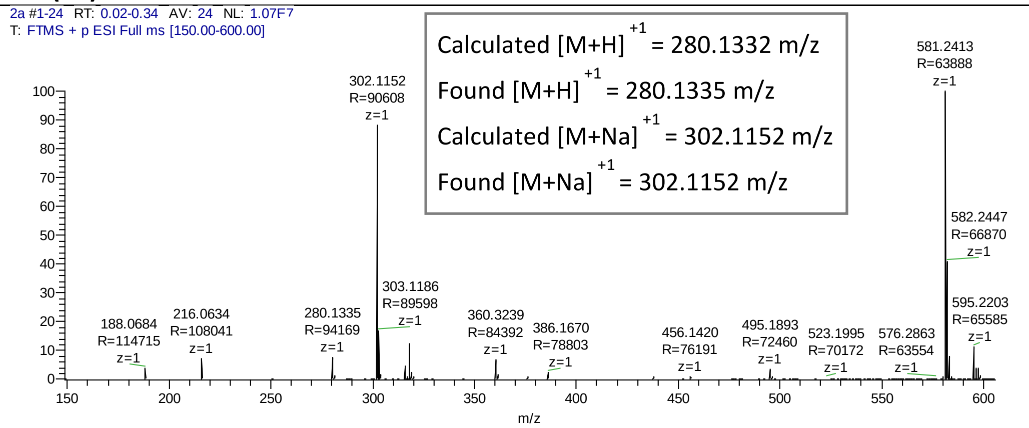

Calculated $[M+H]^{+1}$ = 280.1332 m/z

Found $[M+H]^{+1}$ = 280.1335 m/z

Calculated $[M+Na]^{+1}$ = 302.1152 m/z

Found $[M+Na]^{+1}$ = 302.1152 m/z

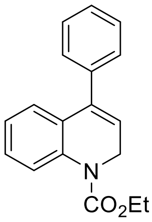

2a

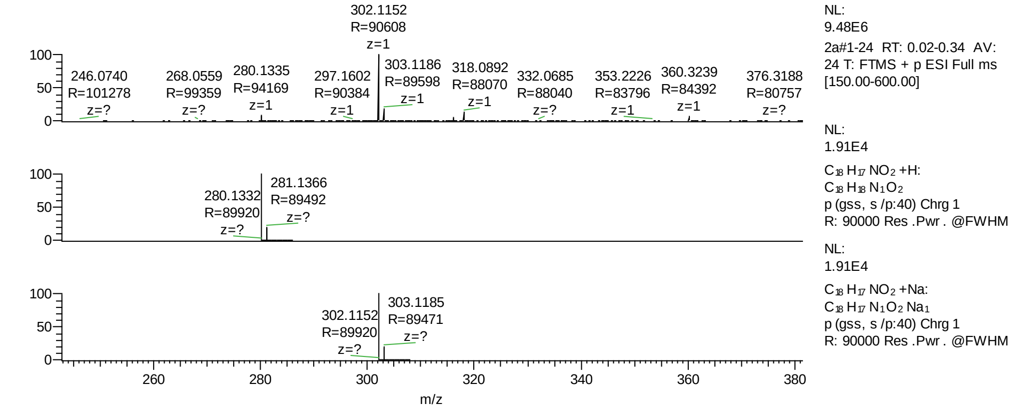

HRMS (2b)

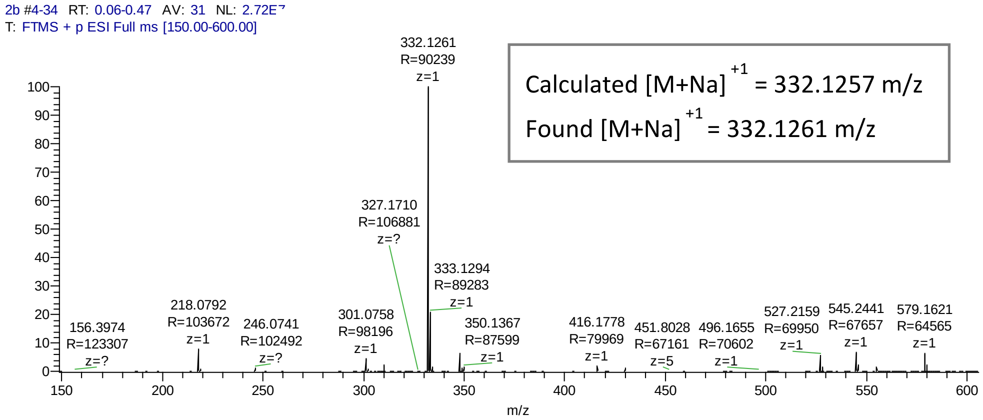

Calculated $[M+Na]^{+1}$ = 332.1257 m/z

Found $[M+Na]^{+1}$ = 332.1261 m/z

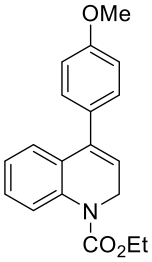

2b

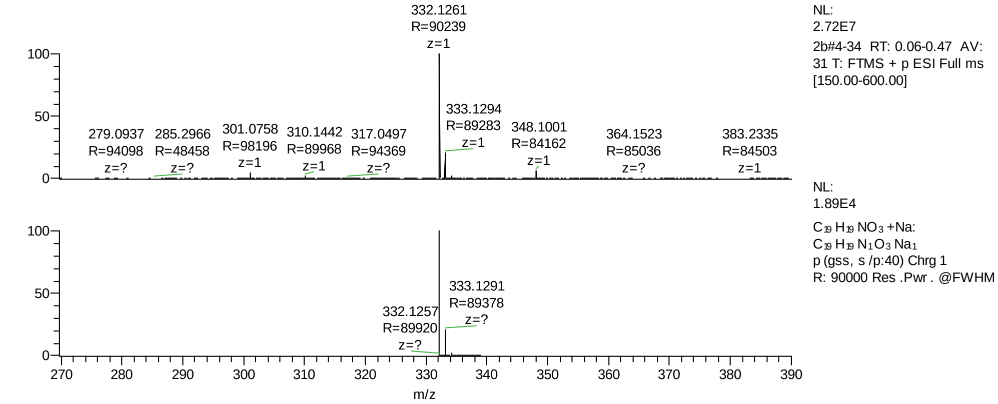

HRMS (2c)

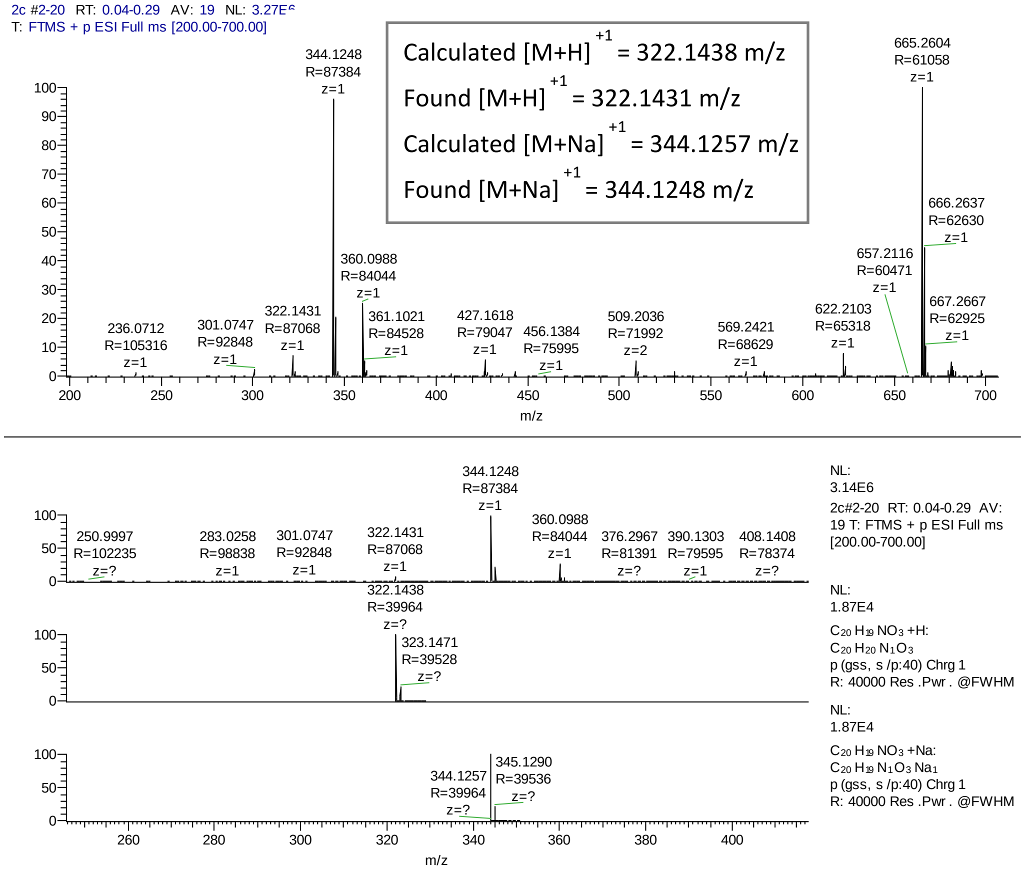

Calculated $[M+H]^{+1}$ = 322.1438 m/z

Found $[M+H]^{+1}$ = 322.1431 m/z

Calculated $[M+Na]^{+1}$ = 344.1257 m/z

Found $[M+Na]^{+1}$ = 344.1248 m/z

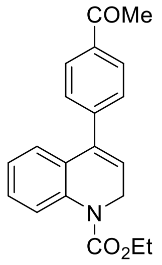

2c

HRMS (2d)

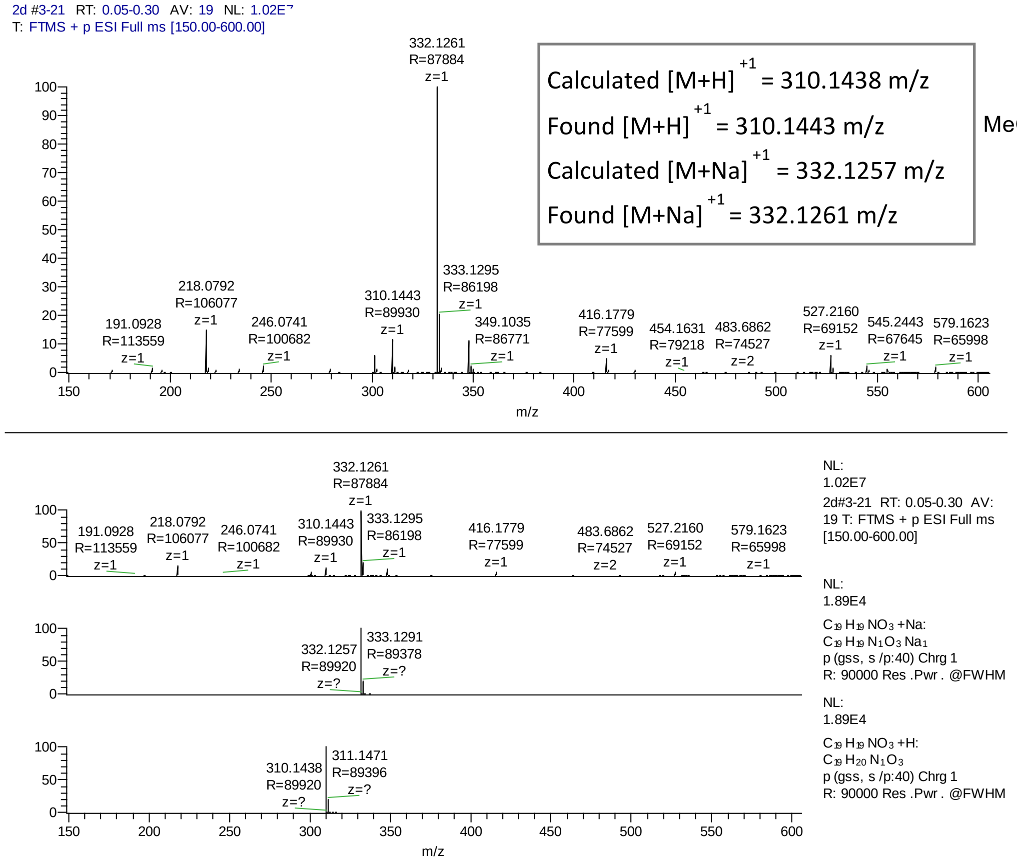

Calculated $[M+H]^{+1}$ = 310.1438 m/z

Found $[M+H]^{+1}$ = 310.1443 m/z

Calculated $[M+Na]^{+1}$ = 332.1257 m/z

Found $[M+Na]^{+1}$ = 332.1261 m/z

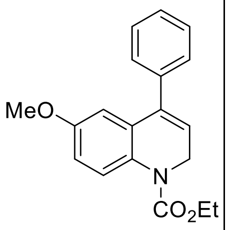

2d

HRMS (2e)

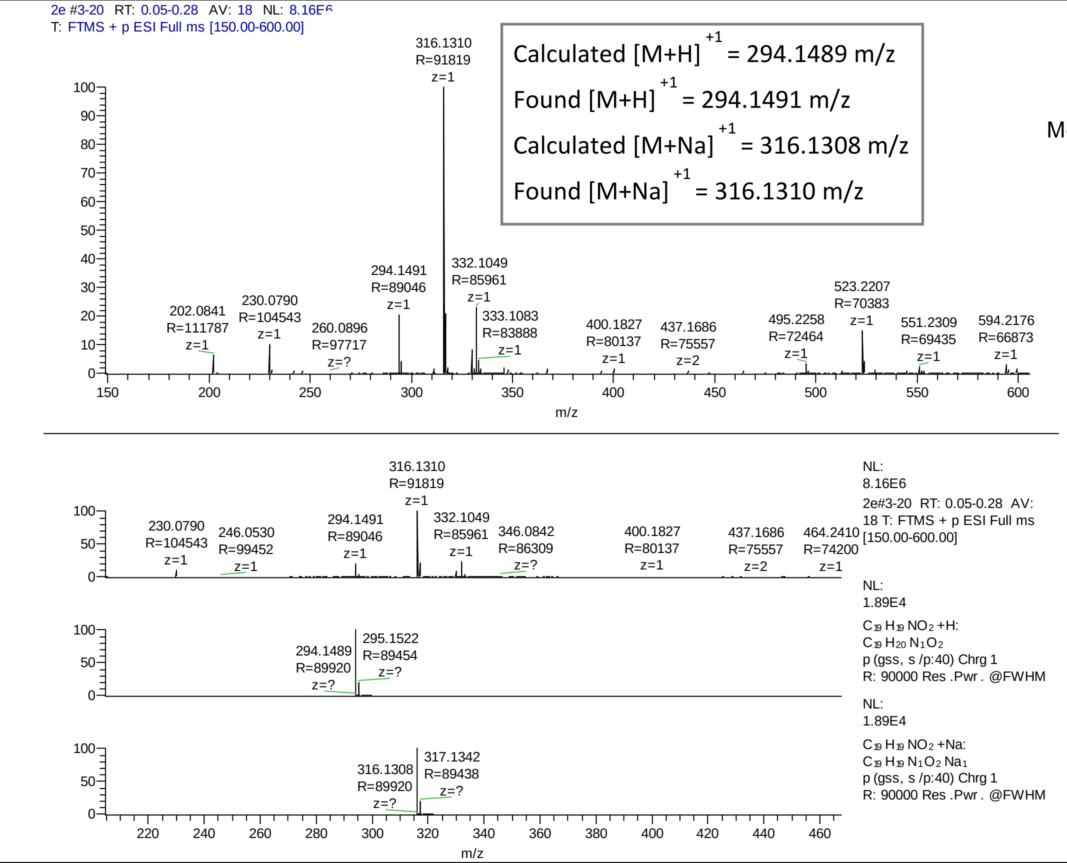

Calculated $[M+H]^{+1}$ = 294.1489 m/z

Found $[M+H]^{+1}$ = 294.1491 m/z

Calculated $[M+Na]^{+1}$ = 316.1308 m/z

Found $[M+Na]^{+1}$ = 316.1310 m/z

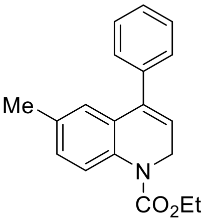

2e

HRMS (2f)

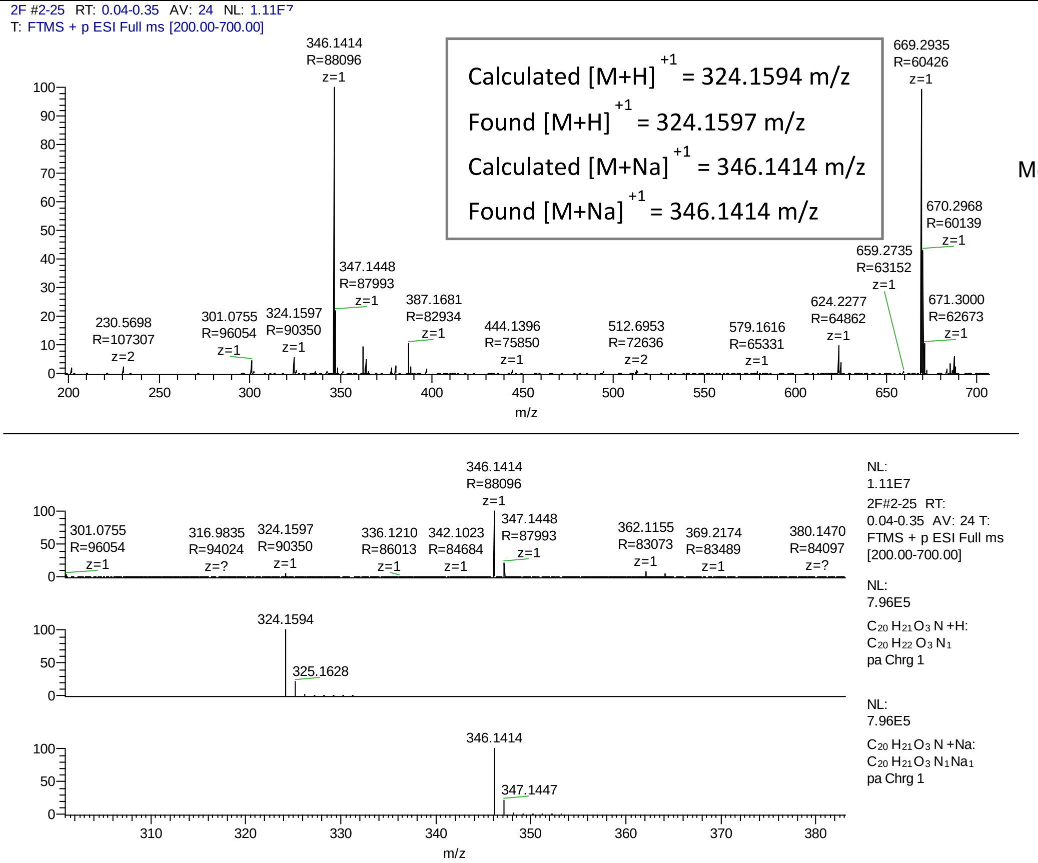

Calculated $[M+H]^{+1}$ = 324.1594 m/z

Found $[M+H]^{+1}$ = 324.1597 m/z

Calculated $[M+Na]^{+1}$ = 346.1414 m/z

Found $[M+Na]^{+1}$ = 346.1414 m/z

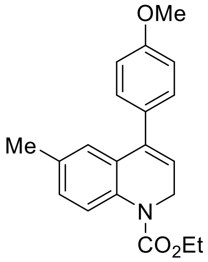

2f

HRMS (2g)

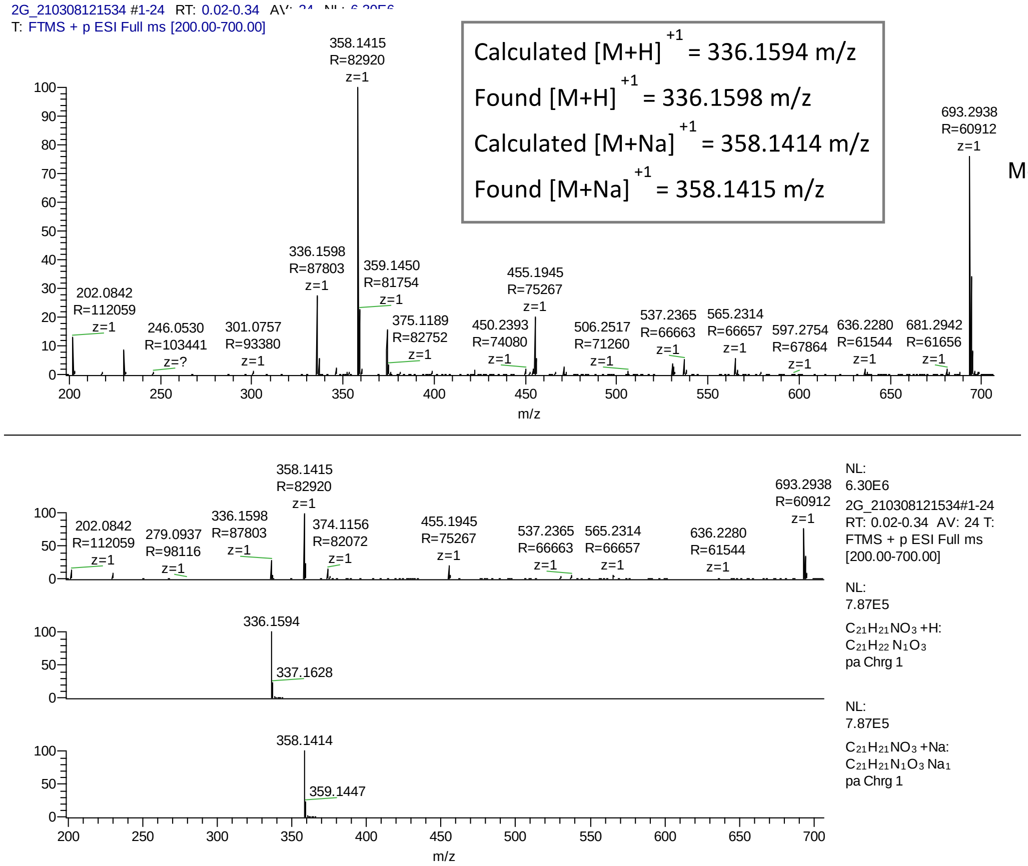

Calculated $[M+H]^{+1}$ = 336.1594 m/z

Found $[M+H]^{+1}$ = 336.1598 m/z

Calculated $[M+Na]^{+1}$ = 358.1414 m/z

Found $[M+Na]^{+1}$ = 358.1415 m/z

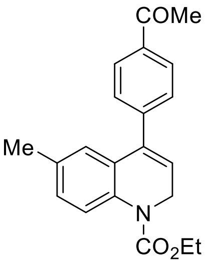

2g

HRMS (2h)

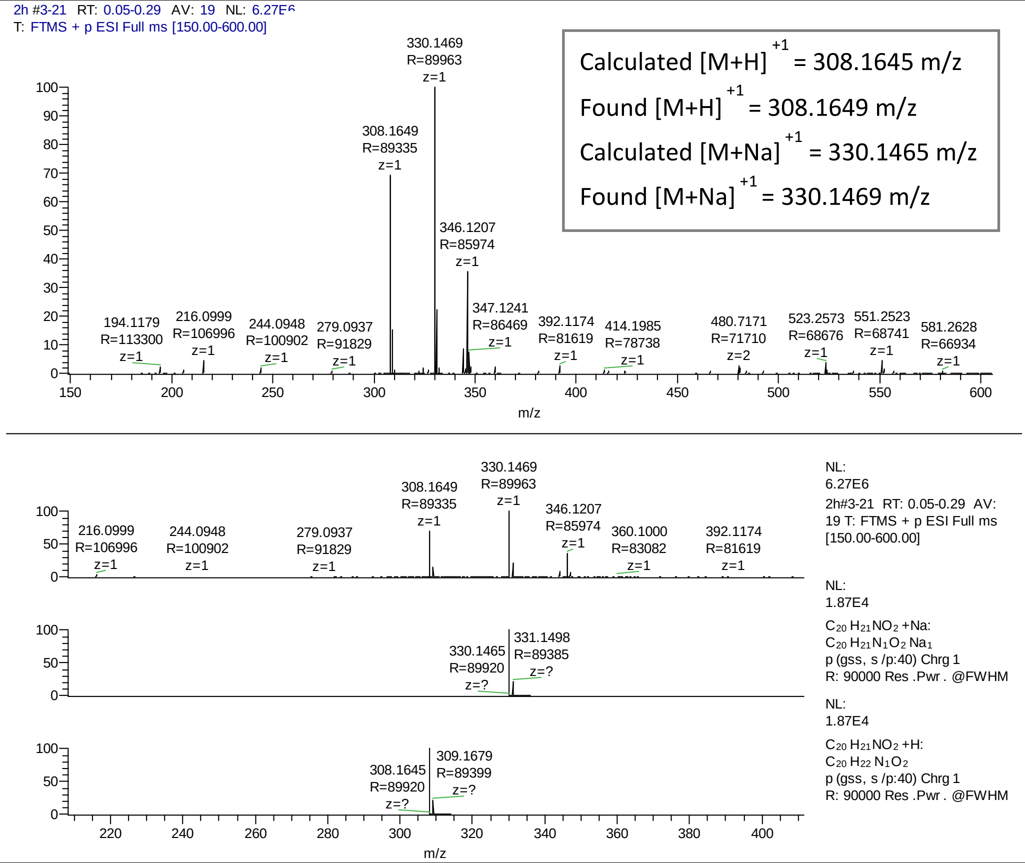

Calculated $[M+H]^{+1}$ = 308.1645 m/z

Found $[M+H]^{+1}$ = 308.1649 m/z

Calculated $[M+Na]^{+1}$ = 330.1465 m/z

Found $[M+Na]^{+1}$ = 330.1469 m/z

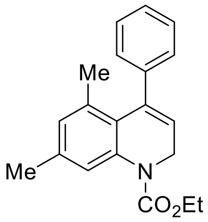

2h

HRMS (2i)

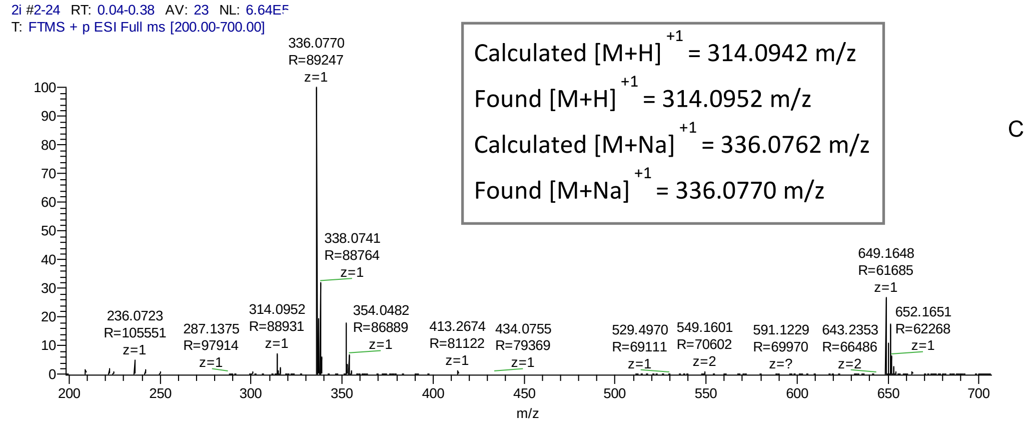

Calculated $[M+H]^{+1}$ = 314.0942 m/z

Found $[M+H]^{+1}$ = 314.0952 m/z

Calculated $[M+Na]^{+1}$ = 336.0762 m/z

Found $[M+Na]^{+1}$ = 336.0770 m/z

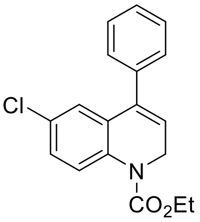

2i

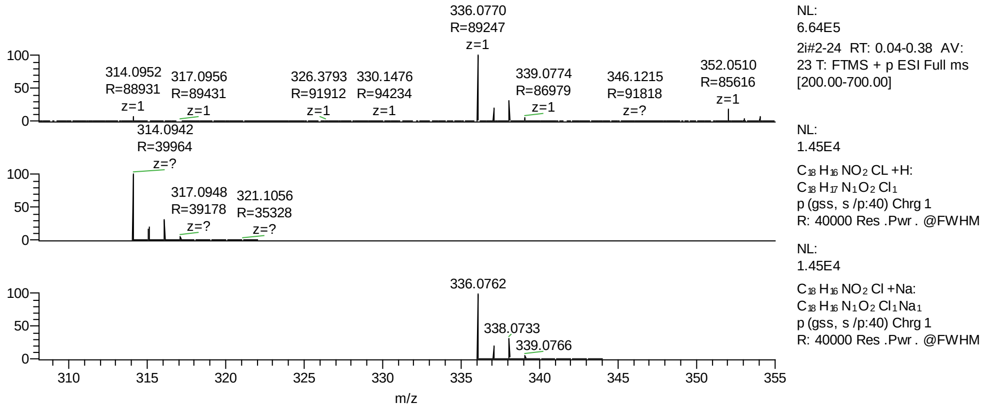

HRMS (2j)

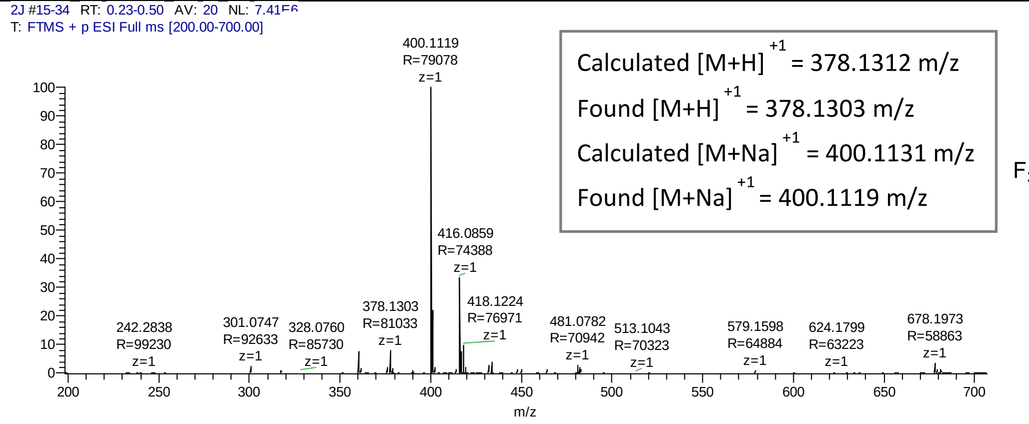

Calculated $[M+H]^{+1}$ = 378.1312 m/z

Found $[M+H]^{+1}$ = 378.1303 m/z

Calculated $[M+Na]^{+1}$ = 400.1131 m/z

Found $[M+Na]^{+1}$ = 400.1119 m/z

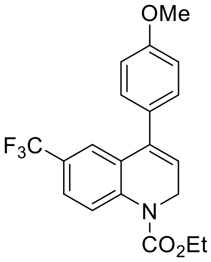

2j

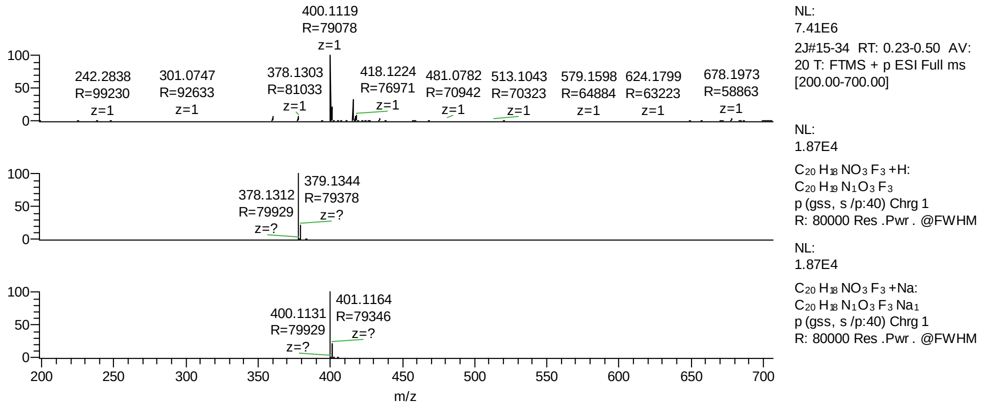

HRMS (2k)

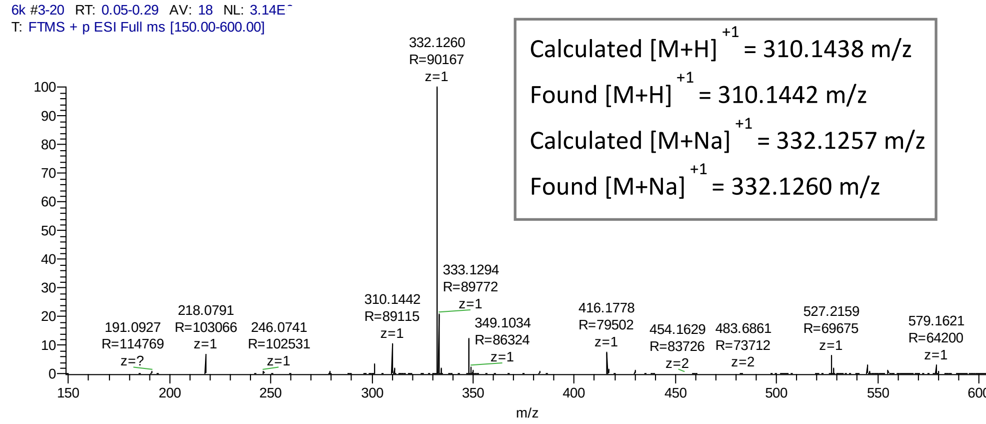

Calculated $[M+H]^{+1}$ = 310.1438 m/z

Found $[M+H]^{+1}$ = 310.1442 m/z

Calculated $[M+Na]^{+1}$ = 332.1257 m/z

Found $[M+Na]^{+1}$ = 332.1260 m/z

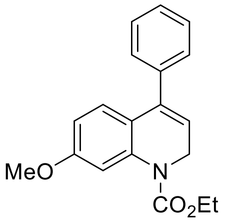

2k

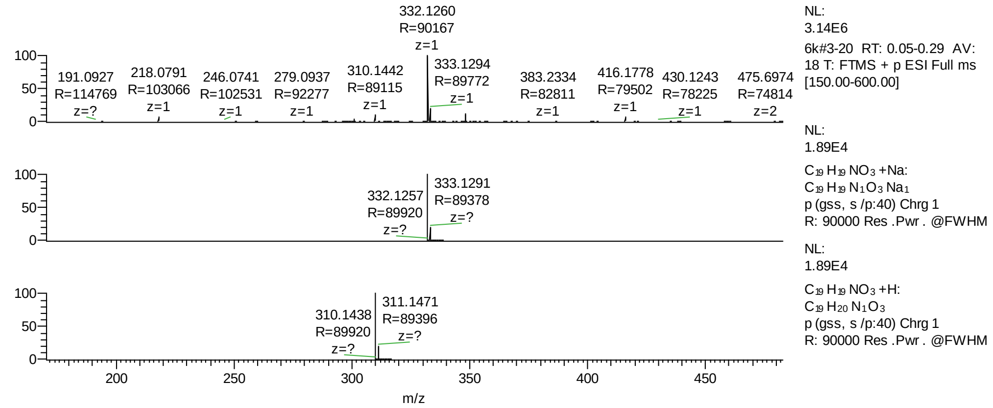

HRMS (2'k)

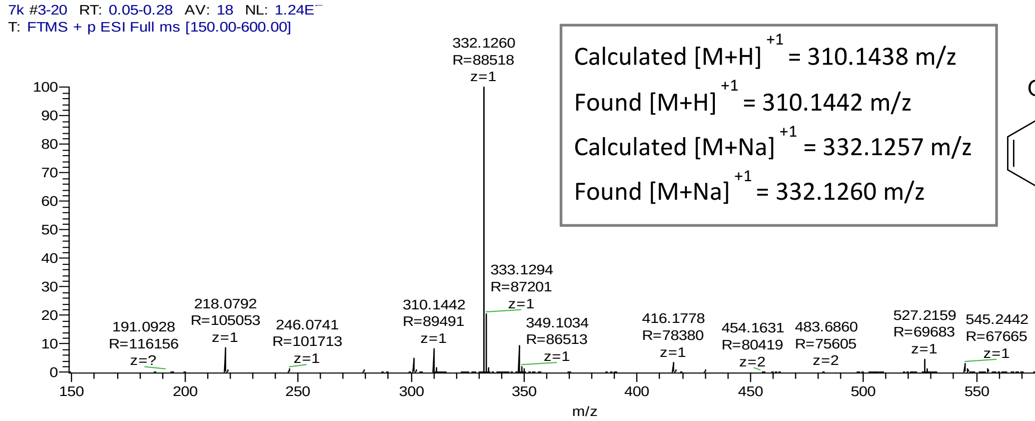

Calculated $[M+H]^{+1}$ = 310.1438 m/z

Found $[M+H]^{+1}$ = 310.1442 m/z

Calculated $[M+Na]^{+1}$ = 332.1257 m/z

Found $[M+Na]^{+1}$ = 332.1260 m/z

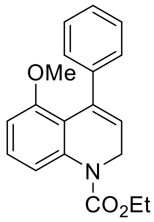

2'k

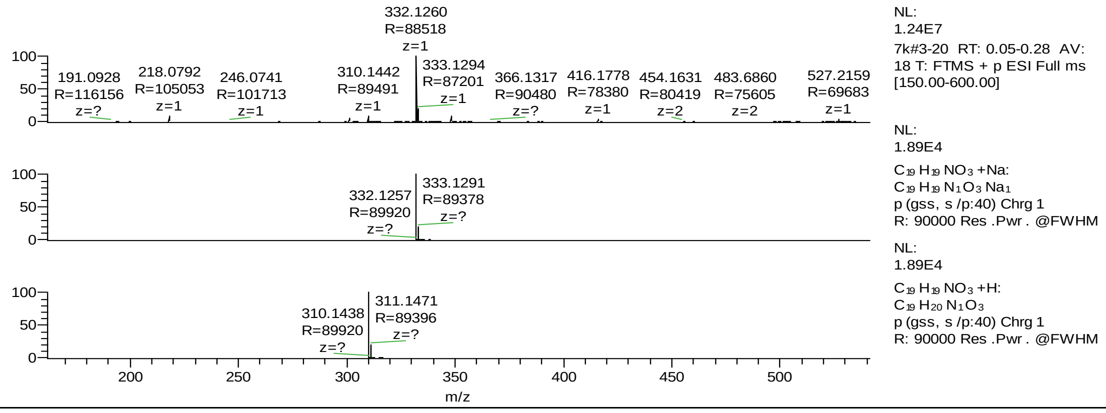

HRMS (2l + 2'l)

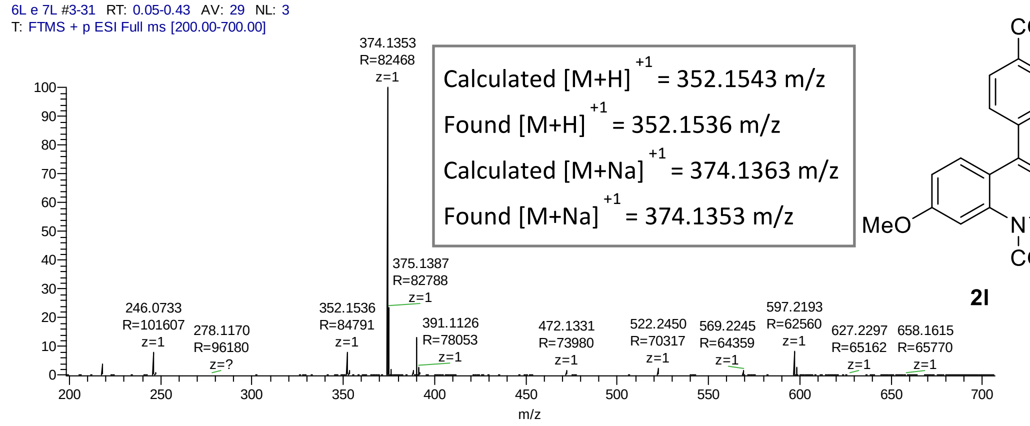

Calculated $[M+H]^{+1}$ = 352.1543 m/z

Found $[M+H]^{+1}$ = 352.1536 m/z

Calculated $[M+Na]^{+1}$ = 374.1363 m/z

Found $[M+Na]^{+1}$ = 374.1353 m/z

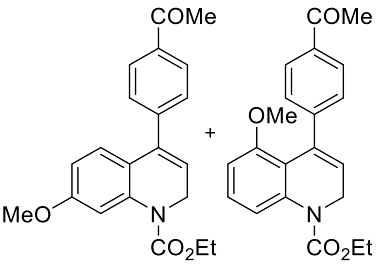

2l **2'l**

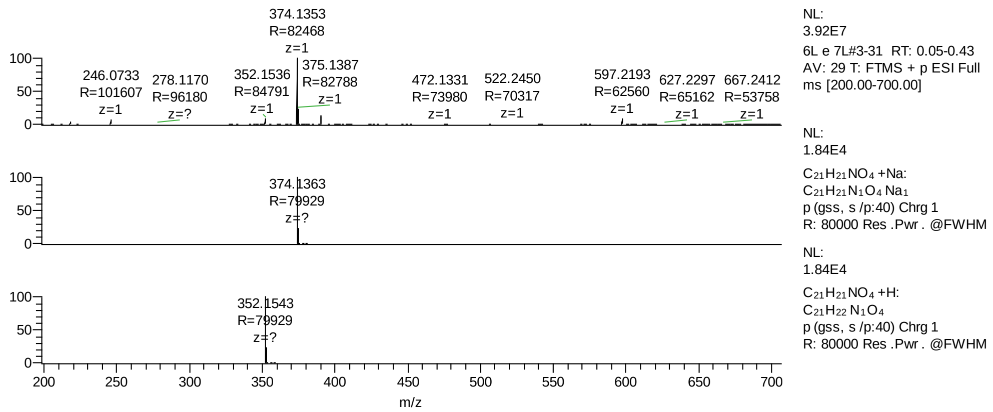

HRMS (2m)

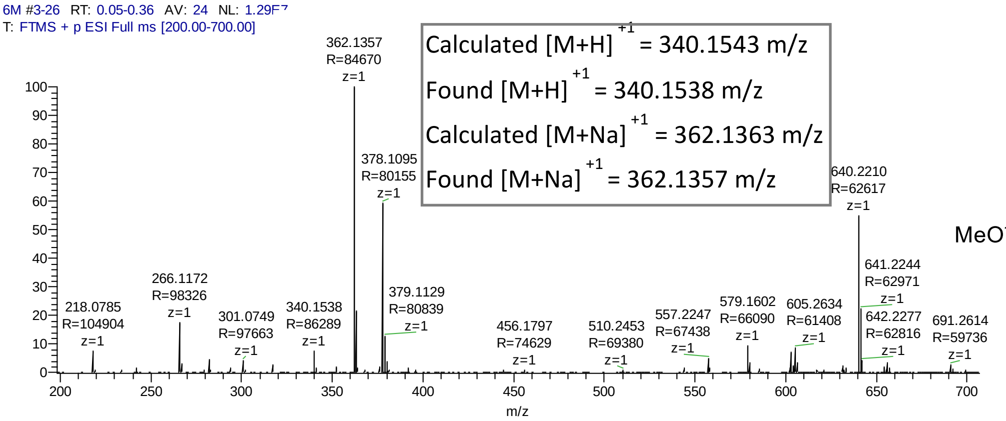

Calculated $[M+H]^{+1}$ = 340.1543 m/z

Found $[M+H]^{+1}$ = 340.1538 m/z

Calculated $[M+Na]^{+1}$ = 362.1363 m/z

Found $[M+Na]^{+1}$ = 362.1357 m/z

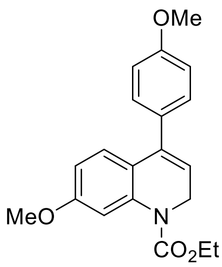

2m

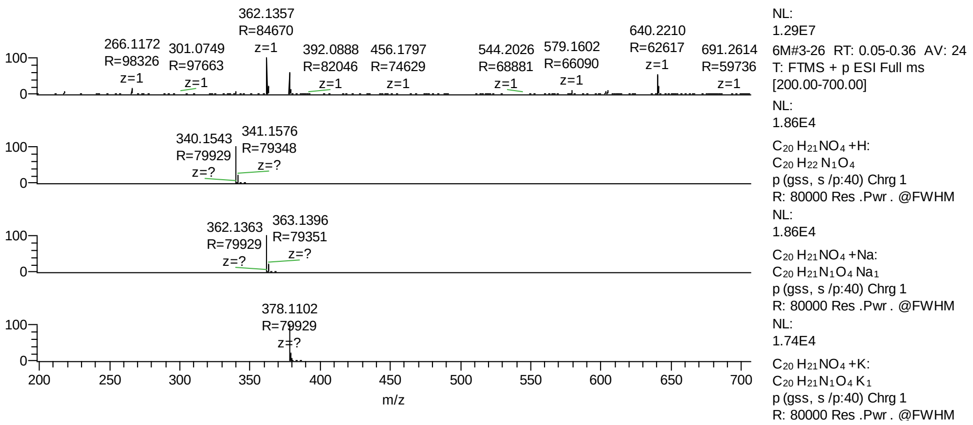

HRMS (2’m)

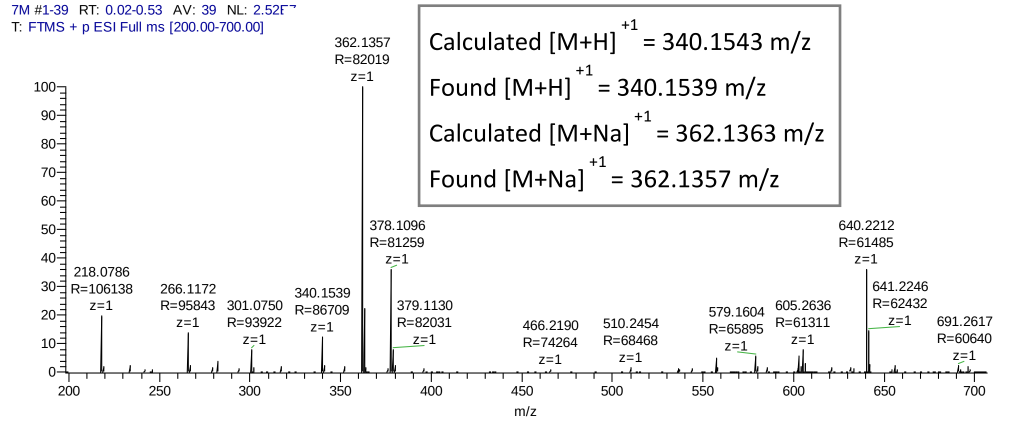

Calculated $[M+H]^{+1}$ = 340.1543 m/z

Found $[M+H]^{+1}$ = 340.1539 m/z

Calculated $[M+Na]^{+1}$ = 362.1363 m/z

Found $[M+Na]^{+1}$ = 362.1357 m/z

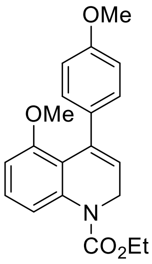

2'm

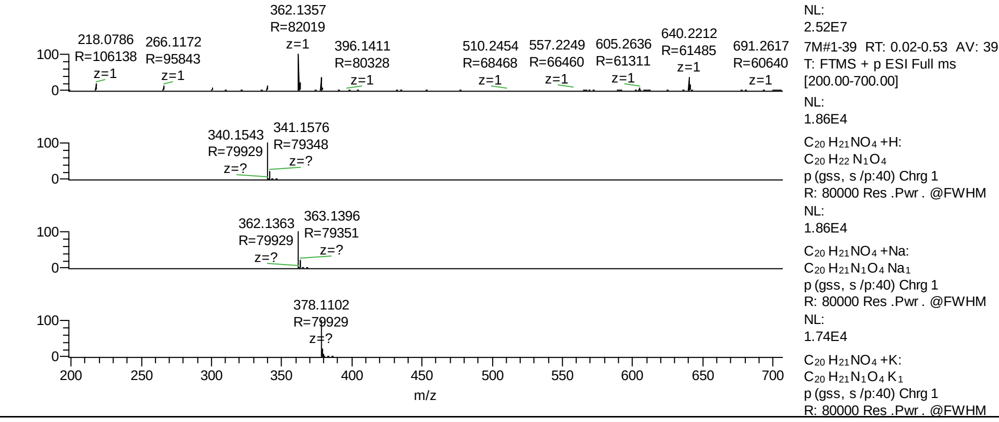

HRMS (2n)

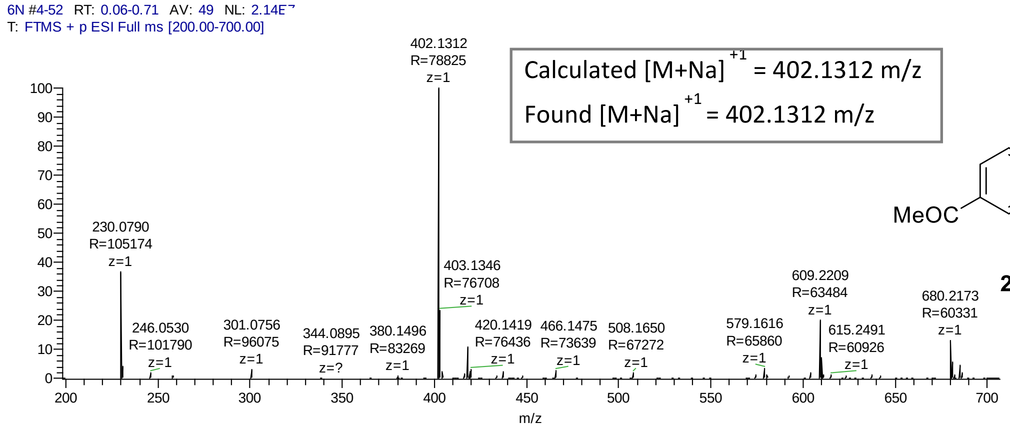

Calculated $[M+Na]^{+1}$ = 402.1312 m/z

Found $[M+Na]^{+1}$ = 402.1312 m/z

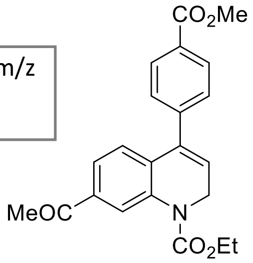

2n

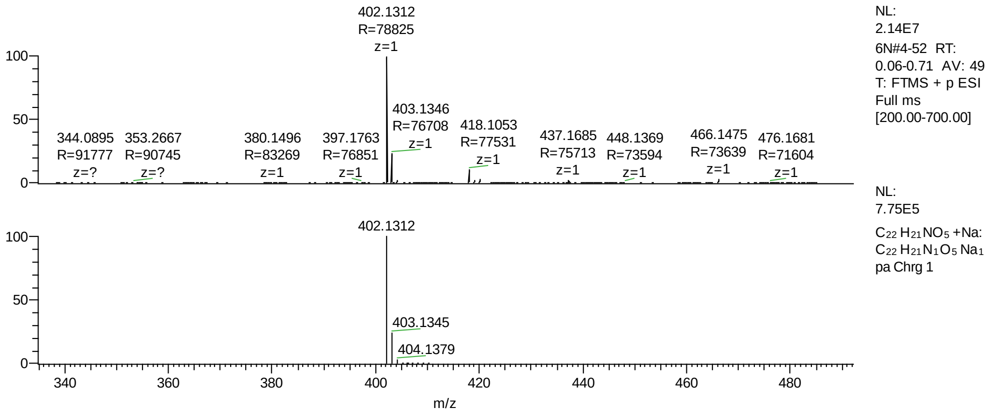

HRMS (2'n)

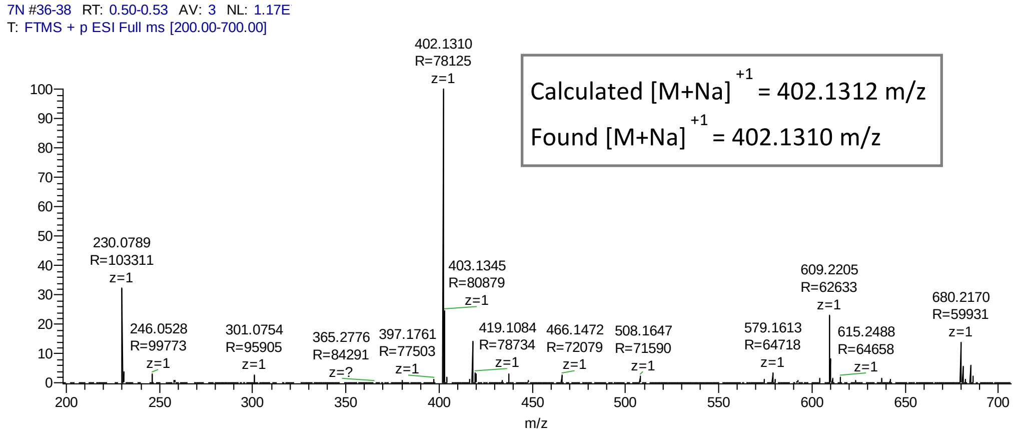

Calculated $[M+Na]^{+1}$ = 402.1312 m/z

Found $[M+Na]^{+1}$ = 402.1310 m/z

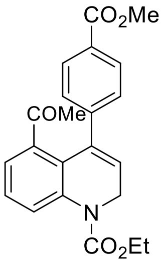

2'n

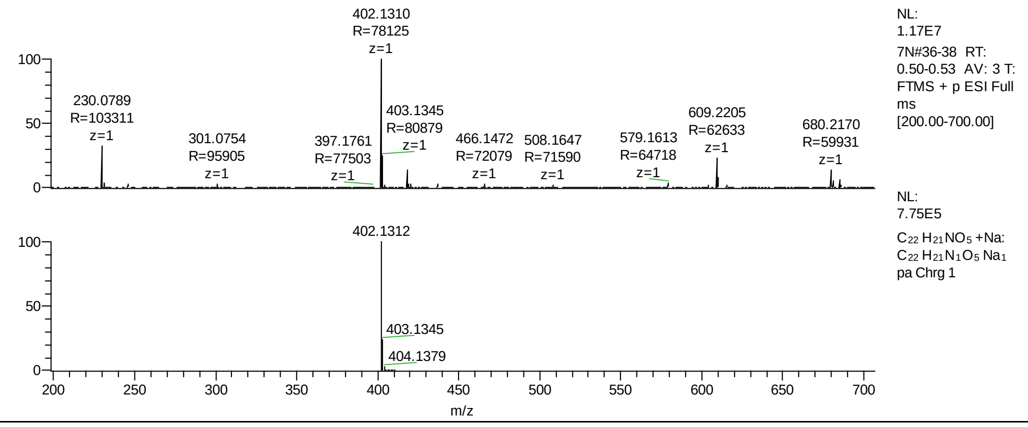

HRMS (2o)

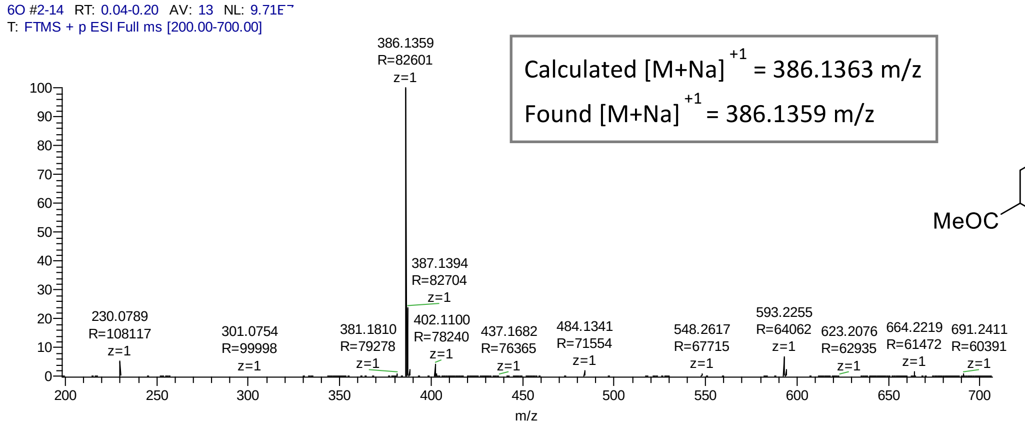

Calculated $[M+Na]^{+1}$ = 386.1363 m/z

Found $[M+Na]^{+1}$ = 386.1359 m/z

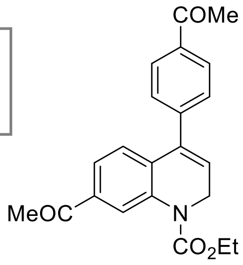

2o

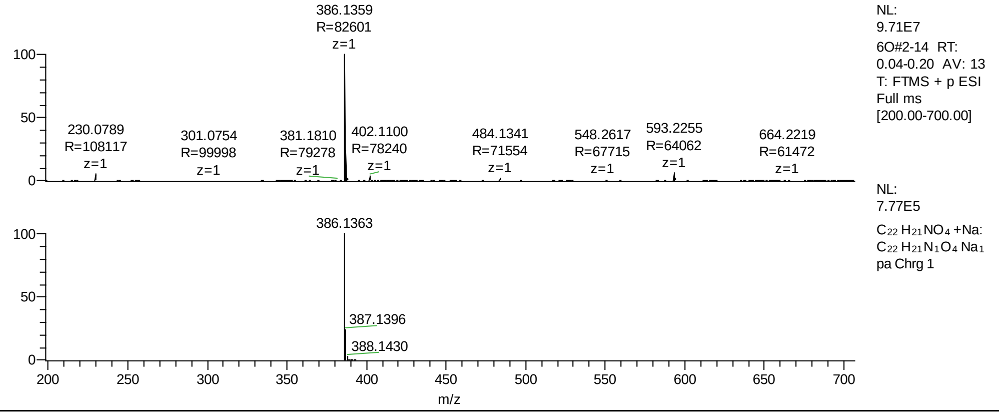

HRMS (2’o)

Calculated $[M+Na]^{+1}$ = 386.1363 m/z

Found $[M+Na]^{+1}$ = 386.1361 m/z

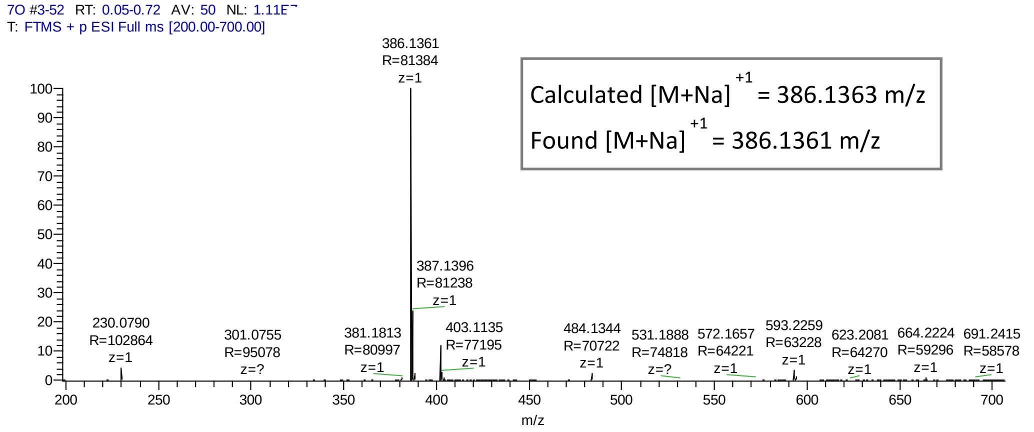

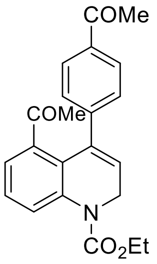

2'o

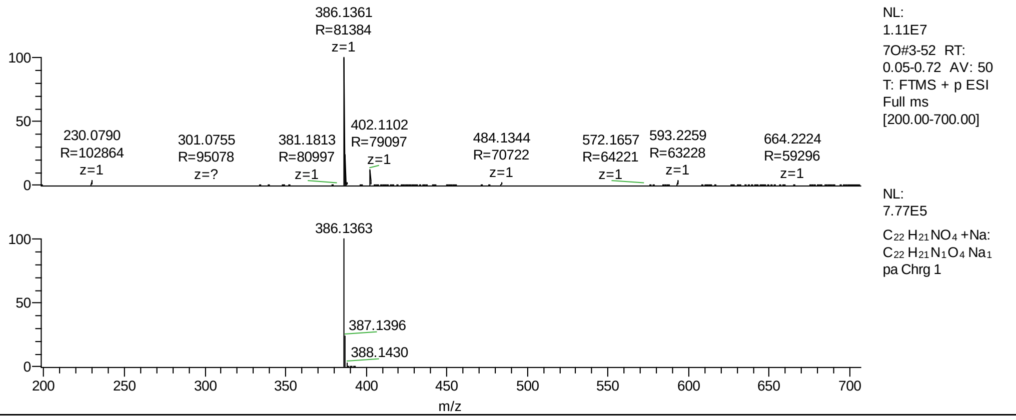

HRMS (3a)

Calculated $[M+Na]^{+1}$ = 320.1257 m/z

Found $[M+Na]^{+1}$ = 320.1253 m/z

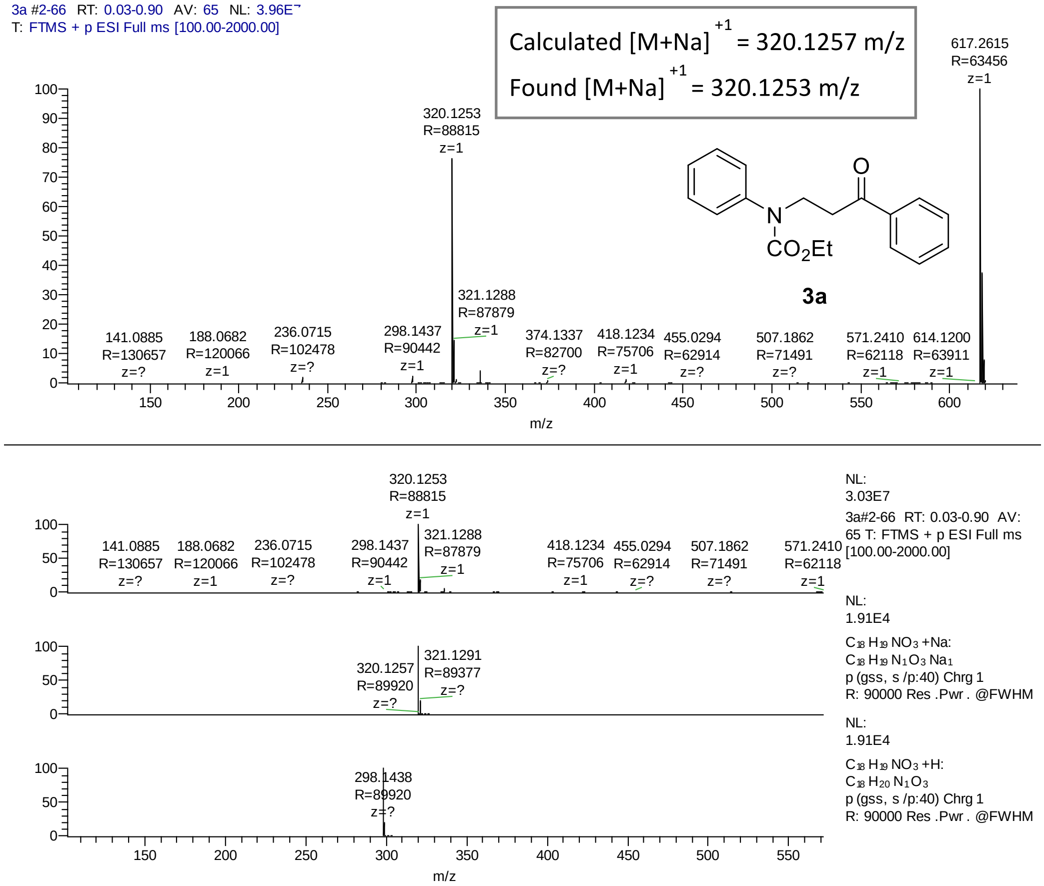

3a

HRMS (4a)

Calculated $[M+Na]^{+1}$ = 326.0763 m/z

Found $[M+Na]^{+1}$ = 326.0764 m/z

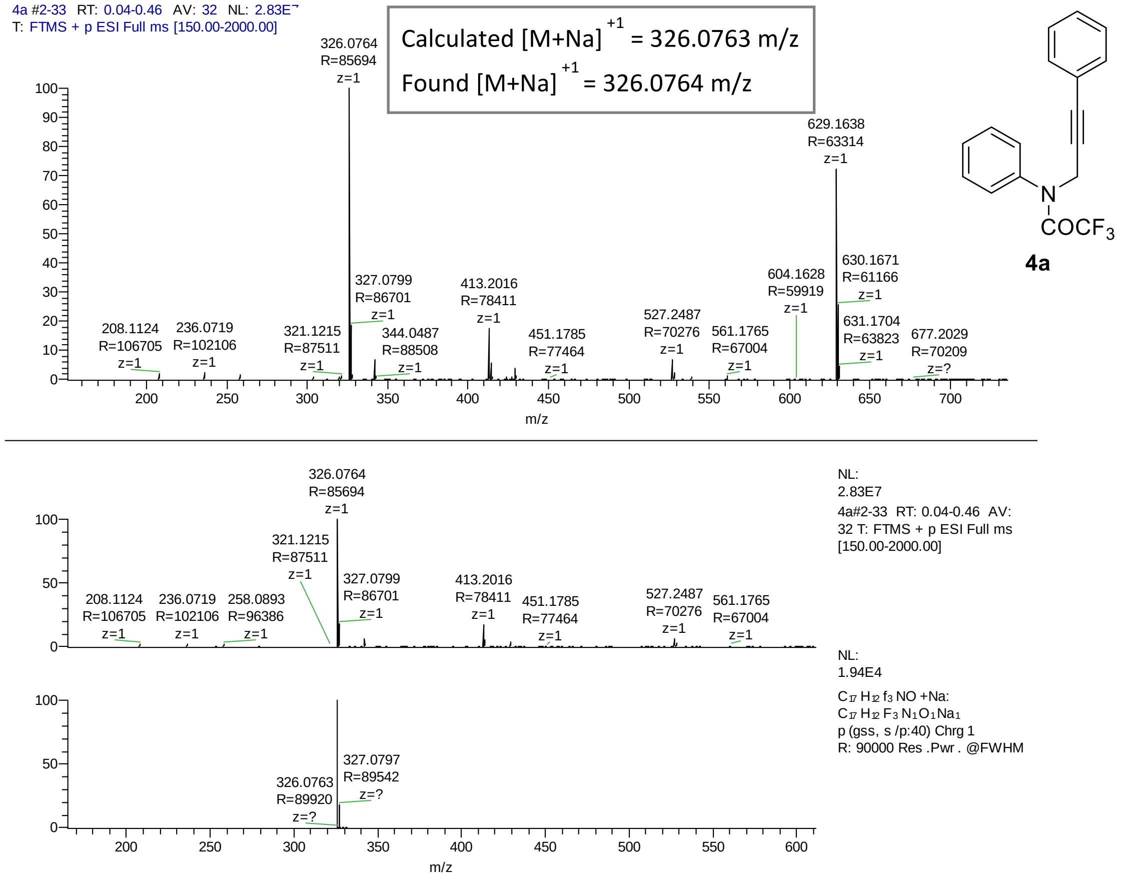

4a

HRMS (4b)

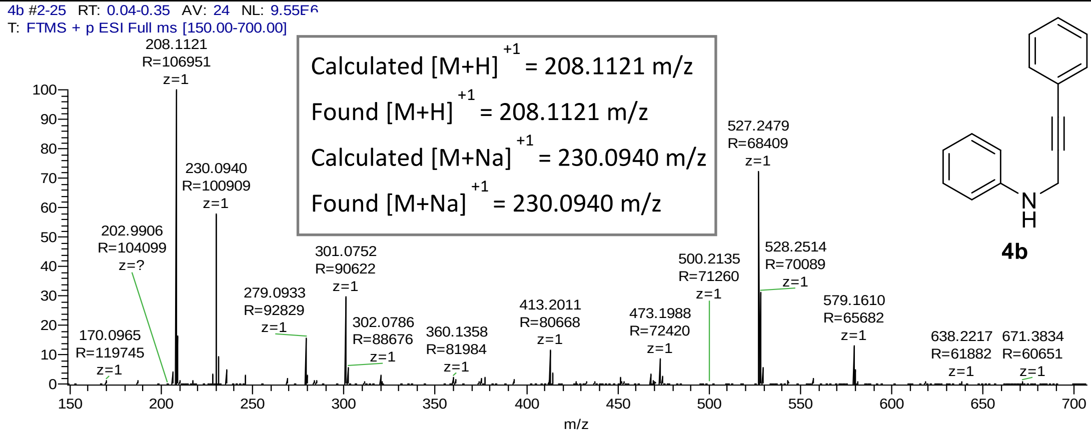

Calculated $[M+H]^{+1}$ = 208.1121 m/z

Found $[M+H]^{+1}$ = 208.1121 m/z

Calculated $[M+Na]^{+1}$ = 230.0940 m/z

Found $[M+Na]^{+1}$ = 230.0940 m/z

4b

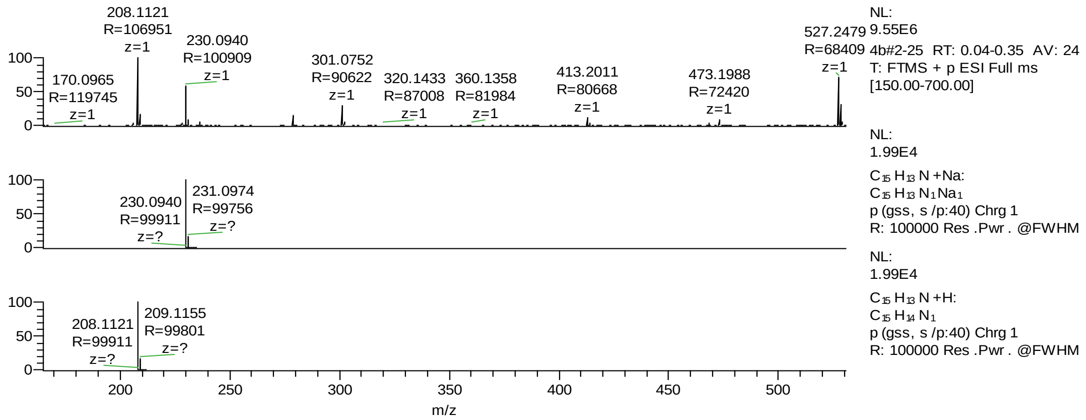

HRMS (5b)

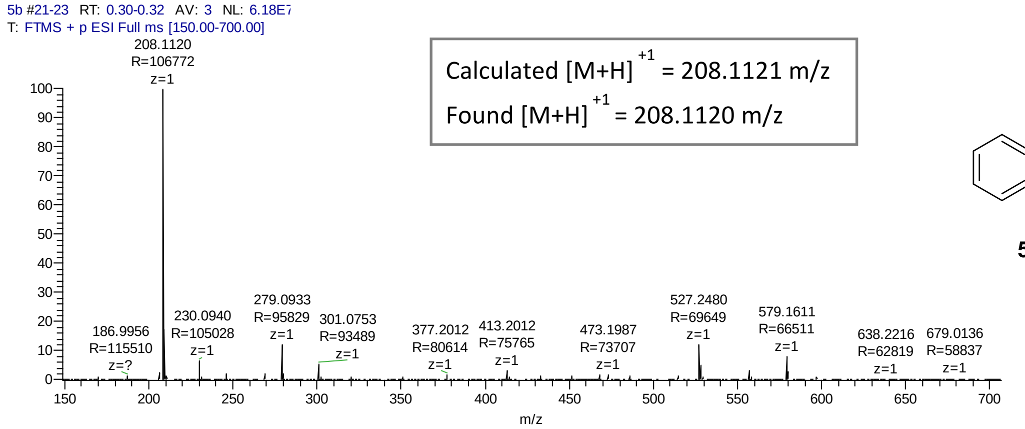

Calculated $[M+H]^{+1}$ = 208.1121 m/z

Found $[M+H]^{+1}$ = 208.1120 m/z

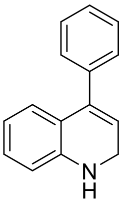

5b

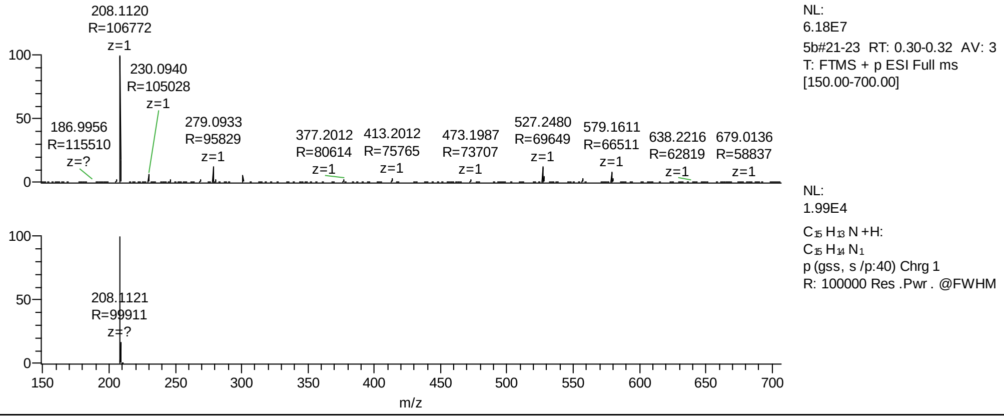

HRMS (6b)

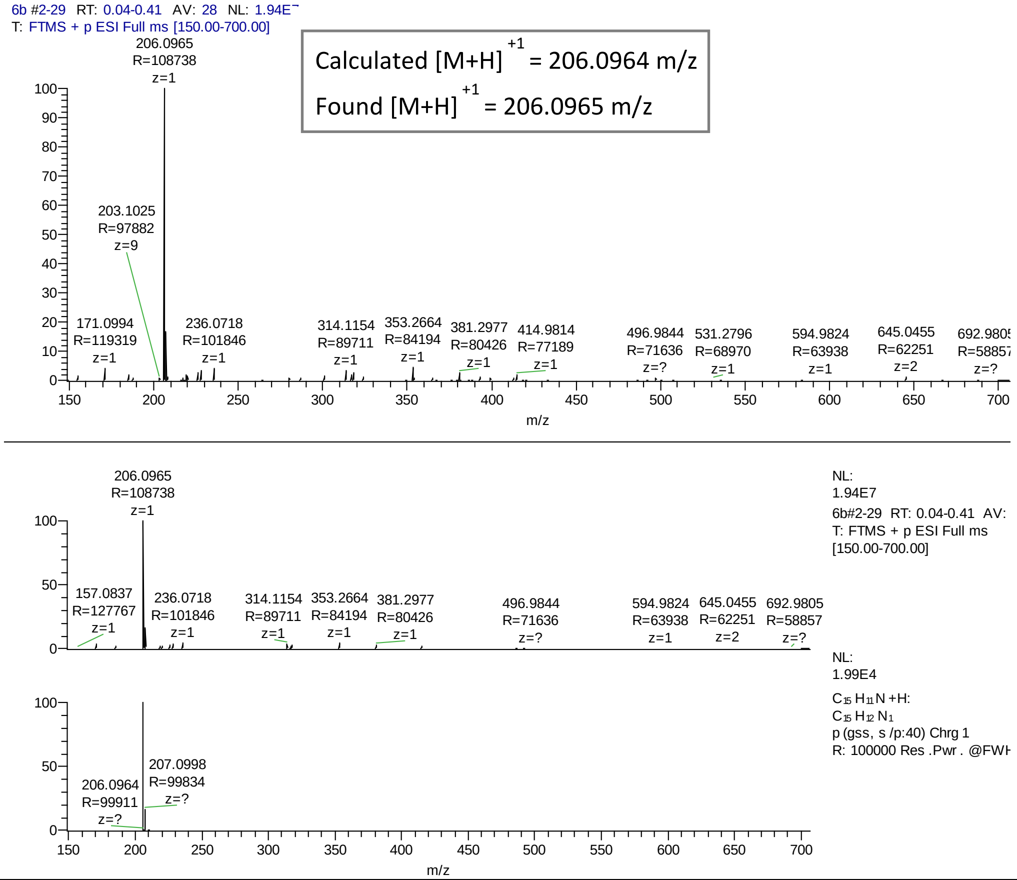

Calculated [M+H]$^{+1}$ = 206.0964 m/z

Found [M+H]$^{+1}$ = 206.0965 m/z

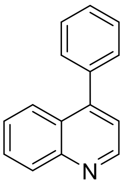

6b

HRMS (7b)

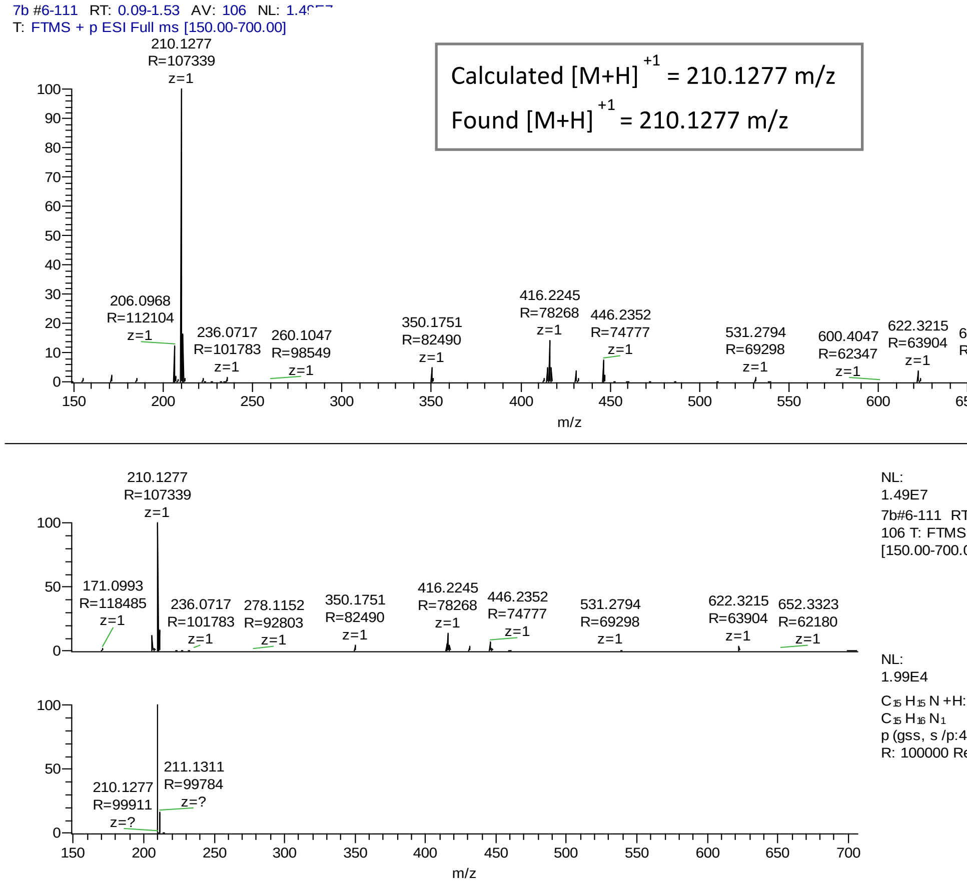

Calculated [M+H]$^{+1}$ = 210.1277 m/z

Found [M+H]$^{+1}$ = 210.1277 m/z

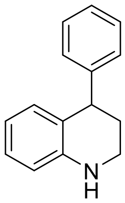

7b
